# WRKY45-dependent priming of diterpenoid phytoalexin biosynthesis in rice and the role of cytokinin in triggering the reaction

**DOI:** 10.1007/s11103-014-0221-x

**Published:** 2014-07-18

**Authors:** Aya Akagi, Setsuko Fukushima, Kazunori Okada, Chang-Jie Jiang, Riichiro Yoshida, Akira Nakayama, Masaki Shimono, Shoji Sugano, Hisakazu Yamane, Hiroshi Takatsuji

**Affiliations:** 1 Disease Resistant Crops Research Unit, National Institute of Agrobiological Sciences, 2-1-2 Kannondai, Tsukuba, Ibaraki 305-8602 Japan; 2 Biotechnology Research Center, The University of Tokyo, 1-1-1 Yayoi, Bunkyo-ku, Tokyo, 113-8657 Japan; 3 Present Address: Bayer CropScience, Tokyo, 100-8262 Japan; 4 Present Address: Faculty of Agriculture, Kagoshima University, Kohrimoto, Kagoshima, 890-0065 Japan; 5 Present Address: Maebashi Institute of Technology, Maebashi, 371-0816 Japan; 6 Present Address: Department of Plant Pathology, Michigan State University, 104 Center for Integrated Plant Systems, East Lansing, MI 48824 USA; 7 Present Address: Department of Biosciences, Teikyo University, Toyosatodai 1-1, Utsunomiya, Tochigi 320-8551 Japan

**Keywords:** Salicylic acid, Benzothiadiazole, Rice, Phytoalexin, Priming, Cytokinin

## Abstract

Plant activators such as benzothiadiazole (BTH) protect plants against diseases by priming the salicylic acid (SA) signaling pathway. In rice, the transcription factor WRKY45 plays a central role in this process. To investigate the mechanism involved in defense-priming by BTH and the role of WRKY45 in this process, we analyzed the transcripts of biosynthetic genes for diterpenoid phytoalexins (DPs) during the rice–*Magnaporthe oryzae* interaction. The DP biosynthetic genes were barely upregulated in BTH-treated rice plants, but were induced rapidly after *M. oryzae* infection in a *WRKY45*-dependent manner. These results indicate that the DP biosynthetic genes were primed by BTH through WRKY45. Rapid induction of the DP biosynthetic genes was also observed after *M. oryzae* infection to *WRKY45*-overexpressing (*WRKY45*-ox) plants. The changes in gene transcription resulted in accumulation of DPs in *WRKY45*-ox and BTH-pretreated rice after *M. oryzae* infection. Previously, we reported that cytokinins (CKs), especially isopentenyladenines, accumulated in *M. oryzae*-infected rice. Here, we show that DP biosynthetic genes are regulated by the SA/CK synergism in a WRKY45-dependent manner. Together, we propose that CK plays a role in mediating the signal of *M. oryzae* infection to trigger the induction of DP biosynthetic genes in BTH-primed plants.

**Electronic supplementary material:**

The online version of this article (doi:10.1007/s11103-014-0221-x) contains supplementary material, which is available to authorized users.

## Introduction

Plants have inducible defense mechanisms, such as systemic acquired resistance (SAR), which provide protection against invading pathogens. SAR is mediated by the salicylic acid (SA) defense-signaling pathway (Sticher et al. 1997; Durrant and Dong 2004; Loake and Grant 2007). Plants can be pre-conditioned to respond to imminent pathogen invasion by previous pathogen infection, root colonization, or chemical treatments; this pre-conditioning for a faster and stronger defense response is known as “priming” (Conrath et al. 2006). Chemical defense inducers, also known as plant activators, such as benzothiadiazole (BTH), as well as exogenously applied SA, induce defense responses through the SA signaling pathway, thereby protecting plants from various biotrophic and hemi-biotrophic plant pathogens (Katz et al. 1998). In a parsley cell culture, for example, BTH treatment did not induce the expression of the *PAL* gene or the accumulation of coumarin, the parsley phytoalexin; however, both were rapidly induced by adding an elicitor to the BTH-pretreated cells (Katz et al. 1998). In Arabidopsis, the priming of defense gene expression by chemical inducers through the SA pathway is associated with accumulation of inactive mitogen-activated protein (MAP) kinases, and their activation is required for defense induction (Beckers et al. 2009). Chromatin modification has also been implicated in priming during SAR (Jaskiewicz et al. 2011). However, further investigations are necessary to fully understand the mechanisms of defense priming in various pathosystems.

In Arabidopsis, the transcriptional coactivator NPR1 plays the major role in the SA-signaling pathway. We have proposed that the SA-signaling pathway in rice branches into OsNPR1- and WRKY45-dependent subpathways (Shimono et al. 2007; Sugano et al. 2010). Both are essential for BTH-induced resistance to phytopathogens such as *Magnaporthe oryzae* and *Xanthomonas oryzae* pv. *oryzae* (*Xoo*), as shown by the largely compromised induced resistances of rice transformants with a silenced *OsNPR1* gene (Chern et al. 2005; Yuan et al. 2007; Sugano et al. 2010) or a silenced *WRKY45* gene (Shimono et al. 2007, 2012). Overexpression of *WRKY45* (*WRKY45*-ox) in rice conferred very strong resistance against both *M. oryzae* and *Xoo* (Shimono et al. 2007, 2012), as did overexpression of *OsNPR1* (Chern et al. 2005; Sugano et al. 2010). Strong disease resistance is often accompanied by severe growth defects because of the high costs of defense reactions (Jaskiewicz et al. 2011); however, *WRKY45*-ox rice plants showed relatively minor growth problems alongside strong disease resistance, although the growth problem occasionally increased due to untimely defense activation triggered by an unknown environmental factor(s) (Shimono et al. 2007). Hence, WRKY45 is a potential candidate for development of rice lines that are resistant to multiple diseases. The strong resistance to *M. oryzae* in *WRKY45*-ox rice consists of a two-layered defense mechanism involving pre- and post-invasive defenses: fungal invasion from more than 95 % of *M. oryzae* appressoria into rice cells was blocked, and post-invasive defense accompanying hypersensitive reaction-like cell death was observed where *M. oryzae* had invaded rice cells (Shimono et al. 2012). Recently, we suggested that ubiquitin–proteasome degradation plays a role in suppressing defense activation in the absence of pathogens (Matsushita et al. 2013). We have also shown that cytokinins (CKs) accumulate in *M. oryzae*-infected leaves and that CK signaling was activated and interacted with the SA pathway (Jiang et al. 2013).

Phytoalexins are defined as low-molecular-weight compounds with antimicrobial activity that are produced by plants in response to infection. They are diverse in structure and different plant species have their own specific phytoalexins (Ahuja et al. 2012; Schmelz et al. 2014). Most phytoalexins are synthesized through the phenylpropanoid-, diterpenoid (DP)-, or tryptophan pathways. In rice, 16 molecular species of phytoalexins have been identified: 15 diterpenoids and one flavonoid. Momilactones and phytocassanes are the best characterized among the diterpenoids, and their antimicrobial activities against *M. oryzae* have been reported. Exogenously supplied momilactone A decreased *M. oryzae* infection of detached rice leaves (Hasegawa et al. 2010), and phytocassanes A–D prevented spore germination and germ-tube growth of *M. oryzae* on slide glass (Koga et al. 1995). Moreover, a momilactone A biosynthetic mutant was impaired in *M. oryzae* resistance (Toyomasu et al. 2014), although another group has reported contradictory results (Xu et al. 2012). The biosynthetic pathways of these phytoalexins and the genes involved in them have been reported. Copalyl diphosphate synthase (CPS) 4, kaurene synthase-like (KSL) 4, cytochromes P450 monooxygenases (CYP) 99A2 and CYP99A3, and momilactone A synthase (MAS), which are encoded in a gene cluster on chromosome 4 (Shimura et al. 2007), are involved in the biosynthesis of momilactones. CPS2, KSL7, CYP71Z7, CYP76M7 and CYP76M8 whose genes are organized in a gene cluster on chromosome 2 (Swaminathan et al. 2009; Wu et al. 2011; Wang et al. 2012b), are involved in the biosynthesis of phytocassanes A–E. KOL4/CYP701A8, an *ent*-kaurene oxidase paralog, is involved in the synthesis of oryzalexins A–C and E and phytocassanes A–E (Wang et al. 2012a).

Production of phytoalexins is regulated by defense signaling pathways mediated by various plant hormones in response to infection by different pathogens. In Arabidopsis, production of camalexin during *Botrytis cinerea* infection was jasmonic acid-dependent (Rowe et al. 2010), whereas its accumulation after *Alternaria brassicicola* infection was independent of jasmonic acid (Thomma et al. 1999; van Wees et al. 2003). SA-independent camalexin production was observed during infection by *Phytophthora porri* (Roetschi et al. 2001) and *Pseudomonas syringae* DC3000 (Nawrath and Metraux 1999). Arabidopsis WRKY33 is implicated in regulating camalexin production downstream of different MAP kinase cascades upon infection by different plant pathogens (Qiu et al. 2008; Mao et al. 2011). Transgenic tobacco plants with increased CK levels displayed enhanced resistance to virulent *P. syringae* pv. *tabaci* through up-regulated syntheses of two major antimicrobial phytoalexins, scopoletin and capsidiol (Grosskinsky et al. 2011). In rice, sakuranetin, a phenylpropanoid phytoalexin, accumulated in leaf discs in response to jasmonic acid (Tamogami et al. 1997). Momilactones and phytocassanes have been reported to accumulate after CK treatments (Ko et al. 2010). A MAPK cascade involving MKK4 was shown to regulate DP biosynthetic genes in response to a chitin elicitor signal (Kishi-Kaboshi et al. 2010). Recently, rice WRKY53 has been shown to be phosphorylated by this MAPK cascade and positively regulate the DP biosynthetic genes (Chujo et al. 2014). Meanwhile, rice WRKY76 negatively regulates the DP biosynthesis (Yokotani et al. 2013).

In this study, we analyzed the transcript levels of defense genes in BTH-treated and *WRKY45*-ox rice plants before and after *M. oryzae* inoculation. We found that the genes encoding enzymes involved in DP biosynthesis are regulated in a WRKY45-dependent ‘priming’ manner. We also showed that CK signaling plays a pivotal role in triggering the activation of DP biosynthetic genes by acting synergistically with the SA pathway. Based on these data, we discuss possible role of DPs in WRKY45-dependent *M. oryzae* resistance in rice. We also discuss the possible involvement of the synergistic interaction between the SA and CK signaling pathways in triggering DP biosynthesis upon infection of primed rice by *M. oryzae*.

## Results

### Diterpenoid phytoalexin biosynthetic genes were upregulated in *WRKY45*-ox rice plants

**Fig. 1:**
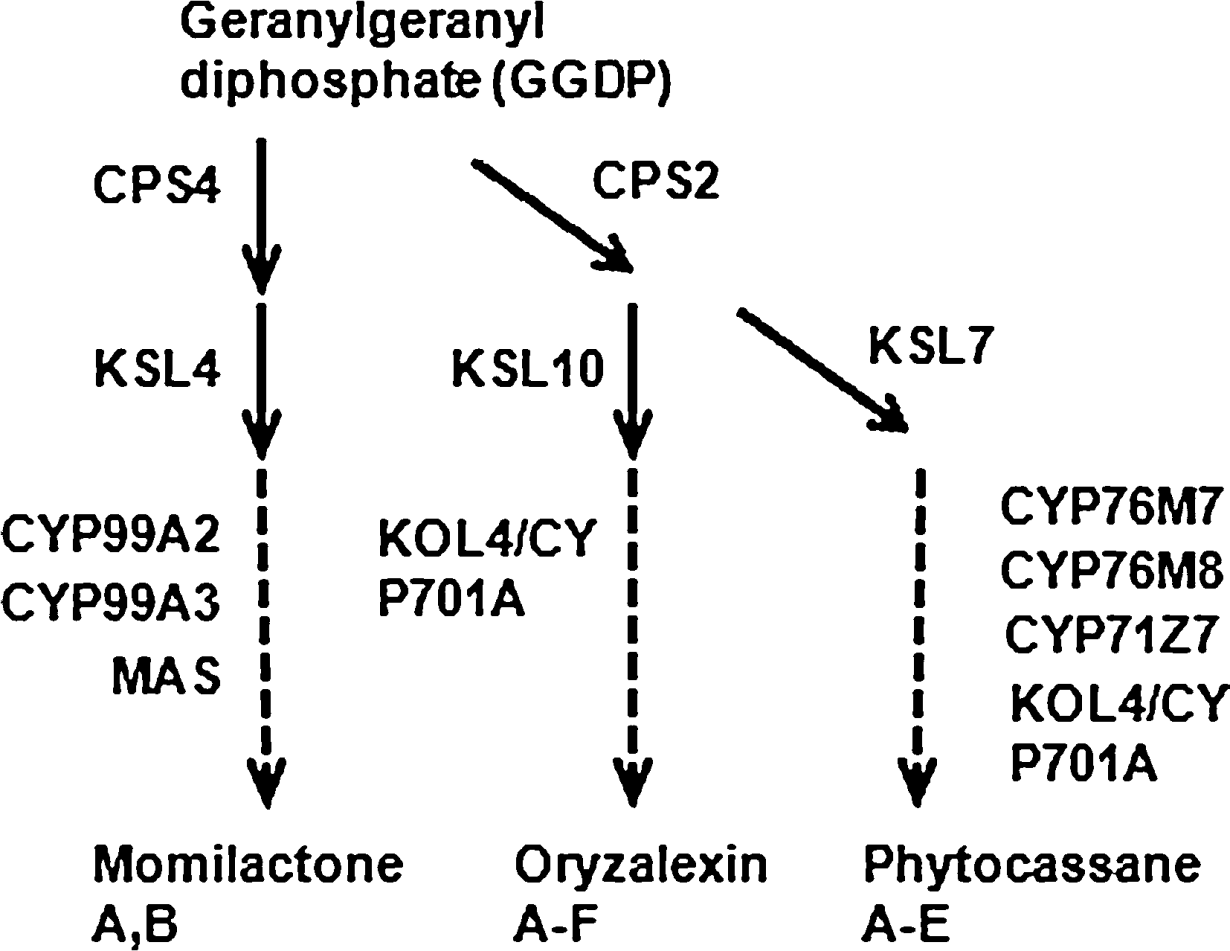
Biosynthetic pathway of diterpenoid phytoalexins in rice

**Fig. 2:**
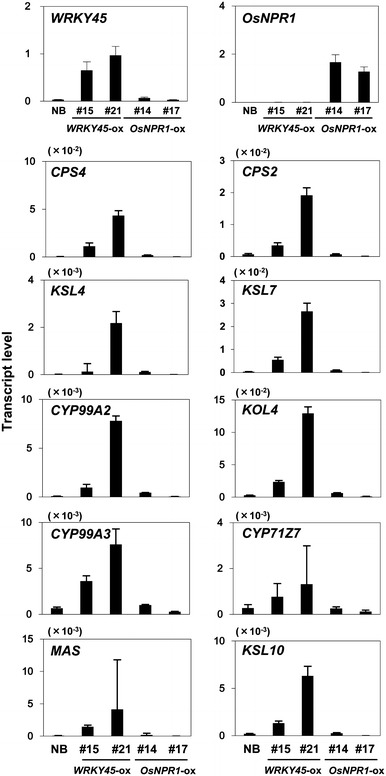
Transcript levels of DP biosynthetic genes in *WRKY45*-ox and *NPR1*-ox rice lines. Transcript levels of biosynthetic genes for momilactones (*CPS4*, *KSL4*, *CYP99A2*, *CYP99A3*, and *MAS*), phytocassanes (*CPS2*, *KSL7*, *KOL4*, and *CYP71Z7*), and oryzalexins (*KSL10*) in *WRKY45*-ox (#15 and #21) and OsNPR1 (#14 and #17) lines were determined by qRT-PCR. RNAs were extracted from fourth leaves of three rice seedlings per line at the four-leaf stage. Means of three technical repeats are shown with standard deviations (SD). We obtained similar results in another independent experiment

Rice plants expressing *WRKY45* under the control of the maize ubiquitin promoter are extremely resistant to rice blast and bacterial leaf-blight diseases. To search for genes potentially responsible for the strong disease resistance conferred by WRKY45 overexpression, we performed genome-wide gene expression analysis of *WRKY45*-ox rice, identifying 1,664 genes that were upregulated in *WRKY45*-ox rice compared with non-transformed rice cv. Nipponbare (NB) (one sample *t* test with false discovery rates <10 % and >twofold changes). Of these, 329 (19 %) were BTH responsive (Table S1) (Shimono et al. 2007). Analysis of the genes with altered expression in *WRKY45*-ox rice by a Gene ontology program (Agri GO; http://bioinfo.cau.edu.cn/agriGO/_) revealed that several genes in the biosynthetic pathway for DPs (Fig. 1), which are known to have anti-blast fungus activities (Hasegawa et al. 2010), were upregulated in *WRKY45*-ox rice. Specifically, five genes encoding enzymes involved in momilactone biosynthesis (*CPS4*, *KSL4*, *CYP99A2*, *CYP99A3*, and *MAS*) were upregulated in *WRKY45*-ox rice, with *KSL4* the most upregulated (11.3-fold). These results prompted us to examine the transcript levels of DP biosynthetic genes more extensively in two lines each of *WRKY45*-ox and *OsNPR1*-ox rice by qRT-PCR (Fig. 2). In addition to the five momilactone biosynthetic genes, those encoding the enzymes for phytocassane biosynthesis (*CPS2*, *KSL7*, *KO4*, and *CYP71Z7*) and oryzalexin biosynthesis (*KSL10*) were also upregulated in both *WRKY45*-ox rice lines. By contrast, none of these genes were affected by the overexpression of *OsNPR1*. We have recently reported on WRKY45-dependent BTH-responsive genes identified by a comprehensive gene expression analysis using *WRKY45*-knockdown rice; however, the DP biosynthetic genes were not identified as WRKY45-dependent genes because they were not induced by BTH in this experiment (Shimono et al. 2007; Nakayama et al. 2013). On the other hand, we previously reported that gene expression patterns in WRKY45-ox rice varied depending on growth conditions (Shimono et al. 2007). In light of these observations, an additional factor(s), presumably an environmental factor(s), acted to induce the WRKY45-dependent upregulation of DP genes described above.

**Fig. 3:**
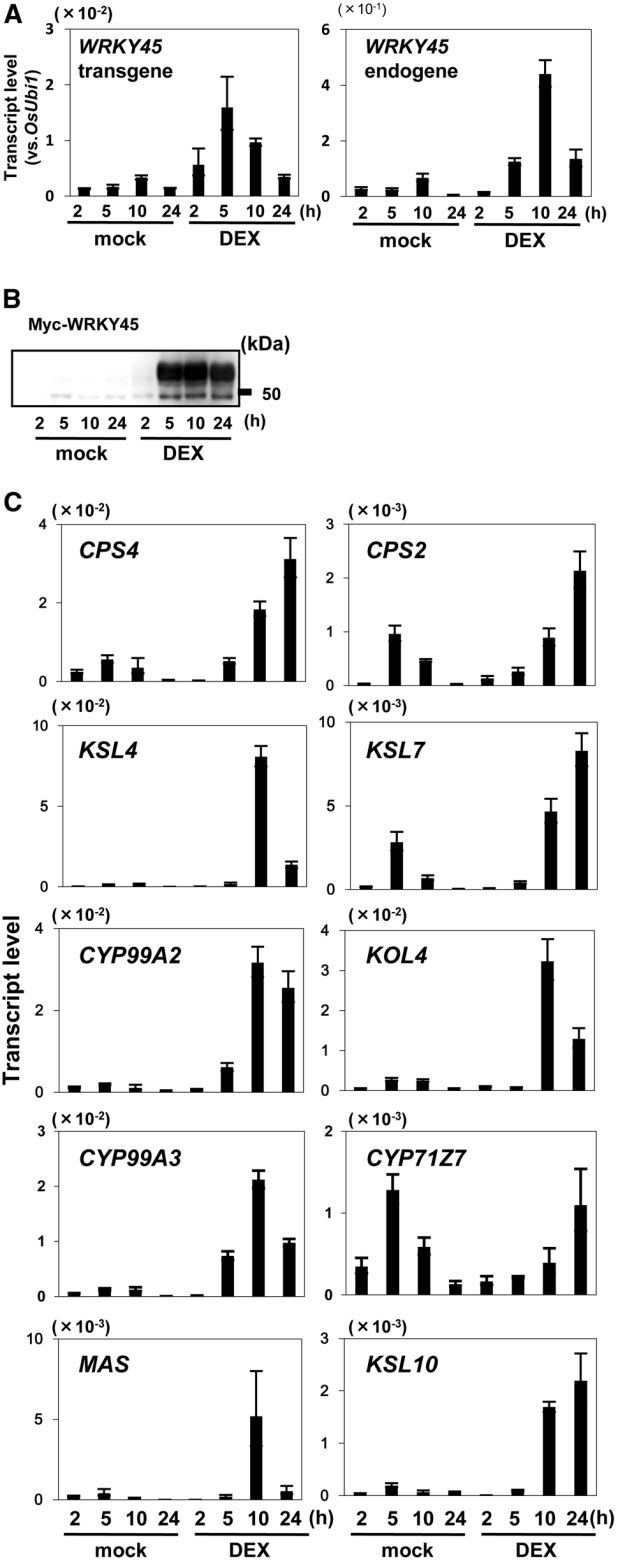
Upregulation of DPs biosynthetic genes by DEX-induced WRKY45. **A** and **B** Induction of WRKY45. *GVG*-*WRKY45*-*myc* transformants at the three-leaf stage were treated with DEX to induce *WRKY45*-*myc*. Whole seedlings were harvested at 2, 5, 10, and 24 h after treatments. Transcript levels of *WRKY45* transgene and *WRKY45* endogenous gene (**A**) were determined by qRT-PCR using specific primers for *rbcs*-*3A* terminator (T3A) and *WRKY45* 3′-UTR sequences, respectively. Protein levels of WRKY45-myc (**B**) were determined by western blotting using anti-myc antibody. **C** Transcript levels of DP biosynthetic genes. Transcript levels of genes involved in the biosynthesis of momilactones (*CPS4*, *KSL4*, *CYP99A2*, *CYP99A3*, and *MAS*), phytocassanes (*CPS2*, *KSL7*, *KOL4*, and *CYP71Z7*), and oryzalexins (*KSL10*) were determined by qRT-PCR. Means of three determinations are shown with SD

To validate the WRKY45-dependence of induction of DP biosynthetic genes, we analyzed gene transcript levels in GVG-WRKY45-myc transgenic rice plants, in which the expression of myc-tagged WRKY45 can be induced by dexamethazone (DEX, Fig. 3). Transcript levels of the transgene-derived *WRKY45* began to increase at 2 h and peaked at 5 h after adding DEX. This was followed by increased transcription of endogenous *WRKY45* as a result of autoregulation (Nakayama et al. 2013); its transcript level began to increase at 5 h and peaked at 10 h (Fig. 3a). Accumulation of WRKY45 proteins was observed 5 h after DEX addition and thereafter (Fig. 3b). Transcript levels of genes encoding enzymes involved in momilactone (*CPS4*, *KSL4*, *CYP99A2*, *CYP99A3*, and *MAS*), phytocassane (*CPS2*, *KSL7*, *KOL4*, and *CYP71Z7*), and oryzalexin (*KSL10*) biosynthesis began to increase after the accumulation of WRKY45 protein. Their transcript levels peaked at 10–24 h after DEX addition (Fig. 3c). Although *CYP71Z7* was upregulated in *WRKY45*-ox rice plants (Fig. 2), its transcript levels did not increase after DEX-induced expression of WRKY45.

### BTH primed the transcription of DP biosynthetic genes via WRKY45

**Fig. 4:**
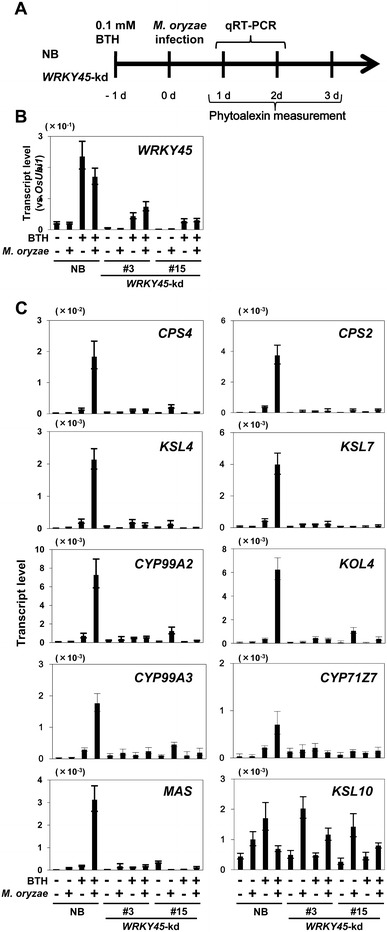
Priming of momilactone and phytocassane biosynthetic genes by BTH via WRKY45. **A** Experimental scheme. Nipponbare (NB) and *WRKY45*-kd lines were treated with BTH and/or spray-inoculated with *M. oryzae* (1.0 × 10^5^ conidia/ml) at four-leaf stage. Transcript levels of DP biosynthetic genes were determined using fourth leaves harvested and pooled from three seedlings in each treatment/line. Transcript levels of *WRKY45* (**B**) and DP biosynthetic genes (**C**) at 1 dpi were determined by qRT-PCR after treatments. Means of three determinations are shown with standard deviations (SD). We obtained similar results in another independent experiment

In our previous study, BTH treatment did not induce DP biosynthetic genes (Shimono et al. 2007; Matsushita et al. 2013). On the other hand, overexpression of *WRKY45* was sufficient to increase the expression of DP biosynthetic genes under our present condition (Figs. 2, 3). A possibility that could account for this inconsistency is that BTH treatment primed the DP biosynthetic genes for expression but required another cue, which can also be provided by environments, to trigger their transcriptional activation. To test this possibility, we inoculated *M. oryzae* onto NB rice plants with or without BTH pre-treatment and analyzed the transcript levels of DP biosynthetic genes at 1 and 2 dpi (Fig. 4a). The transcription of *WRKY45* was induced by BTH treatment alone at 1 dpi (Fig. 4b). By contrast, transcript levels of DP biosynthetic genes were barely upregulated at 1 dpi in the BTH-treated plants, consistent with our previous observations (Shimono et al. 2007). Their transcript levels were not upregulated in *M. oryzae*-inoculated plants without BTH pretreatment, either. Interestingly, however, they were upregulated in BTH-pretreated and *M. oryzae*-inoculated plants at 1 dpi (Fig. 4c). At 2 dpi, the DP biosynthetic genes were induced in *M. oryzae*-inoculated plants even without BTH pretreatment (Fig. S1). Thus, BTH pretreatment increased the speed of induction of DP biosynthetic genes by *M. oryzae* infection, which meets the definition of ‘priming’.

We examined the WRKY45-dependence of the BTH-induced priming by analyzing the expression of DP biosynthetic genes in two lines of *WRKY45*-kd rice after the same set of treatments (Fig. 4). The induction of DP biosynthetic genes at 1 dpi by BTH pretreatment + *M. oryzae* infection was negated in *WRKY45*-kd rice plants, indicating that the priming of DP biosynthetic genes by BTH-treatment is WRKY45 dependent. At 2 dpi, the DP genes were induced by *M. oryzae* infection only, and the induction was independent of WRKY45, suggesting that some other pathway(s) regulates the induction of DPs at this later time point (Fig. S1). In *M. oryzae*-infected BTH-treated NB rice, the accumulation of momilactones and phytocassanes at 3 dpi was enhanced by BTH pretreatment (Fig. S2).

### DP biosynthesis was primed in *WRKY45*-ox rice

**Fig. 5:**
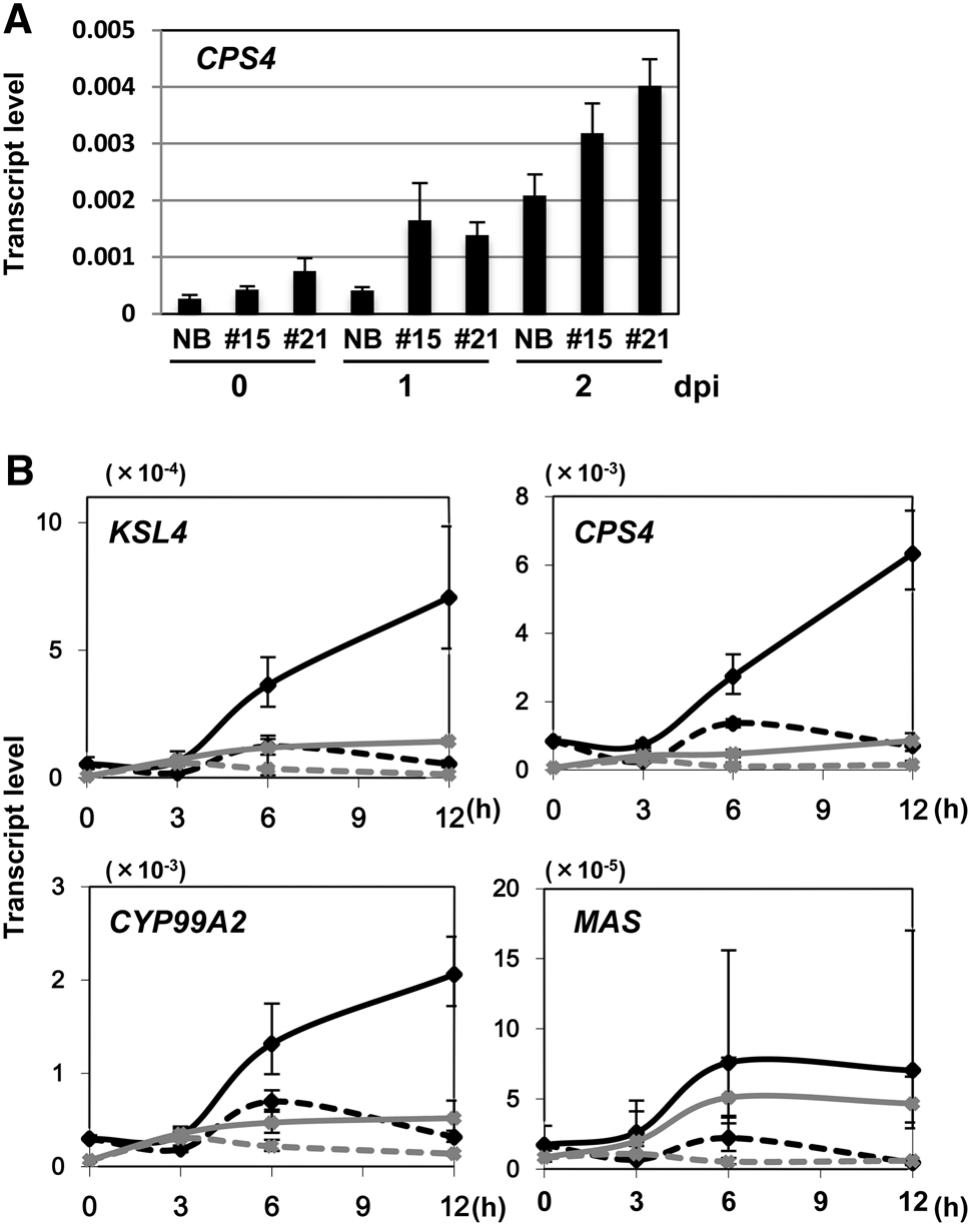
Upregulation of momilactone biosynthetic genes in *WRKY45*-ox rice after *M. oryzae* infection. **A** Nipponbare (NB) and *WRKY45*-ox (#15 and #21) plants were inoculated with *M. oryzae* conidia (10^5^/ml) and the transcript levels of *CPS4* in fifth leaves from three seedlings each were determined by qRT-PCR. **B** Nipponbare (*gray lines*) and *WRKY45*-ox (#21, *black lines*) plants were inoculated with *M. oryzae* conidia (*solid lines*, 10^5^/ml) or mock-treated by spraying solvent only (*dashed lines*) at four-leaf stage. Transcript levels of momilactone biosynthetic genes (*CPS4*, *KSL4*, *CYP99A2*, and *MAS*) in fifth leaves from three seedlings in each treatment/line were determined by qRT-PCR.Means of three determinations are shown with SD. All the experiments were performed twice independently and obtained similar results

We analyzed the transcript levels of DP biosynthetic genes in *WRKY45*-ox rice plants after *M. oryzae* infection (Fig. 5). The differences in the expression levels of *CPS4* between *WRKY45*-ox rice lines and NB were small before inoculation but enlarged at 1 dpi (Fig. 5a). During earlier phases of infection, the transcripts of momilactone biosynthetic genes were increased after *M. oryzae* infection as early as 6 hpi, while no such induction was observed in NB (Fig. 5b). These results indicate that the DP biosynthetic genes are primed in *WRKY45*-ox rice without BTH pretreatment.

**Fig. 6:**
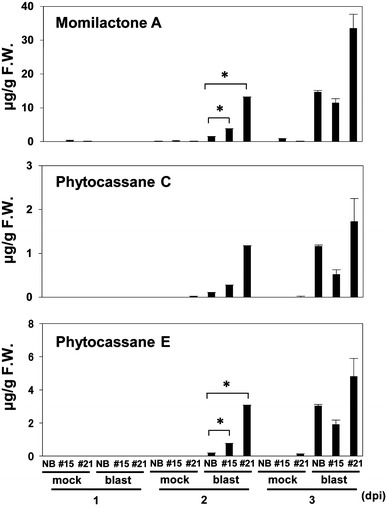
Accumulation of momilactones and phytocassanes in response to *M. oryzae* infection in *WRKY45*-ox rice plants.Plants at four-leaf-stage were spray-inoculated with conidia of *M. oryzae* (1.5 × 10^5^/ml). Fourth leaves from the seedlings in each treatment were harvested into six pools of three plants each (0.05 g) at each time point. DPs were determined for the replicate samples and means are shown with standard errors (SE). *p < 0.05 in student’s *t* test

To determine whether the increased transcript levels of these genes resulted in accumulation of DPs, we analyzed the DP contents in 70 % methanol extracts from leaves of *M. oryzae*-infected and -uninfected *WRKY45*-ox and NB plants by LC–MS/MS (Fig. 6). At 1 dpi, DPs were barely detectable in any sample. At 2 dpi, momilactone A and phytocassanes E had accumulated to higher levels in *M. oryzae*-infected *WRKY45*-ox plants than in *M. oryzae*-infected NB. At 3 dpi, *M. oryzae*-infected NB accumulated levels of DPs comparable to those in *WRKY45*-ox rice. High levels of sakuranetin accumulate in rice during the compatible interaction with *M. oryzae* (Kodama et al. 1992; Jung et al. 2005). However, we did not detect sakuranetin accumulation in either NB or *WRKY45*-ox rice plants in our experimental conditions.

### SA and CK synergistically upregulated DP biosynthetic genes

**Fig. 7:**
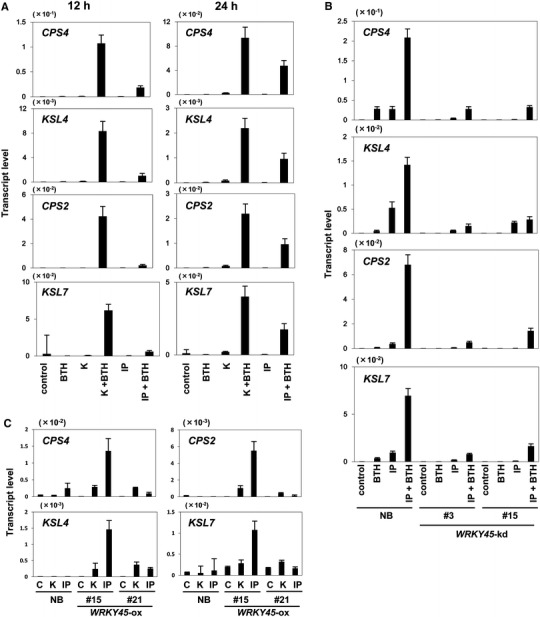
Synergism between SA and CK in inducing DP biosynthetic genes. **A** Induction of DP biosynthetic genes in rice by co-treatments of CK and SA. Roots of Nipponbare rice plants were supplied with kinetin or IP (50 µM) and/or BTH (90 µM) for 12 or 24 h. Transcript levels of DP biosynthetic genes were determined by qRT-PCR. **B** WRKY45 dependence of induction of DP biosynthetic genes by CK/SA synergism. Nipponbare (NB) and *WRKY45*-kd rice plants were treated with an aqueous solution containing 50 µM IP and/or 90 µM BTH from roots for 8 h. Then, transcript levels of DP biosynthetic genes were determined by qRT-PCR. **C** Induction of DP biosynthetic genes in *WRKY45*-ox rice triggered by CK treatment. Nipponbare (NB) and *WRKY45*-ox rice lines were treated with 50 µM kinetin (K) or IP for 12 h. Transcript levels of DP biosynthetic genes were determined by qRT-PCR. Means of three determinations are shown with SD. We obtained similar results in another independent experiment

The activation of DP biosynthetic genes triggered by *M. oryzae* inoculation in BTH-pretreated NB and in *WRKY45*-ox rice plants raises the question of how the signal of *M. oryzae* infection is mediated. CK was reported to induce the expression of DP biosynthetic genes and the accumulation of their products in rice (Ko et al. 2010). Recently, we showed that the synthetic CK kinetin acts synergistically with the SA signaling pathway to induce *PR* gene expression in rice (Jiang et al. 2013). In addition, N6-(Δ-isopentenyl) adenine (IP) was reported to accumulate in *M. oryzae*-infected rice leaves (Jiang et al. 2013). These observations led us to test the effect of CKs on transcription of DP biosynthesis genes to explore the mechanism underlying their activation upon *M. oryzae* infection in BTH-primed rice plants. Here, we chose two genes each for momilactone (*CPS4* and *KSL4*) and phytocassane (*CPS2* and *KSL7*) biosyntheses to analyze their expression as representatives. BTH treatment alone did not result in increased transcript levels of DP biosynthesis genes in rice leaves (Fig. 7a). Kinetin or IP alone induced their transcription only slightly; however, co-treatment of BTH and CKs strongly induced transcription of all the DP biosynthetic genes tested (Fig. 7a). This result indicated that there is a synergistic relationship between BTH and CKs in their transcriptional activation. In these samples, *PR1b* transcription was also induced by co-treatments of BTH and CKs, while transcription of *WRKY45* was induced by BTH alone, and that of *OsRR6* was induced by CKs alone (Fig. S3). Interestingly, transcription of *PR1b* and DP biosynthetic genes was induced earlier by a kinetin and BTH co-treatment than by IP and BTH (Fig. 7a and Fig. S3). The strong induction of these genes by the SA/CK co-treatment was largely compromised in *WRKY45*-kd rice plants, indicating the WRKY45 dependence of this regulation (Fig. 7b). In *WRKY45*-ox rice plants, DP biosynthetic genes were induced by CKs (kinetin and IP) even without BTH (Fig. 7c). Thus, CK treatment appears to mimic *M. oryzae* infection of rice plants in which the SA pathway is primed (Fig. 5), suggesting a role of CK signaling in the WRKY45-dependent regulation of DP biosynthetic genes.

## Discussion

### WRKY45 plays a central role in the priming of DP biosynthetic genes

The extremely strong resistance of *WRKY45*-ox rice plants to *M. oryzae* in spite of relatively minor effects on plant growth (Shimono et al. 2007) prompted us to explore the mechanism regulating their disease resistance. Priming of disease resistance responses is one of the strategies to mobilize rapid and strong defense responses to protect plants against pathogens (Conrath et al. 2006). Our data indicated that WRKY45 is a key player in the priming of DP biosynthesis in rice. Induction of DP biosynthetic genes in rice after *M. oryzae* infection was advanced by BTH pretreatment, compared with non-treated rice, in a *WRKY45*-dependent manner (Fig. 4). The DP biosynthetic genes were also primed in *WRKY45*-ox rice (Fig. 5).

Different levels of upregulation of DP biosynthetic genes were observed even without *M. oryzae* infection in *WRKY45*-ox rice plants (Figs. 2, 3, 5). This upregulation probably corresponds to the “direct defense” previously reported in parsley cells (Thulke and Conrath 1998): *PAL* expression was primed after low-dosage SA application, but directly induced after high-dosage SA application. Presumably, one of the factors that determine whether priming or direct defense is induced in our system is the level of *WRKY45* expression. However, an environmental factor(s) also appear to influence the outcomes. The DP biosynthetic genes were induced substantially in *WRKY45*-ox and *GVG*-*WRKY45* rice (Figs. 2, 3) but only marginally in BTH-treated rice (Fig. 4), while *WRKY45* transcript levels were similar in these plants. The effects of environmental factors that triggered the expression of DP genes in *WRKY45*- or BTH-primed plants seem likely to underlie these observations.

### Contribution of rice DPs to *WRKY45*-induced defense responses

Strong resistance to *M. oryzae* due to pre- and post-invasive defense mechanisms was observed in *WRKY45*-ox rice (Shimono et al. 2012). How do DPs contribute to *M. oryzae* resistance in *WRKY45*-ox rice? *M. oryzae* invasion occurs at around 24 hpi (Kankanala et al. 2007). The high level of DP accumulation detected at 2 dpi and thereafter (Fig. 6) could be responsible for post-invasive defense. The transcript levels of DP biosynthetic genes began to increase at 6 h after *M. oryzae* infection, long before the invasion of *M. oryzae* into rice cells (Fig. 5). However, DPs began to accumulate much later (2 dpi). This time lag could be explained by the sensitivity of DPs detection. If accumulation of DPs is restricted to a very small area around infection sites in the early phases of blast fungus infection, they would be difficult to detect even if their local levels were high. Based on this speculation, it is possible that the *M. oryzae*-induced phytoalexins in *WRKY45*-ox rice contribute to preinvasive defense, which seems to occur at the infection sites at a very early phase. Thus, in *WRKY45*-ox rice plants, *M. oryzae*-induced DPs can contribute to pre-invasive defense, post-invasive defense, or both.

### DP biosynthetic genes are regulated by multiple signaling pathways

Transcriptional regulation of DP biosynthetic genes is rather complicated. The transcription factor TGAP1 has been implicated in transcriptional activation of momilactone biosynthetic genes in rice in response to the elicitor chitin (Okada et al. 2009). A MAP kinase component, MKK4, plays a role in chitin-elicited activation of genes involved in the biosynthesis of momilactones and phytocassanes (Kishi-Kaboshi et al. 2010). Rice WRKY53 positively regulates the DP biosynthetic genes downstream of this MAPK cascade and WRKY76 negatively regulates them (Yokotani et al. 2013; Chujo et al. 2014). Is there any regulatory linkage between WRKY45 and these regulators? *WRKY45* was not transcriptionally upregulated by MKK4 (Kishi-Kaboshi et al. 2010). Our data indicated that *TGAP1* expression was not responsive to BTH or to DEX-induced expression of *WRKY45* (Fig. S4), suggesting that TGAP1 is not involved in the SA pathway. These data suggest that the DP biosynthetic genes are regulated by more than one independent signaling pathway; the chitin-elicited pathway mediated by MKK4 and the SA pathway mediated by WRKY45. At 2 dpi, the DP biosynthetic genes were upregulated in NB by *M. oryzae* infection only. This upregulation was not affected by *WRKY45* knockdown (Fig. S1), consistent with the presence of the WRKY45-independent pathway.

In Arabidopsis, *P. syringae*-induced MPK4-dependent regulation of camalexin biosynthetic genes is mediated by WRKY33 (Qiu et al. 2008). WRKY33 also mediates *B. cinerea*-induced MPK3/MPK6-dependent camalexin production (Mao et al. 2011). While rice WRKY45 is of group III, Arabidopsis WRKY33 belong to group I (Eulgem et al. 2000). *B. cinerea*-induced camalexin biosynthesis is jasmonic acid-dependent (Rowe et al. 2010) and SA-independence was reported for camalexin biosynthesis induced by different pathogens (Nawrath and Metraux 1999; Roetschi et al. 2001). Thus, there are obvious differences between WRKY45-regulated DP biosynthesis in rice and WRKY33-regulated camalexin biosynthesis in Arabidopsis.

### Role of CK signaling in mediating the *M. oryzae* infection signal that regulates DP biosynthetic genes

CK alone at concentrations up to 100 μM induced DP synthetic genes (Fig. 7) as well as DP production (Ko et al. 2010) in rice. On the other hand, higher concentrations of CKs reduced the DP levels (Ko et al. 2010), presumably due to free-radical-scavenging effects of CKs at these concentrations (Ko et al. 2010). Consistent with this, high doses of CKs (110–460 μM) increased blast disease incidence by 18–64 % (Matsumoto 1980). Thus, CKs appears to affect DP production and disease resistance in a dose-dependent manner. Our results show that 100 μM CKs treated together with SA had several-fold greater effects on the induction of DP genes than that of CK alone (Fig. 7), indicating an importance of this synergistic crosstalk of signalings at least at this concentration.

**Fig. 8:**
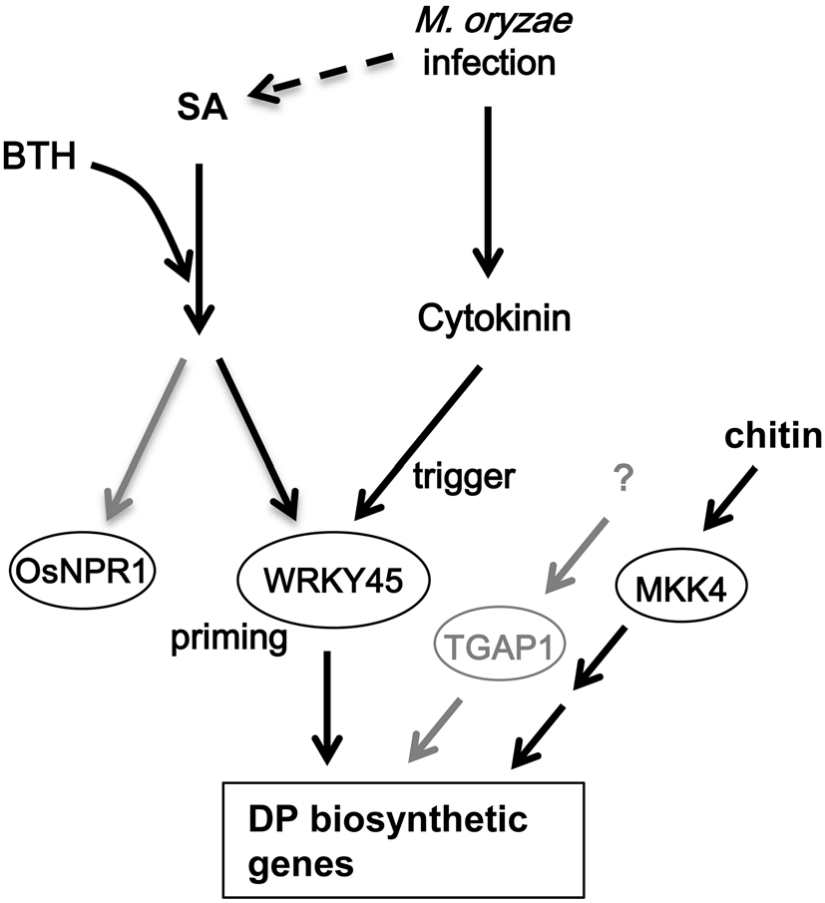
Proposed model for WRKY45-dependent regulation of DP biosynthesis. BTH acts on the rice SA signaling pathway, which is split into WRKY45- and OsNPR1 subpathways. BTH primes the expression of DP biosynthetic genes via WRKY45. *M. oryzae* infection triggers early accumulation of CKs, which in turn act synergistically with the WRKY45-mediated SA signaling pathway to activate expression of DP biosynthetic genes, leading to accumulation of DPs. MKK4-dependent pathways are also shown

Induction of DP biosynthetic genes was triggered by *M. oryzae* inoculation in BTH-pretreated NB and in *WRKY45*-ox rice plants (Figs. 4c, 5, 8). We reasoned that another signaling pathway activated by fungus infection could be involved in regulating this phenomenon. Previously, we showed that there is a synergistic effect of CK and SA on upregulation of the defense gene *PR1b* (Jiang et al. 2013). In this study, we found that DP biosynthetic genes were also highly activated by a co-treatment of SA/BTH and CKs in a WRKY45-dependent manner (Fig. 7). Both the synthetic CK (kinetin) and the natural CK (IP) showed synergistic effects with BTH to upregulate transcription of DP biosynthetic genes, as well as *PR1b*. In *WRKY45*-ox rice plants, the CKs triggered the transcription of DP biosynthetic genes in the absence of exogenous SA or BTH. Previously, we showed that CKs, particularly IP and its precursors, accumulated in the *M. oryzae*-infected area in rice leaves, although it was unclear whether they were derived from the fungal body or whether their production was induced in plant cells upon fungus infection (Jiang et al. 2013). A reporter assay using a CK-responsive reporter gene indicated that CK signaling was activated around disease lesions (Jiang et al. 2013). In Arabidopsis, it has been reported that CK signaling acts together with SA signaling, thereby enhancing plant immunity against *P. syringae* pv. *tomato* DC3000 and *Hyaloperonospora arabidopsis* isolate Noco2, through the cooperative actions of the transcription factors TGA3 and ARR2, which mediate the SA- and CK-signaling pathways, respectively (Choi et al. 2010; Argueso et al. 2012). Taken together, these results and observations suggest that CK signaling, which was activated after *M. oryzae* infection through increased CK levels in plants, activates WRKY45 possibly post-translationally, thereby triggering transcription of the DP biosynthetic genes that had been primed by the SA pathway through WRKY45 (Fig. 8). We propose that this interaction between signaling pathways could underlie the mechanism of defense priming by chemical inducers and *WRKY45* overexpression. Our results also suggested the presence of WRKY45-independent pathway that regulates DP biosynthetic genes during late phase of *M. oryzae* infection (Fig. S1). It is possible that TGAP1 and/or MKK4 mediates this pathway (Fig. 8).

## Materials and methods

### Biological material

Rice transformants (*Oryza sativa* cv. NB) of *WRKY45*-ox and -kd (Shimono et al. 2007), *OsNPR1*-ox (Sugano et al. 2010), and *GVG*-*WRKY45*-*myc* were grown in a greenhouse in soil (Bonsol No.2; Sumitomo Chemical corp., Tokyo, Japan, http://www.sumitomo-chem.co.jp/) under 28 °C days and 23 °C nights.

### Chemical treatments

For chemical applications, rice seeds were germinated on MS plates. Seedlings at the three-leaf stage were transferred to a plastic box (6.5 cm × 6.5 cm × 10 cm) containing 15 ml 1/1000 hyponex (HYPONex Japan, Osaka, Japan; http://www.hyponex.co.jp/) and kept in a growth chamber for 2 days. For DEX treatment, DEX solution (10 mM in 0.1 % DMSO) or the solvent (mock) was added to the hyponex solution to a final DEX concentration of 10 µM. Three whole seedlings were sampled and pooled for analysis. For CK and SA treatments, kinetin or IP and/or BTH were added to the hyponex solution to final concentrations of 50 μM (CKs) and 90 μM (BTH). After addition of the chemicals, the plastic box was incubated in the light at 30 °C.

### *Oryzae* inoculation

A compatible race of blast fungus *M. oryzae* (race 007.0) was cultured and inoculated as described previously (Jiang et al. 2009) with some modifications. In brief, *M. oryzae* was grown on oatmeal agar plates at 26 °C for 10–12 days. After removal of fungal hyphae by brushing and washing with distilled water, the plates were kept under black-blue light (FL15BLB; Toshiba, Osaka, Japan, http://www.toshiba.co.jp) for 3 days at 24 °C to induce conidia formation. Rice seedlings at the four-leaf stage were spray-inoculated with *M.* *oryzae* conidia suspensions [1.000A0× 10^5^ (summer) or 1.5 × 10^5^ (winter)] in 0.01 % Tween 20, kept in a dew chamber 513A (Ozawa corporation, Kyoto, Japan) at 24 °C for 20–24 h, and then moved to a greenhouse.

### Microarray analysis

NB and *WRKY45*-ox rice plants were grown in a greenhouse. The fully expanded youngest leaves from three rice seedlings at the five-leaf-stage were harvested and pooled in three replicates. Total RNAs were extracted and their integrity was checked with a Bioanalyzer (Agilent Technologies Inc., http://www.home.agilent.com). The RNAs (400 ng each) were used to produce double-stranded cDNAs and Cy3-labeled cRNAs were synthesized from the cDNAs using T7 RNA polymerase. The cRNAs were purified, fragmented, and hybridized to an Agilent Rice Oligo Microarray (44 K, custom-made, Agilent technologies). After hybridization, microarray slides were scanned (scanner model G2505B; Agilent Technologies) and data were extracted using Feature Extraction software (Agilent Technologies). Data from three biological replicate experiments were normalized to the mean of several ubiquitin genes (Os01g0328400, Os01g0918200, Os01g0918300, Os02g0161900, Os02g0628800, Os04g0580400, Os05g0160200, Os06g0650100, Os06g0681400, Os09g0420800, Os09g0483400, Os11g0145400, Os12g0143100) by the Subio Platform (Subio Inc., http://www.subio.jp/) and calculated to determine -fold changes (*WRKY45*-ox vs. NB) using one sample *t* test with a 10 % false discovery rate (q value). The gene ontology program Agri GO (http://bioinfo.cau.edu.cn/agriGO/) was used to deduce the biological processes affected by *WRKY45* overexpression.

### Plasmid construction and plant transformation

The plasmid for driving DEX-induced expression of myc-tagged WRKY45 proteins in rice cells was constructed as follows: the CDS of *WRKY45* was amplified by PCR with the primers *Xho*I-WRKY45FW (5′-CTCGAGATGACGTCATCGATGTC-3′) and *Bam*HI-WRKY45RV (5′-GGATCCAAAGCTCAAACCCATAATG-3′). The CDS was then inserted into the pGEM-T Easy Vector (Promega, www.promega.com/) to generate pGEM-WRKY45, which contained the *WRKY45* CDS and a *BamH*I site between *Xho*I and *Sac*I sites. A DNA fragment encoding three tandemly repeated *myc* sequences (3 × *myc*, 5′-ATGGAGCAAAAGCTTATCAGTGAGGAAGACTTGAACGAGCAGAAGCTGATTTCCGAAGAGGATCTCAACGAGCAAAAGCTCATCTCGGAGGAAGACCTGCTC-3′) was inserted between the *BamH*I and *Sac*I sites in pGEM-WRKY45. A DNA linker containing an *Xba*I site was inserted between the *Sac*I and *Nsi*I sites in pGEM-WRKY45. Finally, a fragment encoding *WRKY45* CDS and 3 × *myc* sequence was excised with *Xho*I and *Xba*I, and inserted between *Xho*I and *Spe*I sites in a DEX-inducible gene expression vector, pTA7002 (Aoyama and Chua 1997). Rice plants were transformed by an *Agrobacterium* (strain EHA105)-mediated method (Toki et al. 2006) to generate transgenic GVG-WRKY45-myc plants.

### Quantitative RT-PCR

Total RNA was isolated from rice leaves using Trizol reagent (Invitrogen, www.invitrogen.jp/) and purified with an RNeasy mini kit (Qiagen, http://www.qiagen.com). The RNAs were treated with DNase (Takara, www.takara-bio.co.jp/) and reverse-transcribed into cDNA using Revertra Ace (Takara) and oligo(dT)_23_ primers (Sigma-Aldrich). qRT-PCR was performed with a Thermal Cycler Dice TP800 system (Takara) using a KAPA SYBR fast universal qPCR kit. Expression levels relative to the rice *ubiquitin 1* (*Rubq1,* Os06g0681400) gene, whose expression was not affected by *M. oryzae* infection both in NB and *WRKY45*-ox rice (Fig. S5), were quantified using the delta–delta Ct method. Primers used in this study are listed in Table S1.

### DP measurement

DPs were extracted as described elsewhere (Hasegawa et al. 2010). In brief, 0.05 g leaf material from each of the leaves from three seedlings was pooled and immediately frozen at −80 °C. The leaf material was ground into a powder with beads in a Retsch MM300 mixer mill (Qiagen), suspended in 40 volumes 70 % methanol, and boiled for 5 min. Supernatants were transferred to new tubes and the leaf residue was resuspended in 20 volumes 70 % methanol and boiled again for 5 min. This procedure was repeated once more. Then, all the supernatants were combined, dried in a vacuum centrifuge, and re-suspended in 0.5 ml 70 % methanol. DPs were quantified by HPLC–ESI–MS/MS as described elsewhere (Shimizu et al. 2008).

## Accession numbers

The sequences of genes used in this study can be found in the RAP database (http://rapdb.dna.affrc.go.jp/) under the following accession numbers: *Rubq1* (Os06g0681400), *CPS4* (Os04g0178300), *KSL4* (Os04g0179700), *CYP99A2* (Os04g0180400), *CYP99A3* (Os04g0178400), *MAS* (Os04g0179100), *CPS2* (Os02g0571100), *KSL7* (Os02g0570400), *KOL4* (Os06g0569500), *CYP71Z6* (Os02g0570500), *CYP71Z7* (Os02g0570700), and *KSL10* (Os12g0491800), *OsTGAP1* (Os04g0637000), *OsRR6* (Os04g0673300).

## Electronic supplementary material

Below is the link to the electronic supplementary material.

Supplementary material 1 (PDF 517 kb)

Supplementary material 2 (PDF 360 kb)

Supplementary material 3 (PDF 25 kb)
