# Supplementary material for: WRKY45-dependent priming of diterpenoid phytoalexin biosynthesis in rice and the role of cytokinin in triggering the reaction
Source: Plant Mol Biol. 2014 Jul 18;86(1):171–83. doi: 10.1007/s11103-014-0221-x (PMC4133022; doi:10.1007/s11103-014-0221-x)
Supplement: Supplementary file 1 — Supplementary material 1 (PDF 517 kb) [file 11103_2014_221_MOESM1_ESM.pdf]

WRKY45-dependent priming of diterpenoid phytoalexin biosynthesis in rice and the role of cytokinin in triggering the reaction
Plant Molecular Biology,
Aya Akagi, Setsuko Fukushima, Kazunori Okada, Chang-Jie Jiang, Riichiro Yoshida, Akira Nakayama, Masaki Shimono, Shoji Sugano, Hisakazu Yamane, Hiroshi Takatsuji,
National Institute of Agrobiological Sciences,
Corresponding author: Hiroshi Takatsuji E-mail: takatsuh@affrc.go.jp

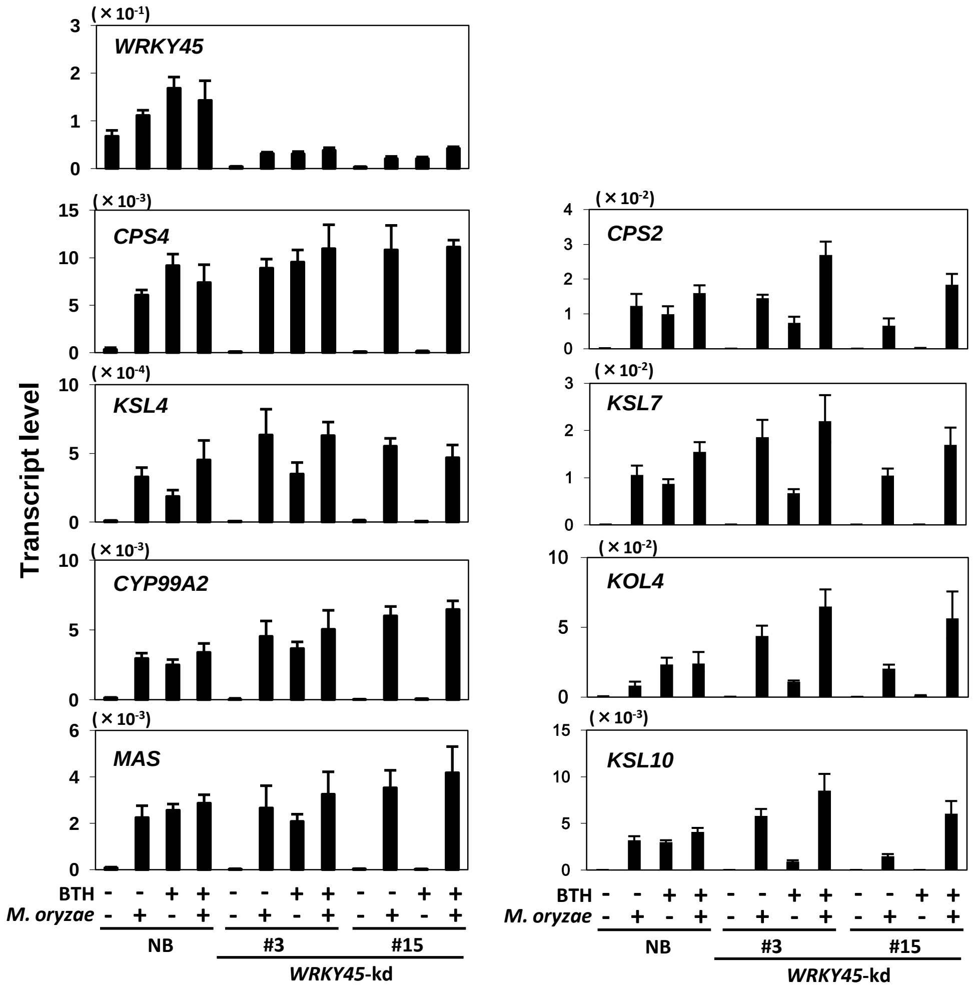

Figure S1. Momilactone biosynthetic genes were *WRKY45*-independently induced at 2 dpi. Nipponbare (NB) and *WRKY45*-kd rice lines at four-leaf stage were treated with BTH and/or spray-inoculated with *M. oryzae* (10^5 conidia/ml). Transcript levels of DP biosynthetic genes were determined according to the scheme in Figure 3A. Means of three determinations are shown with SD. DP biosynthetic genes were induced by BTH only. WRKY45 dependence of induction differed between the two *WRKY45*-kd lines, presumably due to different effectiveness of *WRKY45* knockdown.

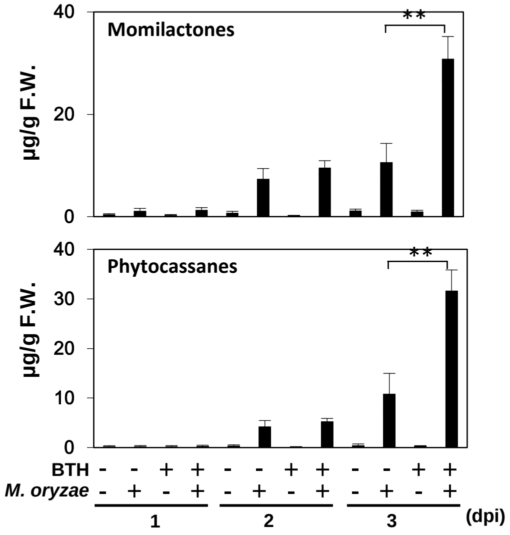

Figure S2. Accumulation of DPs in response to *M. oryzae* infection in BTH-treated rice plants. Nipponbare rice plants at four-leaf stage pretreated with 0.1 mM BTH at -1 d were spray-inoculated with *M. oryzae* (3.0×10^5 conidia/ml). Fourth leaves were harvested from four seedlings in each treatment and their DP content was analyzed. Means of six replicate samples are shown with SE. Momilactones and phytocassanes are sums of momilactone A and B, and phytocassane A – E, respectively. **: $p<0.01$ in student's *t*-test.

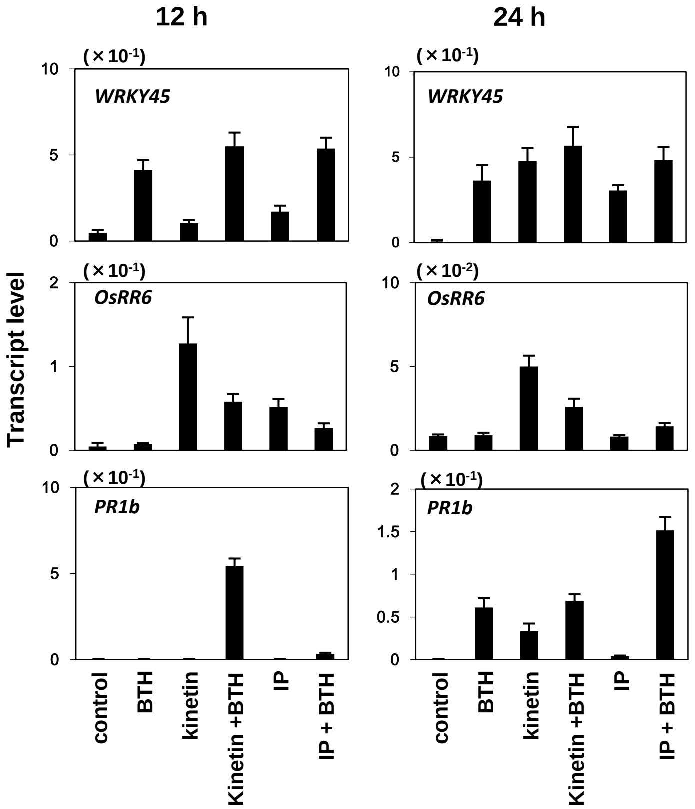

Figure S3. Effects of BTH and/or CKs on DP biosynthetic gene transcription in rice.

Roots of Nipponbare rice plants were treated with kinetin or IP (50 µM) and/or BTH (90 µM) for 12 and 24 h, and transcript levels of DP biosynthetic genes were determined by qRT-PCR. Same samples as those used in Figure 6A were used. Means of three determinations are shown with SD.

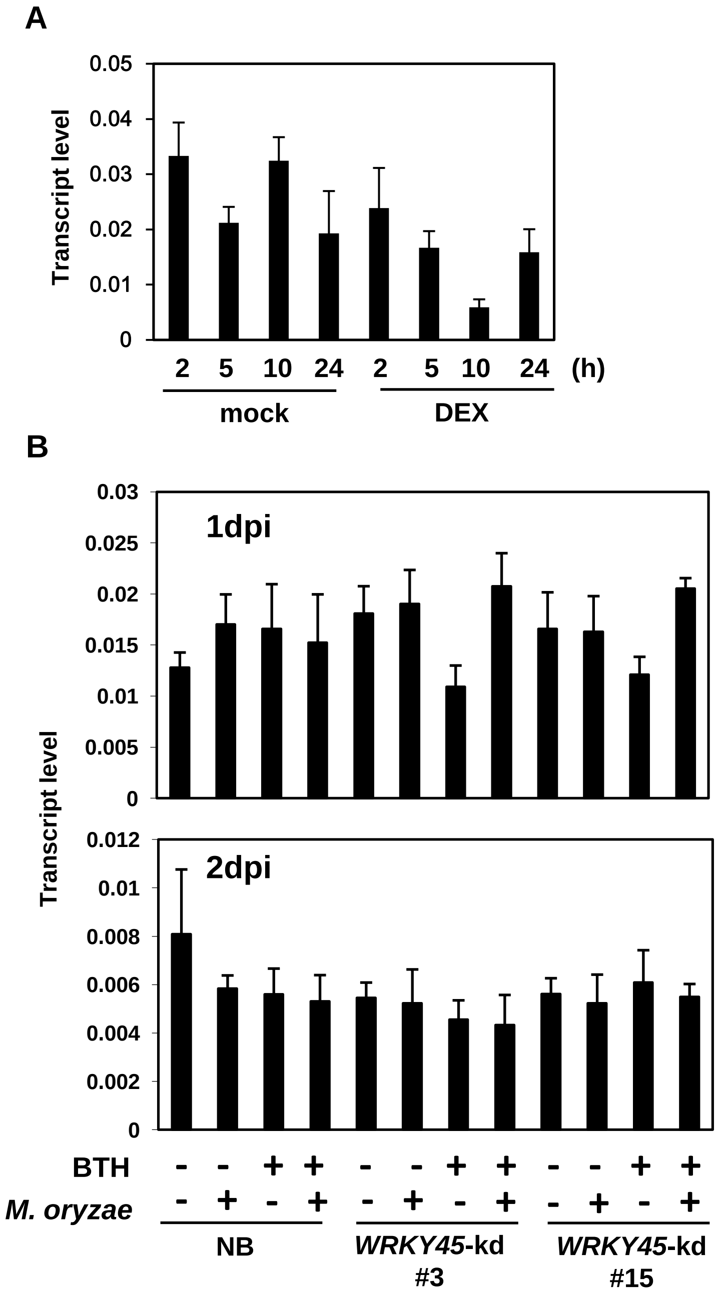

Figure S4. *TGAP1* transcription is not regulated by WRKY45.
Transcript levels of *TGAP1* in plants in which *WRKY45* expression was induced by DEX. Same samples as those used in Figure 3 was used.
Transcript levels of *TGAP1* gene in Nipponbare and *WRKY45*-kd rice plants with or without BTH treatment and *M. oryzae* infection.

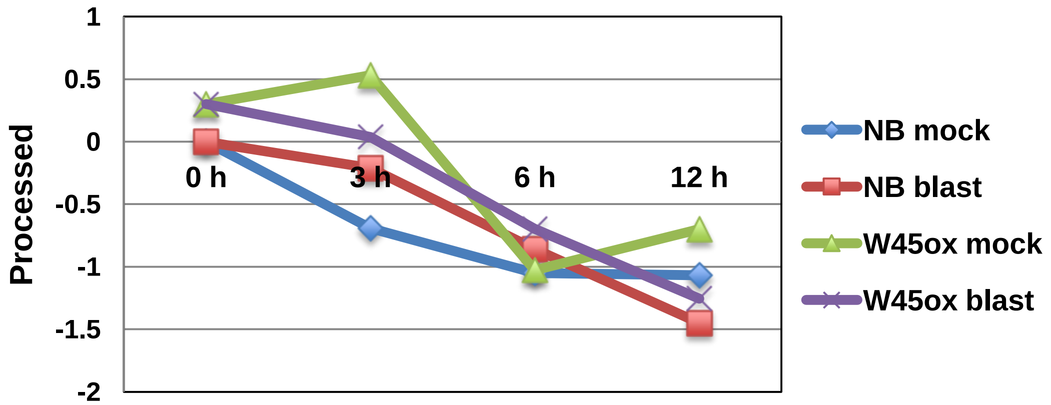

Figure S5. Expression of *ubiquitin 1* during *M. oryzae* infection in microarray. The *WRKY45*-ox and NB rice plants were spray-inoculated with *M. oryzae* (10^5 conidia/ml) or mock-treated, and then total RNAs extracted at 3, 6, and 12 hpi were analyzed by microarray in triplicates. After global normalization using mean values, the average processed data for *ubiquitin 1* (Os06g0681400) were plotted.
