# Supplementary material for: WRKY45-dependent priming of diterpenoid phytoalexin biosynthesis in rice and the role of cytokinin in triggering the reaction
Source: Plant Mol Biol. 2014 Jul 18;86(1):171–83. doi: 10.1007/s11103-014-0221-x (PMC4133022; doi:10.1007/s11103-014-0221-x)
Supplement: Supplementary file 2 — Supplementary material 2 (PDF 360 kb) [file 11103_2014_221_MOESM2_ESM.pdf]

WRKY45-dependent priming of diterpenoid phytoalexin biosynthesis in rice and the role of cytokinin in triggering the reaction

Plant Molecular Biology

Aya Akagi, Setsuko Fukushima, Kazunori Okada, Chang-Jie Jiang, Riichiro Yoshida, Akira Nakayama, Masaki Shimono, Shoji Sugano, Hisakazu Yamane, Hiroshi Takatsuji
National Institute of Agrobiological Sciences
Corresponding author: Hiroshi Takatsuji E-mail: takatsuh@affrc.go.jp

Table S1. List of upregulated genes in WRKY45-ox rice

Genes upregulated in WRKY45-ox rice (#21) relative to Nipponbare are listed with -fold changes, Q-values, BTH inducibility (Shimono et al., 2007), and annotations. Complete data set is available in the Gene Expression Omnibus repository, GSE48202.

measurement ID	Fold Change	Q-Value	BTH-inducible genes	Description
Os03g0115800|	3037.4	0.023187		Conserved hypothetical protein.
Os03g0115800|	2596.9	0.023187		Conserved hypothetical protein.
Os07g0122000|	245.3	0.023187	✓	Conserved hypothetical protein.
Os01g0692400|	172.1	0.024357		Conserved hypothetical protein.
Os05g0322900|	151.8	0.024211	✓	WRKY transcription factor 45.
Os08g0410900|	140.2	0.023187		Non-protein coding transcript, uncharacterized transcript.
Os05g0537700|	137.5	0.023187		Cyclin-like F-box domain containing protein.
Os02g0772100|	132.3	0.029566		Conserved hypothetical protein.
Os05g0322900|	131.2	0.028157	✓	WRKY transcription factor 45.
Os05g0322900|	120.1	0.027037	✓	WRKY transcription factor 45.
Os01g0916100|	117.6	0.032268	✓	Conserved hypothetical protein.
Os06g0265100|	112.5	0.023187		Hypothetical protein.
Os07g0511400|	112.0	0.026444		Hypothetical protein.
Os07g0133100|	109.4	0.023187		(No Hit)
Os06g0493100|	102.9	0.027037	✓	Hypothetical protein.
Os06g0493100|	98.7	0.023187	✓	Hypothetical protein.
Os11g0471200|	83.9	0.028009		Conserved hypothetical protein.
Os06g0210400|	78.6	0.029825		Legume lectin, beta domain containing protein.
Os09g0417800|	75.5	0.024357	✓	DNA-binding WRKY domain containing protein.
Os02g0277700|	72.9	0.023187	✓	Leucine-rich repeat, plant specific containing protein.
Os08g0176200|	71.9	0.02334		Protein kinase family protein.
Os07g0129300|	69.2	0.036494		Pathogenesis-related protein 1 precursor.
Os11g0691500|	66.8	0.025904		Protein kinase domain containing protein.
Os04g0202800|	64.7	0.032482	✓	Protein kinase domain containing protein.
Os09g0417600|	61.0	0.028052	✓	DNA-binding WRKY domain containing protein.

Os04g0369000|	59.6	0.027037	✓	Major sperm protein domain containing protein.
Os05g0296200|	53.5	0.028632		Conserved hypothetical protein.
Os12g0218800|	53.0	0.024211		Hypothetical protein.
Os04g0397800|	49.7	0.031493		Non-protein coding transcript, unclassifiable transcript.
Os12g0431100|	48.6	0.027136	✓	AAA ATPase, central region domain containing protein.
Os04g0154800|	47.8	0.027647	✓	Protein of unknown function DUF594 family protein.
Os10g0442300|	41.7	0.034878	✓	UDP-glucuronosyl/UDP-glucosyltransferase family protein.
Os09g0304800|	40.8	0.033167		Conserved hypothetical protein.
Os06g0328400|	39.5	0.038323		Legume lectin, beta domain containing protein.
Os09g0417600|	39.3	0.023187	✓	DNA-binding WRKY domain containing protein.
Os03g0776400|	39.1	0.027944	✓	Conserved hypothetical protein.
Os11g0433300|	37.6	0.026444		(No Hit)
Os08g0124100|	37.5	0.029322		Resistance protein candidate (Fragment).
Os09g0417600|	37.0	0.028052	✓	DNA-binding WRKY domain containing protein.
Os07g0471300|	34.8	0.023187		Argonaute 4 protein.
Os04g0399300|	34.4	0.023187	✓	NUDIX hydrolase domain containing protein.
Os04g0371700|	34.3	0.030557		Protein kinase domain containing protein.
Os06g0293500|	34.1	0.03032		Hypothetical protein.
Os01g0297200|	33.7	0.023187		AAA ATPase, central region domain containing protein.
Os04g0468600|	33.3	0.029161	✓	Conserved hypothetical protein.
Os02g0807800|	32.7	0.03131	✓	(No Hit)
Os08g0201700|	32.2	0.024202		Protein kinase domain containing protein.
Os04g0575900|	31.5	0.024357		(No Hit)
Os08g0316400|	31.3	0.024864		(No Hit)
Os08g0173600|	30.8	0.036968	✓	Hypothetical protein.
Os03g0446000|	30.8	0.024357		(No Hit)
Os01g0153000|	30.6	0.024357		Protein kinase domain containing protein.
Os04g0581000|	30.4	0.025768	✓	2OG-Fe(II) oxygenase domain containing protein.
Os07g0229600|	30.4	0.024357		(No Hit)
Os11g0572200|	30.3	0.024357		Hypothetical protein.
Os03g0812400|	30.3	0.032482		Calcium-binding EF-hand domain containing protein.
Os01g0916000|	29.8	0.033989		Conserved hypothetical protein.
Os06g0271400|	29.1	0.029399		Hypothetical protein.
Os04g0613000|	28.9	0.027647		Zinc transporter 1 precursor (ZRT/IRT-like protein 1).
Os08g0355600|	28.9	0.023187	✓	Hypothetical protein.
Os01g0291500|	28.9	0.026444		Transferase family protein.
Os04g0280500|	27.6	0.062408		Conserved hypothetical protein.

Os01g0915900|	27.1	0.026671		(No Hit)
Os04g0368000|	26.9	0.028217		(No Hit)
Os09g0471500|	26.6	0.032011	✓	Protein kinase domain containing protein.
Os07g0517600|	26.5	0.024571		AAA ATPase, central region domain containing protein.
Os01g0134700|	26.4	0.028939	✓	Conserved hypothetical protein.
Os05g0472600|	26.2	0.06048		Conserved hypothetical protein.
Os06g0579200|	25.9	0.034195		(No Hit)
osa-miR398a|C	25.4	0.05685		miRNA
osa-miR398a|C	25.0	0.057637		miRNA
Os02g0227700|	24.7	0.023187		Protein kinase domain containing protein.
Os11g0154500|	24.6	0.023187		No apical meristem (NAM) protein domain containing protein.
Os03g0115700|	24.4	0.024202		Short-chain dehydrogenase/reductase SDR family protein.
Os04g0370900|	24.3	0.030317	✓	Protein kinase domain containing protein.
Os08g0124000|	24.1	0.034795	✓	Resistance protein candidate (Fragment).
Os08g0201800|	24.1	0.027297		Copia protein.
Os02g0609900|	23.7	0.029161	✓	Leucine-rich repeat, typical subtype containing protein.
Os05g0493100|	23.3	0.024357		KI domain interacting kinase 1.
Os02g0111600|	22.8	0.04366	✓	EGF-like calcium-binding domain containing protein.
Os04g0267600|	22.5	0.023187		Cyclin-like F-box domain containing protein.
Os05g0223300|	22.3	0.02509		RNA-binding protein.
Os04g0581000|	22.3	0.024357	✓	2OG-Fe(II) oxygenase domain containing protein.
Os08g0124000|	22.2	0.024357	✓	Resistance protein candidate (Fragment).
Os08g0297800|	22.0	0.036051		Sulfotransferase family protein.
Os04g0613000|	21.9	0.028939		Zinc transporter 1 precursor (ZRT/IRT-like protein 1).
Os02g0645000|	21.8	0.024357		Zn-finger, RING domain containing protein.
Os11g0561100|	21.3	0.039661		(No Hit)
Os04g0468600|	21.1	0.028939	✓	Conserved hypothetical protein.
Os01g0117300|	21.0	0.029322		Protein kinase domain containing protein.
Os03g0328200|	21.0	0.039068		Conserved hypothetical protein.
Os07g0122100|	20.8	0.023187	✓	Conserved hypothetical protein.
Os08g0541300|	20.3	0.024357		Leucine-rich repeat, plant specific containing protein.
Os04g0468600|	20.1	0.029253	✓	Conserved hypothetical protein.
Os04g0371000|	20.0	0.024357		Conserved hypothetical protein.
Os10g0537800|	20.0	0.038912	✓	Peptidase A1, pepsin family protein.
Os04g0468600|	19.8	0.028939	✓	Conserved hypothetical protein.
Os03g0132900|	19.7	0.025319	✓	Chitinase (EC 3.2.1.14) (Fragment).
Os08g0213100|	19.7	0.039803		(No Hit)

Os01g0678000|	19.6	0.028939	✓	Conserved hypothetical protein.
Os09g0267800|	19.6	0.024211		(No Hit)
Os07g0526400|	19.6	0.035225	✓	Chalcone synthase (EC 2.3.1.74) (Naringenin-chalcone synthase).
Os01g0117200|	19.3	0.023187		ARK protein (Fragment).
Os01g0291500|	18.9	0.025768		Transferase family protein.
Os08g0198300|	18.7	0.028431		BTB/POZ domain containing protein.
Os04g0369100|	18.2	0.028426		Protein kinase domain containing protein.
Os06g0674100|	17.8	0.028939		Hypothetical protein.
Os07g0550600|	17.8	0.036827	✓	Transferase family protein.
Os11g0625900|	17.6	0.027037	✓	Protein kinase domain containing protein.
Os12g0249900|	17.6	0.024734		Protein kinase domain containing protein.
Os07g0532800|	17.5	0.024211	✓	Peptidase A1, pepsin family protein.
Os07g0447000|	17.5	0.02829		Protein of unknown function DUF594 family protein.
Os03g0132900|	16.9	0.024357	✓	Chitinase (EC 3.2.1.14) (Fragment).
Os01g0687400|	16.8	0.02334		Chitinase (EC 3.2.1.14).
Os12g0217800|	16.5	0.035746		Non-protein coding transcript, unclassifiable transcript.
Os10g0520100|	16.2	0.035225		Cyclin-like F-box domain containing protein.
Os10g0124300|	15.9	0.033763		Disease resistance protein family protein.
Os08g0351300|	15.9	0.032541	✓	Hypothetical protein.
Os11g0229300|	15.7	0.027647		NBS-LRR disease resistance protein homologue.
Os03g0106400|	15.6	0.030317		Branched-chain-amino-acid aminotransferase 5, chloroplast precursor (EC 2.6.1.42) (Atbcat-5).
Os11g0420000|	15.5	0.029281		Conserved hypothetical protein.
Os07g0460800|	15.4	0.023187		(No Hit)
Os10g0490100|	15.3	0.060083	✓	Barwin-related endoglucanase domain containing protein.
Os04g0563000|	15.3	0.027944		Conserved hypothetical protein.
Os01g0687400|	15.2	0.023714		Chitinase (EC 3.2.1.14).
Os05g0166300|	15.1	0.034874		Protein kinase domain containing protein.
Os04g0561500|	15.1	0.035596		Prolyl endopeptidase (EC 3.4.21.26) (Post-proline cleaving enzyme) (PE).
Os07g0689600|	15.1	0.030557		Nicotianamine synthase 9 (EC 2.5.1.43) (S-adenosyl-L-methionine:S- adenosyl-L-methionine:S-adenosyl-methionine 3-amino-3-carboxypropyltransferase 9).
Os12g0255200|	15.0	0.035225		Ca(2+)-dependent nuclease.
Os07g0685500|	15.0	0.023187	✓	Alpha/beta hydrolase family protein.
Os04g0561500|	14.9	0.042696		Prolyl endopeptidase (EC 3.4.21.26) (Post-proline cleaving enzyme) (PE).
Os04g0611400|	14.8	0.024734	✓	Vacuolar sorting receptor homolog.
Os09g0417600|	14.8	0.023187	✓	DNA-binding WRKY domain containing protein.

Os05g0492600|	14.8	0.030652		NBS-LRR type resistance protein (Fragment).
Os07g0132000|	14.8	0.025768		(No Hit)
Os11g0669100|	14.7	0.030557	✓	Hypothetical protein.
Os10g0569600|	14.6	0.034884		RIR1b protein precursor.
Os01g0824800|	14.6	0.023187	✓	Conserved hypothetical protein.
Os04g0611400|	14.5	0.024864	✓	Vacuolar sorting receptor homolog.
Os10g0531400|	14.4	0.024734	✓	Glutathione S-transferase GST 30 (EC 2.5.1.18).
Os04g0227000|	14.3	0.023187		Protein kinase domain containing protein.
Os03g0132900|	14.2	0.028269	✓	Chitinase (EC 3.2.1.14) (Fragment).
Os12g0556500|	14.2	0.02576	✓	Hypothetical protein.
Os04g0149400|	14.1	0.024357	✓	Hypothetical protein.
Os10g0538200|	14.1	0.040322		Peptidase A1, pepsin family protein.
Os07g0689600|	14.0	0.030434		Nicotianamine synthase 9 (EC 2.5.1.43) (S-adenosyl-L-methionine:S- adenosyl-L-methionine:S-adenosyl-methionine 3-amino-3-carboxypropyltransferase 9).
Os03g0657400|	13.9	0.023187	✓	WRKY transcription factor 60.
Os10g0503300|	13.9	0.03799	✓	Benzoyl coenzyme A: benzyl alcohol benzoyl transferase.
Os10g0531400|	13.9	0.025056	✓	Glutathione S-transferase GST 30 (EC 2.5.1.18).
Os05g0324700|	13.8	0.027647	✓	Hypothetical protein.
Os02g0582800|	13.8	0.025768	✓	Conserved hypothetical protein.
Os12g0629700|	13.8	0.030557	✓	Thaumatin-like protein precursor.
Os12g0130200|	13.7	0.036494		Ser/Thr protein kinase (Fragment).
Os06g0688800|	13.6	0.039488		(No Hit)
Os12g0628600|	13.6	0.061785	✓	Thaumatin-like protein precursor.
Os12g0141000|	13.5	0.035049		Hypothetical protein.
Os07g0251200|	13.4	0.034874	✓	Harpin-induced 1 domain containing protein.
Os06g0673700|	13.4	0.027136		Hypothetical protein.
Os08g0391300|	13.4	0.027136		Non-protein coding transcript, putative npRNA.
Os08g0124900|	13.4	0.025319	✓	Protein kinase family protein.
Os01g0628900|	13.3	0.0379	✓	Cytochrome P450 family protein.
Os02g0738300|	13.2	0.038644		Conserved hypothetical protein.
Os11g0144900|	13.2	0.029729		Hypothetical protein.
Os10g0132500|	13.1	0.029566		Disease resistance protein family protein.
Os11g0227700|	12.9	0.02912		RPR1.
Os06g0591200|	12.9	0.024357		Conserved hypothetical protein.
Os06g0591200|	12.9	0.025296		Conserved hypothetical protein.
Os04g0368000|	12.8	0.033676		(No Hit)
Os04g0397800|	12.8	0.033395		Non-protein coding transcript, unclassifiable transcript.
osa-miR398a|C	12.7	0.071203		miRNA

Os08g0404500|	12.6	0.024357	✓	C1-like domain containing protein.
Os08g0466200|	12.5	0.030882		K+ potassium transporter family protein.
Os03g0335200|	12.5	0.028939	✓	WRKY DNA binding protein.
Os10g0328600|	12.4	0.078773		Conserved hypothetical protein.
Os01g0690200|	12.4	0.036191		Conserved hypothetical protein.
Os04g0468600|	12.3	0.033167	✓	Conserved hypothetical protein.
Os01g0690800|	12.3	0.028541	✓	Protein kinase domain containing protein.
Os11g0686500|	12.2	0.027944		Disease resistance protein family protein.
Os08g0124500|	12.1	0.028939		Resistance protein candidate (Fragment).
Os08g0404500|	12.1	0.028939	✓	C1-like domain containing protein.
Os11g0676500|	12.1	0.030378		NBS-LRR type resistance protein (Fragment).
Os09g0565300|	12.1	0.034874		(No Hit)
Os10g0569800|	11.9	0.024357		Hypothetical protein.
Os12g0255200|	11.9	0.0379		Ca(2+)-dependent nuclease.
Os03g0664800|	11.8	0.039528	✓	GCN5-related N-acetyltransferase domain containing protein.
Os06g0676700|	11.7	0.044742		High pI alpha-glucosidase.
Os11g0567800|	11.6	0.028939	✓	HcrVf2 protein.
Os01g0111700|	11.6	0.032782	✓	Conserved hypothetical protein.
Os08g0256700|	11.5	0.034324		Conserved hypothetical protein.
Os08g0124500|	11.5	0.028694		Resistance protein candidate (Fragment).
Os10g0433100|	11.4	0.042254		(No Hit)
Os04g0179700|	11.3	0.028157	✓	Terpene synthase.
Os06g0125800|	11.3	0.033595		Zn-finger, RING domain containing protein.
Os08g0466200|	11.3	0.027481		K+ potassium transporter family protein.
Os04g0365100|	11.1	0.029039		Wall-associated kinase-like protein.
Os04g0301500|	11.1	0.067522		Basic helix-loop-helix dimerisation region bHLH domain containing protein.
Os01g0186600|	11.0	0.037081		Conserved hypothetical protein.
Os01g0564300|	11.0	0.023187		Peptidylprolyl isomerase, FKBP-type domain containing protein.
Os07g0686800|	10.8	0.024357		Serine/threonine protein kinase-like.
Os08g0466200|	10.6	0.030539		K+ potassium transporter family protein.
Os06g0557700|	10.6	0.03017		Protein kinase domain containing protein.
Os09g0471400|	10.6	0.023187		Protein kinase domain containing protein.
Os06g0125800|	10.6	0.034325		Zn-finger, RING domain containing protein.
Os07g0586800|	10.6	0.039127		Conserved hypothetical protein.
Os09g0365900|	10.5	0.024357		L-ascorbate oxidase precursor (EC 1.10.3.3) (Ascorbase) (ASO).
Os07g0442800|	10.5	0.032734		Conserved hypothetical protein.
Os01g0690800|	10.4	0.023187		Protein kinase domain containing protein.
Os11g0667600|	10.4	0.065111	✓	Protein kinase family protein.

Os01g0160900|	10.4	0.035821		Leucine-rich repeat, plant specific containing protein.
Os01g0804200|	10.3	0.024864		Cytochrome P450 family protein.
Os04g0288500|	10.3	0.050311		Protein kinase domain containing protein.
Os03g0737300|	10.2	0.027481		Magnesium-dependent phosphatase-1 family protein.
Os06g0676700|	10.2	0.045937		High pI alpha-glucosidase.
Os03g0412400|	10.2	0.024066	✓	Conserved hypothetical protein.
Os01g0891500|	10.1	0.02847		GroES-like domain containing protein.
Os11g0229500|	10.1	0.03664	✓	Disease resistance protein family protein.
Os01g0724700|	10.1	0.024357		(No Hit)
Os01g0322700|	10.1	0.031314		Non-protein coding transcript, unclassifiable transcript.
Os06g0579000|	10.0	0.024357	✓	Hypothetical protein.
Os04g0179700|	10.0	0.061585	✓	Terpene synthase.
Os01g0115600|	9.9	0.029629	✓	LRK14.
Os04g0151600|	9.9	0.024357		Hypothetical protein.
Os08g0127100|	9.8	0.028939	✓	Lysine and histidine specific transporter.
Os11g0229500|	9.7	0.035872	✓	Disease resistance protein family protein.
Os10g0473900|	9.7	0.029281		Disease resistance protein family protein.
Os09g0570100|	9.7	0.025768	✓	Protein kinase domain containing protein.
Os04g0480500|	9.6	0.029504		Leucine-rich repeat, plant specific containing protein.
Os11g0132900|	9.6	0.042716		Protein kinase family protein.
Os12g0222900|	9.6	0.03131		Leucine-rich repeat, typical subtype containing protein.
Os01g0117500|	9.6	0.039488	✓	LRK14.
Os04g0128700|	9.6	0.045944	✓	Conserved hypothetical protein.
Os03g0724700|	9.5	0.026444		Maf-like protein family protein.
Os04g0366000|	9.5	0.023187		EGF-like calcium-binding domain containing protein.
Os09g0479300|	9.5	0.024357		Conserved hypothetical protein.
Os12g0593700|	9.5	0.024357		(No Hit)
Os06g0344100|	9.5	0.028939		(No Hit)
Os11g0229500|	9.4	0.036945	✓	Disease resistance protein family protein.
Os09g0356800|	9.4	0.028939		Protein kinase domain containing protein.
Os06g0142500|	9.3	0.028939		Calycin family protein.
Os01g0879200|	9.3	0.058518		Conserved hypothetical protein.
Os01g0588400|	9.3	0.02829	✓	Band 7 protein family protein.
Os07g0493800|	9.2	0.032734		Protein kinase domain containing protein.
Os07g0670200|	9.2	0.043271		C2 domain containing protein.
Os10g0569400|	9.2	0.026909		RIR1a protein precursor.
Os04g0108900|	9.2	0.024734		(No Hit)

Os02g0281900|	9.2	0.030805	✓	Disease resistance protein family protein.
Os11g0282700|	9.2	0.032268		Homeodomain-like containing protein.
Os02g0212400|	9.1	0.036727		Conserved hypothetical protein.
Os10g0569400|	9.1	0.024357		RIR1a protein precursor.
Os11g0558900|	9.1	0.023714		Leucine-rich repeat, plant specific containing protein.
Os02g0738200|	9.1	0.04109		U box domain containing protein.
Os06g0261300|	9.1	0.024357		Hypothetical protein.
Os01g0585200|	9.1	0.050429	✓	Conserved hypothetical protein.
Os07g0220200|	9.1	0.041384	✓	(No Hit)
Os04g0616700|	9.0	0.028939	✓	BRASSINOSTEROID INSENSITIVE 1-associated receptor kinase 1 precursor (EC 2.7.1.37) (BRI1-associated receptor kinase 1) (Somatic embryogenesis receptor-like kinase 3).
Os06g0200800|	9.0	0.030987		Conserved hypothetical protein.
Os04g0288100|	9.0	0.029825		Adenosine diphosphate glucose pyrophosphatase precursor.
Os12g0218900|	9.0	0.042125		(No Hit)
Os05g0533400|	8.9	0.029161		NB-ARC domain containing protein.
Os10g0184200|	8.8	0.023187		Protein of unknown function DUF594 family protein.
Os08g0356700|	8.8	0.038291	✓	Plant protein of unknown function family protein.
Os12g0229700|	8.8	0.04032		(No Hit)
Os11g0666200|	8.8	0.023187		Protein kinase domain containing protein.
Os11g0479000|	8.8	0.04247	✓	Hypothetical protein.
Os10g0386300|	8.8	0.028193		Hypothetical protein.
Os04g0141500|	8.7	0.040768		(No Hit)
Os10g0334100|	8.7	0.039803		(No Hit)
Os11g0192600|	8.6	0.037948		(No Hit)
Os05g0368000|	8.6	0.039487	✓	Conserved hypothetical protein.
Os02g0306400;	8.6	0.043009		OsNAAT1 mRNA for nicotianamine aminotransferase
Os09g0570100|	8.6	0.024211	✓	Protein kinase domain containing protein.
Os08g0348800|	8.5	0.038674		(No Hit)
Os06g0271100|	8.5	0.038674		(No Hit)
Os06g0618900|	8.4	0.040342		(No Hit)
Os07g0215600|	8.4	0.040322		(No Hit)
Os04g0280700|	8.4	0.023187		(No Hit)
Os02g0577500|	8.4	0.023187		(No Hit)
Os06g0697600|	8.4	0.023187		AAA ATPase, central region domain containing protein.
Os03g0204500|	8.4	0.034294		(No Hit)
Os04g0339800|	8.4	0.024211		Protein kinase family protein.

Os11g0225000|	8.4	0.034918		Serine/threonine protein kinase domain containing protein.
Os05g0303300|	8.3	0.041068		(No Hit)
Os11g0230900|	8.3	0.040932		(No Hit)
Os12g0221700|	8.3	0.036653	✓	(No Hit)
Os01g0554900|	8.3	0.039753		(No Hit)
Os03g0724700|	8.2	0.026671		Maf-like protein family protein.
Os03g0841400|	8.2	0.044152		(No Hit)
Os04g0451600|	8.2	0.041863		(No Hit)
Os01g0668600|	8.2	0.024357	✓	Resistance protein candidate (Fragment).
Os02g0205500|	8.2	0.040752	✓	Naringenin-chalcone synthase family protein.
Os04g0219900|	8.1	0.04213		(No Hit)
Os08g0127100|	8.1	0.03562	✓	Lysine and histidine specific transporter.
Os04g0268000|	8.1	0.04109		(No Hit)
Os11g0550500|	8.1	0.024202		LZ-NBS-LRR class RGA.
Os01g0601700|	8.1	0.032268		Leucine-rich repeat, typical subtype containing protein.
Os02g0671500|	8.1	0.042401		(No Hit)
Os07g0536500|	8.1	0.04213		(No Hit)
Os12g0511900|	8.1	0.030882		(No Hit)
Os03g0772600|	8.1	0.024357		Protein kinase family protein.
Os06g0726300|	8.1	0.036164	✓	No apical meristem (NAM) protein family protein.
Os07g0163400|	8.0	0.023187		(No Hit)
Os12g0463300|	8.0	0.023187		(No Hit)
Os06g0709500|	8.0	0.040266		(No Hit)
Os02g0481400|	8.0	0.023187		(No Hit)
Os04g0195800|	8.0	0.040266		(No Hit)
Os07g0611100|	8.0	0.041284		(No Hit)
Os08g0327900|	8.0	0.042737		(No Hit)
Os02g0763000|	8.0	0.023187		Uncharacterized Cys-rich domain containing protein.
Os11g0235000|	7.9	0.041781		(No Hit)
Os09g0471800|	7.9	0.024202		EGF-like calcium-binding domain containing protein.
Os10g0131700|	7.9	0.028939		NB-ARC domain containing protein.
Os08g0124600|	7.9	0.023187		(No Hit)
Os04g0518400|	7.9	0.03441	✓	Phenylalanine ammonia-lyase 2 (EC 4.3.1.5).
Os08g0149600|	7.9	0.041256		(No Hit)
Os02g0209300|	7.9	0.037948	✓	Non-protein coding transcript, unclassifiable transcript.
Os08g0543100|	7.8	0.024357		Disease resistance protein family protein.
Os08g0562300|	7.8	0.051442		Zn-finger, C2H2 type domain containing protein.
Os02g0281200|	7.8	0.033374		Disease resistance protein family protein.

Os01g0307300|	7.8	0.040085		(No Hit)
Os04g0333700|	7.8	0.040322		(No Hit)
Os01g0176000|	7.8	0.023292	✓	UDP-glucuronosyl/UDP-glucosyltransferase family protein.
Os10g0569500|	7.8	0.028939		Conserved hypothetical protein.
Os10g0441900|	7.8	0.032541		Lectin-like receptor kinase 7;2.
Os05g0410200|	7.8	0.040033		Esterase/lipase/thioesterase domain containing protein.
Os07g0537900|	7.8	0.028267		SRK3 gene.
Os06g0688700|	7.8	0.044701		(No Hit)
Os12g0218500|	7.8	0.032734		(No Hit)
Os04g0108900|	7.8	0.041109		(No Hit)
Os07g0488800|	7.7	0.040033		(No Hit)
Os06g0163000|	7.7	0.030317		Heat shock protein STI (Stress inducible protein) (GmSTI).
Os12g0629300|	7.7	0.039135	✓	Thaumatin-like protein precursor.
Os05g0550700|	7.7	0.02334		Protein kinase domain containing protein.
Os04g0213400|	7.7	0.039918		(No Hit)
Os12g0265900|	7.7	0.028217	✓	(No Hit)
Os06g0625300|	7.6	0.031633		Peptidoglycan-binding LysM domain containing protein.
Os06g0278700|	7.6	0.041736		(No Hit)
Os08g0356500|	7.6	0.023187	✓	Plant protein of unknown function family protein.
Os01g0798800|	7.6	0.024357		Protein of unknown function DUF594 family protein.
Os01g0322700|	7.6	0.034874		Non-protein coding transcript, unclassifiable transcript.
Os08g0289400|	7.5	0.023187		Zn-finger, CCHC type domain containing protein.
Os10g0375000|	7.5	0.029457		(No Hit)
Os04g0167800|	7.5	0.024357	✓	Chalcone reductase homologue (Fragment).
Os04g0648200|	7.5	0.036414		Leucine-rich repeat, plant specific containing protein.
Os12g0473100|	7.5	0.041256		(No Hit)
Os02g0554900|	7.4	0.032076		Protein disulfide-isomerase precursor (EC 5.3.4.1) (PDI).
Os04g0127500|	7.4	0.033395	✓	EGF-like calcium-binding domain containing protein.
Os02g0659900|	7.4	0.038154		(No Hit)
Os04g0227500|	7.4	0.033523	✓	DSBA oxidoreductase family protein.
Os08g0124700|	7.3	0.03131		Resistance protein candidate (Fragment).
Os11g0454100|	7.3	0.024357		(No Hit)
Os10g0419400|	7.3	0.024357		Submergence induced protein 2.
Os01g0844300|	7.2	0.032542		Peptidylprolyl isomerase.
Os03g0270500|	7.2	0.025768	✓	Protein of unknown function DUF668 family protein.
Os03g0108100|	7.2	0.044169		(No Hit)

Os07g0538200|	7.2	0.023557	✓	Protein kinase domain containing protein.
Os05g0507200|	7.2	0.037165		60S ribosomal protein L38.
Os03g0327700|	7.2	0.02847		Hypothetical protein.
Os03g0854900|	7.2	0.028939		(No Hit)
Os11g0668300|	7.2	0.042401		Hypothetical protein.
Os03g0150600|	7.2	0.035049	✓	High affinity phosphate transporter 2 (Phosphate transporter).
Os02g0631100|	7.1	0.030887		Conserved hypothetical protein.
Os09g0355400|	7.1	0.073177		Protein kinase domain containing protein.
Os04g0390800|	7.1	0.043404		Short-chain dehydrogenase/reductase SDR family protein.
Os05g0570700|	7.1	0.024357		(No Hit)
Os11g0227200|	7.1	0.024211	✓	RPR1.
Os06g0671600|	7.1	0.043963		Non-protein coding transcript, putative npRNA.
Os09g0355400|	7.0	0.040752	✓	Protein kinase domain containing protein.
Os09g0129500|	7.0	0.023187		Conserved hypothetical protein.
Os02g0270900|	7.0	0.023187		Peptidase S8 and S53, subtilisin, kexin, sedolisin domain containing protein.
Os10g0570200|	7.0	0.029566	✓	RIR1b protein precursor.
Os01g0132100|	7.0	0.034878		Leucine-rich repeat, plant specific containing protein.
Os09g0304400|	7.0	0.035965		Conserved hypothetical protein.
Os04g0227500|	7.0	0.031438	✓	DSBA oxidoreductase family protein.
Os12g0527700|	7.0	0.023187		Protein kinase domain containing protein.
Os02g0554900|	6.9	0.030557		Protein disulfide-isomerase precursor (EC 5.3.4.1) (PDI).
Os11g0502700|	6.9	0.028052	✓	Hypothetical protein.
Os11g0672200|	6.9	0.029399		Protein kinase-like domain containing protein.
Os02g0306400:	6.9	0.056389		OsNAAT1 mRNA for nicotianamine aminotransferase
Os06g0291800|	6.9	0.029566		(No Hit)
Os11g0156600|	6.9	0.027647		(No Hit)
Os10g0328700|	6.8	0.069523		TPR-like domain containing protein.
Os01g0347200|	6.8	0.037742		Hypothetical protein.
Os10g0375000|	6.8	0.024357		(No Hit)
Os08g0462900|	6.8	0.041346		Protein of unknown function DUF716 family protein.
Os01g0115700|	6.8	0.03032	✓	Protein kinase family protein.
Os12g0170800|	6.7	0.039488	✓	24 kDa protein SC24 (24 kDa seed coat protein).
Os10g0142600|	6.7	0.024357		Protein kinase domain containing protein.
Os08g0198900|	6.7	0.04032		DNA-binding WRKY domain containing protein.
Os01g0114900|	6.7	0.029766		Protein kinase domain containing protein.
Os12g0156100|	6.7	0.036774		NAC-domain containing protein 90 (ANAC090).
Os06g0589300|	6.7	0.032326		Viral coat and capsid protein family protein.

Os09g0541100|	6.6	0.029457	✓	Plastocyanin-like domain containing protein.
Os06g0726100|	6.5	0.038006	✓	Endochitinase precursor (EC 3.2.1.14).
Os07g0538200|	6.5	0.030557	✓	Protein kinase domain containing protein.
Os04g0582700|	6.5	0.030434		Conserved hypothetical protein.
Os04g0303500|	6.4	0.038674		Protein kinase domain containing protein.
Os08g0346900|	6.4	0.052483		(No Hit)
Os12g0555000|	6.4	0.036382		Bet v I allergen family protein.
Os09g0517100|	6.4	0.029434	✓	Disease resistance protein family protein.
Os09g0371200|	6.4	0.027944		Major facilitator superfamily protein.
Os10g0534700|	6.4	0.032204	✓	Protein of unknown function DUF604 family protein.
Os05g0256500|	6.4	0.028939		Protein kinase domain containing protein.
Os04g0227500|	6.4	0.032011	✓	DSBA oxidoreductase family protein.
Os10g0483400|	6.4	0.030858		Protein kinase domain containing protein.
Os03g0830400|	6.4	0.027944		PGPS/D12.
Os10g0552400|	6.3	0.029399		U box domain containing protein.
Os03g0150600|	6.3	0.050116	✓	High affinity phosphate transporter 2 (Phosphate transporter).
Os08g0540900|	6.3	0.024357	✓	Conserved hypothetical protein.
Os07g0232800|	6.3	0.034878	✓	Zinc transporter ZIP family protein.
Os01g0714600|	6.3	0.040033	✓	Conserved hypothetical protein.
Os06g0210000|	6.3	0.056147	✓	Protein of unknown function DUF6 domain containing protein.
Os10g0180800|	6.3	0.042771	✓	EGF-like calcium-binding domain containing protein.
Os05g0485300|	6.3	0.029566		Eukaryotic protein of unknown function DUF887 family protein.
Os02g0665800|	6.3	0.032599		Protein kinase domain containing protein.
Os08g0483900|	6.3	0.034874	✓	Basic helix-loop-helix dimerisation region bHLH domain containing protein.
Os07g0106200|	6.3	0.028939	✓	Monosaccharide transporter 3.
Os02g0579600|	6.2	0.024734		Transcription factor MADS27.
Os04g0175600|	6.2	0.092048		Caffeic acid O-methyltransferase (EC 2.1.1.6).
Os12g0127200|	6.2	0.03472		Harpin-induced 1 domain containing protein.
Os01g0660200|	6.2	0.032542	✓	Acidic class III chitinase OsChib3a precursor (Chitinase) (EC 3.2.1.14).
Os01g0575200|	6.2	0.025904	✓	Rb (Fragment).
Os01g0660200|	6.2	0.032482		Acidic class III chitinase OsChib3a precursor (Chitinase) (EC 3.2.1.14).
Os11g0592100|	6.2	0.023187		Barwin.
Os07g0232800|	6.2	0.033205	✓	Zinc transporter ZIP family protein.
Os03g0150800|	6.2	0.028217	✓	High affinity phosphate transporter 2 (Phosphate transporter).
Os08g0457000|	6.2	0.04406		Conserved hypothetical protein.
Os03g0838400|	6.2	0.0379	✓	Ammonium transporter.

Os03g0140900|	6.2	0.029457		Hypothetical protein.
Os03g0150600|	6.2	0.046098	✓	High affinity phosphate transporter 2 (Phosphate transporter).
osa-miR419|Os	6.2	0.040562		miRNA
Os11g0109500|	6.2	0.027401		Staphylococcus nuclease (SNase-like) domain containing protein.
Os12g0170800|	6.1	0.037165	✓	24 kDa protein SC24 (24 kDa seed coat protein).
Os01g0115900|	6.1	0.039127		Protein kinase domain containing protein.
Os04g0178300|	6.1	0.024357	✓	Copalyl diphosphate synthetase (Fragment).
Os01g0660200|	6.1	0.03108	✓	Acidic class III chitinase OsChib3a precursor (Chitinase) (EC 3.2.1.14).
Os11g0600600|	6.1	0.02576	✓	Non-protein coding transcript, uncharacterized transcript.
Os11g0649700|	6.1	0.032011	✓	Protein of unknown function DUF588 family protein.
Os08g0127800|	6.0	0.092712		Conserved hypothetical protein.
Os01g0824600|	6.0	0.023187		Protein kinase domain containing protein.
Os12g0130500|	6.0	0.024202		(No Hit)
Os03g0838400|	6.0	0.039068	✓	Ammonium transporter.
Os11g0592100|	6.0	0.02847		Barwin.
Os05g0410200|	5.9	0.043128		Esterase/lipase/thioesterase domain containing protein.
Os10g0445900|	5.9	0.030434	✓	Hypothetical protein.
Os01g0660200|	5.9	0.033095	✓	Acidic class III chitinase OsChib3a precursor (Chitinase) (EC 3.2.1.14).
Os01g0613800|	5.9	0.045602		Peptidase C1A, papain family protein.
Os08g0170700|	5.9	0.037913		Disease resistance protein family protein.
Os03g0603600|	5.9	0.024357		Glycerophosphoryl diester phosphodiesterase family protein.
Os09g0125900|	5.9	0.098959		ATP-binding cassette, sub-family G, member 1 (White protein homolog) (ATP-binding cassette transporter 8). Splice isoform 3.
Os03g0415200|	5.9	0.025768		MAP3K protein kinase-like protein.
Os04g0340100|	5.9	0.028939	✓	Protein kinase-like domain containing protein.
Os01g0584900|	5.9	0.028939	✓	WRKY transcription factor 67.
Os05g0410200|	5.9	0.043375		Esterase/lipase/thioesterase domain containing protein.
Os04g0229100|	5.8	0.024357	✓	Cinnamyl-alcohol dehydrogenase CAD1 (Putative alcohol dehydrogenase) (EC 1.1.1.195).
Os01g0940700|	5.8	0.07471		Beta-1,3-glucanase (Fragment).
Os04g0303300|	5.8	0.029161		(No Hit)
Os03g0421500|	5.8	0.031192		Conserved hypothetical protein.
Os10g0135100|	5.8	0.03385		NB-ARC domain containing protein.
Os10g0490800|	5.8	0.03472		Plant Basic Secretory Protein family protein.
Os12g0218300|	5.8	0.045371		Hypothetical protein.

Os01g0149700|	5.8	0.03938		Protein kinase domain containing protein.
Os07g0489000|	5.7	0.039488	✓	(No Hit)
Os11g0565000|	5.7	0.037081		Leucine-rich repeat, plant specific containing protein.
Os04g0303100|	5.7	0.032457		Protein kinase domain containing protein.
Os01g0347300|	5.7	0.030882		(No Hit)
Os12g0272800|	5.7	0.025768		(No Hit)
Os07g0511100|	5.7	0.056644		Glycine-rich protein precursor.
Os06g0222100|	5.7	0.038154		Trehalose-phosphatase family protein.
Os04g0616700|	5.7	0.041283	✓	BRASSINOSTEROID INSENSITIVE 1-associated receptor kinase 1 precursor (EC 2.7.1.37) (BRI1-associated receptor kinase 1) (Somatic embryogenesis receptor-like kinase 3).
Os04g0654400|	5.7	0.024357		Hypothetical protein.
Os03g0584400|	5.7	0.035872		AAA-type ATPase-like protein.
Os02g0738100|	5.7	0.024357	✓	(No Hit)
Os03g0133000|	5.7	0.030557	✓	No apical meristem (NAM) protein domain containing protein.
Os01g0114100|	5.7	0.027136		Protein kinase family protein.
Os07g0268800|	5.7	0.029826		Protein of unknown function DUF594 family protein.
Os07g0273600|	5.7	0.030913		Hypothetical protein.
Os09g0498500|	5.7	0.037213		NAD-binding site containing protein.
Os01g0946300|	5.7	0.023187		Lecithin:cholesterol acyltransferase family protein.
Os11g0692300|	5.7	0.024357		Bacterial blight resistance protein.
Os01g0115800|	5.6	0.038674		Protein kinase domain containing protein.
Os12g0547600|	5.6	0.036618	✓	Hypothetical protein.
Os10g0180800|	5.6	0.024066	✓	EGF-like calcium-binding domain containing protein.
Os04g0647900|	5.6	0.030216		Leucine-rich repeat, typical subtype containing protein.
Os01g0940700|	5.6	0.069387		Beta-1,3-glucanase (Fragment).
Os12g0555000|	5.6	0.030791	✓	Bet v I allergen family protein.
Os01g0940700|	5.6	0.072022		Beta-1,3-glucanase (Fragment).
Os01g0944500|	5.6	0.074814		Glycoside hydrolase, family 17 protein.
Os11g0514400|	5.6	0.028217	✓	BRASSINOSTEROID INSENSITIVE 1-associated receptor kinase 1 precursor (EC 2.7.1.37) (BRI1-associated receptor kinase 1) (Somatic embryogenesis receptor-like kinase 3).
Os01g0786700|	5.6	0.032011	✓	Conserved hypothetical protein.
Os10g0491000|	5.6	0.03562		Plant Basic Secretory Protein family protein.
Os07g0106200|	5.5	0.036774	✓	Monosaccharide transporter 3.
Os04g0469000|	5.5	0.036171	✓	Heavy metal transport/detoxification protein domain containing protein.
Os04g0229100|	5.5	0.025904	✓	Cinnamyl-alcohol dehydrogenase CAD1 (Putative alcohol dehydrogenase) (EC 1.1.1.195).

Os11g0226900|	5.5	0.033904	✓	(No Hit)
Os06g0726100|	5.5	0.028968	✓	Endochitinase precursor (EC 3.2.1.14).
Os01g0864200|	5.5	0.034437	✓	Conserved hypothetical protein.
Os08g0125800|	5.5	0.032599		Resistance protein candidate (Fragment).
Os05g0571200|	5.5	0.040446	✓	WRKY transcription factor 19.
Os06g0288100|	5.5	0.029935	✓	Receptor-like protein kinase.
Os11g0590700|	5.5	0.024026		Disease resistance protein family protein.
Os09g0481600|	5.5	0.04158		Conserved hypothetical protein.
Os04g0632700|	5.5	0.028052	✓	Protein kinase domain containing protein.
Os04g0328200|	5.4	0.046066		(No Hit)
Os04g0674700|	5.4	0.040335	✓	Amp-binding protein.
Os04g0616700|	5.4	0.038323	✓	BRASSINOSTEROID INSENSITIVE 1-associated receptor kinase 1 precursor (EC 2.7.1.37) (BRI1-associated receptor kinase 1) (Somatic embryogenesis receptor-like kinase 3).
Os01g0940700|	5.4	0.059527		Beta-1,3-glucanase (Fragment).
Os06g0710700|	5.4	0.035018	✓	Benzoyl coenzyme A: benzyl alcohol benzoyl transferase.
Os12g0512400|	5.4	0.028217		Hypothetical protein.
Os06g0589300|	5.4	0.0379		Viral coat and capsid protein family protein.
Os01g0117600|	5.4	0.032011		Protein kinase domain containing protein.
Os05g0224700|	5.4	0.030829	✓	Protein kinase domain containing protein.
Os06g0202300|	5.4	0.075825		Conserved hypothetical protein.
Os09g0498400|	5.4	0.039751		Non-protein coding transcript, uncharacterized transcript.
Os09g0271900|	5.4	0.028052		(No Hit)
Os10g0464000|	5.4	0.036191		Hypersensitive-induced response protein.
Os02g0787700|	5.4	0.033167		Conserved hypothetical protein.
Os02g0211200|	5.3	0.030887		Protein kinase domain containing protein.
Os04g0179200|	5.3	0.032482	✓	Stem secoisolariciresinol dehydrogenase (Fragment).
Os10g0440000|	5.3	0.024357	✓	Cytochrome P450 family protein.
Os12g0512400|	5.3	0.025904		Hypothetical protein.
Os11g0694000|	5.3	0.030882		Hypothetical protein.
Os11g0665600|	5.3	0.06852		Helix-turn-helix, Fis-type domain containing protein.
Os08g0111300|	5.3	0.030557		Transferase family protein.
Os01g0366300|	5.3	0.095375		ARK3 product/receptor-like serine/threonine protein kinase ARK3.
Os06g0225300|	5.3	0.025944		BRASSINOSTEROID INSENSITIVE 1-associated receptor kinase 1 precursor (EC 2.7.1.37) (BRI1-associated receptor kinase 1) (Somatic embryogenesis receptor-like kinase 3).
Os04g0115200|	5.3	0.045874		Conserved hypothetical protein.

Os10g0491000|	5.3	0.038006		Plant Basic Secretory Protein family protein.
Os10g0180800|	5.3	0.024202	✓	EGF-like calcium-binding domain containing protein.
Os03g0610400|	5.3	0.025821	✓	Zn-finger, C2H2 type domain containing protein.
Os09g0467200|	5.3	0.035746	✓	Probable glutathione S-transferase (EC 2.5.1.18) (Auxin-induced protein PGNT1/PCNT110).
Os11g0213000|	5.3	0.033595		Protein kinase domain containing protein.
Os10g0180800|	5.3	0.028939	✓	EGF-like calcium-binding domain containing protein.
Os12g0222800|	5.3	0.0439		(No Hit)
Os01g0115100|	5.3	0.037823		(No Hit)
Os12g0206800|	5.3	0.040562		Transcription factor MADS33.
Os05g0384300|	5.2	0.030882		Peptidase aspartic family protein.
Os09g0350900|	5.2	0.039488		Protein kinase domain containing protein.
Os02g0826800|	5.2	0.029731		(No Hit)
Os11g0228600|	5.2	0.045845	✓	NBS-LRR disease resistance protein homologue.
Os02g0253700|	5.2	0.032297		Conserved hypothetical protein.
Os07g0677200|	5.2	0.040085	✓	Peroxidase.
Os11g0673600|	5.2	0.028939		Disease resistance protein family protein.
Os05g0278500|	5.2	0.037027		Transferase family protein.
Os09g0467200|	5.2	0.033395	✓	Probable glutathione S-transferase (EC 2.5.1.18) (Auxin-induced protein PGNT1/PCNT110).
Os07g0631700|	5.2	0.023187		Calcium-binding EF-hand domain containing protein.
Os04g0632600|	5.2	0.028217	✓	S-locus receptor-like kinase RLK10.
Os09g0325800|	5.2	0.045937	✓	Conserved hypothetical protein.
Os09g0467200|	5.2	0.035916	✓	Probable glutathione S-transferase (EC 2.5.1.18) (Auxin-induced protein PGNT1/PCNT110).
Os10g0536400|	5.1	0.046589	✓	Conserved hypothetical protein.
Os05g0537100|	5.1	0.028217	✓	WRKY transcription factor 10.
Os04g0600300|	5.1	0.039488	✓	Alternative oxidase (OSJNBa0083N12.12 protein).
Os12g0500100|	5.1	0.0379		(No Hit)
Os03g0853200|	5.1	0.058865		CD9/CD37/CD63 antigen family protein.
Os06g0591400|	5.1	0.032482		Conserved hypothetical protein.
Os01g0639600|	5.1	0.034119	✓	Conserved hypothetical protein.
Os08g0163800|	5.1	0.035675		Protein of unknown function DUF260 domain containing protein.
Os10g0456100|	5.1	0.039147	✓	Brn1-like protein.
Os02g0582600|	5.1	0.023187		Heavy metal transport/detoxification protein domain containing protein.
Os07g0106200|	5.1	0.033989	✓	Monosaccharide transporter 3.
Os09g0467200|	5.1	0.032482	✓	Probable glutathione S-transferase (EC 2.5.1.18) (Auxin-induced protein PGNT1/PCNT110).
Os01g0877900|	5.1	0.040033		Hypothetical protein.
Os05g0472700|	5.1	0.057756		Zinc transporter protein ZIP1.

Os10g0469700|	5.1	0.032852	✓	Leucine-rich repeat, cysteine-containing type containing protein.
Os10g0100500|	5.1	0.050848		Protein kinase domain containing protein.
Os11g0168600|	5.1	0.026909	✓	Protein kinase domain containing protein.
Os09g0356200|	5.1	0.033095		Protein kinase domain containing protein.
Os06g0697500|	5.1	0.026444		AAA ATPase, central region domain containing protein.
Os12g0547600|	5.1	0.036983		Hypothetical protein.
Os07g0121800|	5.1	0.023187	✓	Conserved hypothetical protein.
Os10g0180800|	5.0	0.023187	✓	EGF-like calcium-binding domain containing protein.
Os09g0283600|	5.0	0.026444		Zn-finger, cysteine-rich C6HC domain containing protein.
Os03g0690500|	5.0	0.029253		2OG-Fe(II) oxygenase domain containing protein.
Os07g0494800|	5.0	0.027647		Protein kinase domain containing protein.
Os02g0582600|	5.0	0.02334	✓	Heavy metal transport/detoxification protein domain containing protein.
Os06g0582600|	5.0	0.025344	✓	Cysteine proteinase.
Os07g0106200|	5.0	0.026793	✓	Monosaccharide transporter 3.
Os02g0615300|	5.0	0.028939		Protein kinase domain containing protein.
Os05g0181700|	5.0	0.033395	✓	Hypothetical protein.
Os06g0163000|	5.0	0.028541		Heat shock protein STI (Stress inducible protein) (GmSTI).
Os03g0663500|	5.0	0.035596	✓	Thaumatin, pathogenesis-related family protein.
Os03g0748500|	5.0	0.028267		Flavodoxin/nitric oxide synthase domain containing protein.
Os02g0758200|	5.0	0.054731		Conserved hypothetical protein.
Os01g0623500|	5.0	0.033947	✓	AAA ATPase, central region domain containing protein.
Os07g0677200|	5.0	0.029281	✓	Peroxidase.
Os11g0594700|	5.0	0.050376		Protein of unknown function DUF538 family protein.
Os10g0560200|	5.0	0.024357		Uncharacterized Cys-rich domain containing protein.
Os09g0265800|	4.9	0.06956		HGWP repeat containing protein.
Os02g0787600|	4.9	0.034878		Ionotropic glutamate receptor family protein.
Os10g0416500|	4.9	0.047059	✓	Chitinase 1 precursor (EC 3.2.1.14) (Tulip bulb chitinase-1) (TBC-1).
Os01g0118300|	4.9	0.047517		Phosphatidyl serine synthase family protein.
Os07g0677200|	4.9	0.030557	✓	Peroxidase.
Os10g0416500|	4.9	0.053178	✓	Chitinase 1 precursor (EC 3.2.1.14) (Tulip bulb chitinase-1) (TBC-1).
Os08g0201500|	4.9	0.042737	✓	Conserved hypothetical protein.
Os03g0743500|	4.9	0.030781	✓	Calmodulin 1 (Fragment).
Os11g0702400|	4.9	0.059222	✓	Zn-finger, C2H2 type domain containing protein.
Os05g0384600|	4.9	0.038006		ABC transporter related domain containing protein.

Os11g0245500|	4.9	0.043948		Lipid-binding START domain containing protein.
Os05g0472700|	4.9	0.071389		Zinc transporter protein ZIP1.
Os02g0621800|	4.9	0.045033		Conserved hypothetical protein.
Os11g0136300|	4.8	0.033395		Protein of unknown function DUF6 domain containing protein.
Os11g0482200|	4.8	0.035049		Hypothetical protein.
Os01g0905300|	4.8	0.060914		Hypothetical protein.
Os01g0945600|	4.8	0.028939		Amino acid/polyamine transporter I family protein.
Os04g0179200|	4.8	0.029566	✓	Stem secoisolariciresinol dehydrogenase (Fragment).
Os02g0787600|	4.8	0.034884		Ionotropic glutamate receptor family protein.
Os08g0485800|	4.8	0.083281	✓	(No Hit)
Os09g0466300|	4.8	0.034878		GRAM domain containing protein.
Os05g0440100|	4.8	0.02613		Histone deacetylase superfamily protein.
Os05g0519700|	4.8	0.04032		101 kDa heat shock protein.
Os02g0281200|	4.8	0.051027		Disease resistance protein family protein.
Os04g0180400|	4.8	0.032599	✓	Cytochrome P450 CYP99A1 (EC 1.14.-.-) (Fragment).
Os11g0626700|	4.8	0.025716	✓	Hypothetical protein.
Os04g0552700|	4.7	0.034795		Zn-finger, C2H2 type domain containing protein.
Os01g0597600|	4.7	0.076098	✓	Amino acid/polyamine transporter II family protein.
Os03g0130100|	4.7	0.049542	✓	AMP-binding protein (Adenosine monophosphate binding protein 5 AMPBP5).
Os07g0121600|	4.7	0.041246		Conserved hypothetical protein.
Os12g0556200|	4.7	0.044838		Hypothetical protein.
Os06g0494400|	4.7	0.026939		Multi antimicrobial extrusion protein MatE family protein.
Os02g0585200|	4.7	0.023187	✓	Heavy metal transport/detoxification protein domain containing protein.
Os08g0291800|	4.7	0.044172		(No Hit)
Os03g0134500|	4.7	0.055033		Cytochrome p450 (CYP78A9).
Os01g0905200|	4.7	0.030913		Exo70 exocyst complex subunit family protein.
Os02g0153900|	4.7	0.030781	✓	Protein kinase domain containing protein.
Os01g0567200|	4.7	0.09213	✓	Conserved hypothetical protein.
Os11g0117600|	4.7	0.04924	✓	WRKY transcription factor 50 (Fragment).
Os01g0678800|	4.7	0.033482		Heavy metal transport/detoxification protein domain containing protein.
Os02g0216300|	4.7	0.036423		Conserved hypothetical protein.
Os06g0581500|	4.7	0.030257	✓	Protein kinase domain containing protein.
Os01g0117400|	4.7	0.028052		Protein kinase domain containing protein.
Os06g0649000|	4.6	0.059005		WRKY transcription factor 28.
Os08g0335500|	4.6	0.039147		(No Hit)
Os03g0117600|	4.6	0.05152		(No Hit)

Os08g0408300|	4.6	0.040335		Conserved hypothetical protein.
Os08g0518800|	4.6	0.062159		Chitinase (EC 3.2.1.14).
Os10g0419400|	4.6	0.023187		Submergence induced protein 2.
Os05g0407400|	4.6	0.055058		(No Hit)
Os01g0884400|	4.6	0.028939	✓	U box domain containing protein.
Os04g0136600|	4.6	0.061965	✓	(No Hit)
Os01g0736500|	4.6	0.030557		Harpin-induced 1 domain containing protein.
Os07g0127700|	4.6	0.051349		Pathogenesis-related protein 1.
Os04g0416900|	4.6	0.024211		Digalactosyldiacylglycerol synthase 1.
Os08g0230800|	4.5	0.032204	✓	(No Hit)
Os07g0582400|	4.5	0.039343	✓	Sorbitol transporter.
Os02g0271600|	4.5	0.037823		Peptidase S8 and S53, subtilisin, kexin, sedolisin domain containing protein.
Os01g0905200|	4.5	0.024357		Exo70 exocyst complex subunit family protein.
Os07g0582400|	4.5	0.036727	✓	Sorbitol transporter.
Os06g0587300|	4.5	0.02334		Conserved hypothetical protein.
Os09g0354300|	4.5	0.036597		2OG-Fe(II) oxygenase domain containing protein.
Os02g0306300|	4.5	0.039681		Conserved hypothetical protein.
Os02g0687200|	4.5	0.037179		Protein of unknown function DUF581 family protein.
Os09g0339000|	4.5	0.04244		Protein kinase domain containing protein.
Os06g0163000|	4.5	0.053093		Heat shock protein STI (Stress inducible protein) (GmSTI).
Os02g0216300|	4.5	0.04247		Conserved hypothetical protein.
Os09g0498200|	4.5	0.032011		Conserved hypothetical protein.
Os05g0555700|	4.5	0.033692		Conserved hypothetical protein.
Os02g0198500|	4.5	0.057168		Conserved hypothetical protein.
Os02g0216300|	4.5	0.035596		Conserved hypothetical protein.
Os07g0677200|	4.5	0.036494	✓	Peroxidase.
Os04g0176100|	4.4	0.037081		Caffeic acid 3-O-methyltransferase (EC 2.1.1.68) (S-adenosysl-L- methionine:caffeic acid 3-O-methyltransferase) (COMT) (CAOMT).
Os10g0416500|	4.4	0.046861	✓	Chitinase 1 precursor (EC 3.2.1.14) (Tulip bulb chitinase-1) (TBC-1).
Os08g0473900|	4.4	0.025319		Alpha-amylase type B (Fragment).
Os08g0485800|	4.4	0.06395	✓	(No Hit)
Os07g0614600|	4.4	0.034663		Lung seven transmembrane receptor family protein.
Os03g0150800|	4.4	0.037536	✓	High affinity phosphate transporter 2 (Phosphate transporter).
Os04g0175400|	4.4	0.038006	✓	Conserved hypothetical protein.
Os01g0152000|	4.4	0.037216	✓	Protein kinase domain containing protein.
Os04g0431700|	4.4	0.032599		(No Hit)
Os04g0178300|	4.4	0.024357	✓	Copalyl diphosphate synthetase (Fragment).
Os03g0575200|	4.4	0.042183		K+ potassium transporter family protein.

Os01g0176800|	4.4	0.040049		(No Hit)
Os07g0582400|	4.4	0.035821	✓	Sorbitol transporter.
Os02g0615500|	4.4	0.028939		Protein kinase domain containing protein.
Os08g0395700|	4.4	0.046771		Conserved hypothetical protein.
Os08g0412800|	4.4	0.055427	✓	Protein of unknown function DUF1262 family protein.
Os04g0217300|	4.4	0.066474		(No Hit)
Os04g0664900|	4.3	0.053093		Cell wall invertase (EC 3.2.1.26).
Os11g0232500|	4.3	0.044133		(No Hit)
Os12g0281300|	4.3	0.044388		Pi-ta protein.
Os08g0316700|	4.3	0.040865		(No Hit)
Os09g0494600|	4.3	0.036051		Protein of unknown function DUF599 family protein.
Os07g0677200|	4.3	0.029457	✓	Peroxidase.
Os03g0383900|	4.3	0.037823		Heavy metal transport/detoxification protein domain containing protein.
Os08g0352100|	4.3	0.030887		Conserved hypothetical protein.
Os02g0676600|	4.3	0.043176		Protein of unknown function DUF635 family protein.
Os01g0890300|	4.3	0.042025		Conserved hypothetical protein.
Os04g0538000|	4.3	0.041967		TPR repeat containing protein.
Os02g0561000|	4.3	0.023187		(No Hit)
Os07g0677200|	4.3	0.036597	✓	Peroxidase.
Os10g0419400|	4.3	0.03017		Submergence induced protein 2.
Os05g0560500|	4.3	0.036945		Hypothetical protein.
Os09g0315100|	4.2	0.045106		Ribonuclease III domain containing protein.
Os07g0650600|	4.2	0.02613		BLE2 protein.
Os01g0371500|	4.2	0.030882		Glutathione-S-transferase 19E50.
Os06g0229000|	4.2	0.037216		FtsH protease (VAR2) (Zinc dependent protease).
Os02g0211000|	4.2	0.033435		Viral coat and capsid protein family protein.
Os12g0135800|	4.2	0.071389		Esterase/lipase/thioesterase domain containing protein.
Os10g0444700|	4.2	0.061488		Phosphate transporter 6.
Os12g0556200|	4.2	0.045602	✓	Hypothetical protein.
Os02g0615500|	4.2	0.031156		Protein kinase domain containing protein.
Os03g0150800|	4.2	0.034986	✓	High affinity phosphate transporter 2 (Phosphate transporter).
Os03g0380600|	4.2	0.040085		En/Spm-like transposon proteins family protein.
Os05g0530400|	4.2	0.032011	✓	Heat shock factor protein 1 (HSF 1) (Heat shock transcription factor 1) (HSTF 1).
Os06g0200700|	4.2	0.036416		Conserved hypothetical protein.
Os11g0539500|	4.2	0.048834		O-methyltransferase, family 2 domain containing protein.
Os09g0506000|	4.2	0.0486	✓	Diphosphonucleotide phosphatase 1 precursor.
Os01g0891700|	4.2	0.042771		Receptor-like protein CLAVATA2.

Os11g0135000|	4.2	0.050116		Major facilitator superfamily antiporter.
Os12g0272800|	4.1	0.039661		(No Hit)
Os01g0955100|	4.1	0.033441	✓	Regulator of gene silencing.
Os05g0519700|	4.1	0.029566		101 kDa heat shock protein.
Os09g0506000|	4.1	0.051683	✓	Diphosphonucleotide phosphatase 1 precursor.
Os01g0117000|	4.1	0.028939		Protein kinase domain containing protein.
Os01g0584900|	4.1	0.032542	✓	WRKY transcription factor 67.
Os08g0200500|	4.1	0.032011	✓	Protein kinase domain containing protein.
Os09g0434900|	4.1	0.03226		Zn-finger, cysteine-rich C6HC domain containing protein.
Os03g0150800|	4.1	0.036494	✓	High affinity phosphate transporter 2 (Phosphate transporter).
Os08g0158000|	4.1	0.062673		(No Hit)
Os01g0314800|	4.1	0.052388	✓	Late embryogenesis abundant protein 3 family protein.
Os05g0530400|	4.1	0.030378	✓	Heat shock factor protein 1 (HSF 1) (Heat shock transcription factor 1) (HSTF 1).
Os07g0534700|	4.1	0.044366		Protein kinase family protein.
Os01g0178900|	4.1	0.024211		Hypothetical protein.
Os01g0946600|	4.1	0.060924		Glucan endo-1,3-beta-glucosidase GV (EC 3.2.1.39) ((1->3)-beta-glucan endohydrolase GV) ((1->3)-beta-glucanase isoenzyme GV) (Beta-1,3-endoglucanase GV).
Os06g0129100|	4.1	0.065499	✓	Short-chain dehydrogenase/reductase SDR family protein.
Os01g0824700|	4.1	0.036051		Cyclin-like F-box domain containing protein.
Os01g0768100|	4.1	0.028939	✓	Hypothetical protein.
Os07g0130800|	4.1	0.034884	✓	Lectin-like receptor kinase 7;2.
Os05g0240200|	4.1	0.084491		Disease resistance protein family protein.
Os08g0480400|	4.1	0.037838		(No Hit)
Os05g0530400|	4.0	0.035529	✓	Heat shock factor protein 1 (HSF 1) (Heat shock transcription factor 1) (HSTF 1).
Os01g0508500|	4.0	0.052378	✓	Hypothetical protein.
Os04g0630900|	4.0	0.040033		Anthocyanidin reductase.
Os11g0592000|	4.0	0.029457		Barwin.
Os03g0583900|	4.0	0.033947		Type III restriction enzyme, res subunit family protein.
Os02g0154000|	4.0	0.039147	✓	Protein kinase domain containing protein.
Os01g0846500|	4.0	0.038706		PAP/25A core domain containing protein.
Os08g0353700|	4.0	0.030805		Conserved hypothetical protein.
Os12g0130300|	4.0	0.064525		Resistance protein candidate (Fragment).
Os02g0584700|	4.0	0.089321	✓	(No Hit)
Os10g0189200|	4.0	0.059326		(No Hit)
Os03g0836300|	4.0	0.029566		Harpin-induced 1 domain containing protein.

Os02g0560600|	4.0	0.029879	✓	(No Hit)
Os09g0327600|	4.0	0.028052		Non-protein coding transcript, putative npRNA.
Os01g0114500|	4.0	0.036798		Rust resistance kinase Lr10.
Os01g0722700|	4.0	0.028217		Hexokinase.
Os02g0271000|	4.0	0.036774		Peptidase S8 and S53, subtilisin, kexin, sedolisin domain containing protein.
Os07g0230800|	4.0	0.060353		(No Hit)
Os03g0803500|	4.0	0.047314		2OG-Fe(II) oxygenase domain containing protein.
Os09g0313600|	4.0	0.030317		(No Hit)
Os02g0559800|	4.0	0.025768	✓	Zn-finger, RING domain containing protein.
Os03g0150800|	4.0	0.036774	✓	High affinity phosphate transporter 2 (Phosphate transporter).
Os01g0130200|	3.9	0.044761	✓	(No Hit)
Os06g0192800|	3.9	0.044777	✓	Zn-finger, RING domain containing protein.
Os01g0690000|	3.9	0.023187	✓	Conserved hypothetical protein.
Os04g0495500|	3.9	0.030317		Hypothetical protein.
Os07g0650600|	3.9	0.041032		BLE2 protein.
Os04g0667600|	3.9	0.039135	✓	Heavy metal transport/detoxification protein domain containing protein.
Os02g0727000|	3.9	0.084955		(No Hit)
Os02g0561800|	3.9	0.026444		(No Hit)
Os11g0477400|	3.9	0.036798		Transposase (Fragment).
Os12g0153800|	3.9	0.046582		Heat shock protein Hsp70 family protein.
Os01g0114300|	3.9	0.031493		LRK14.
Os10g0562900|	3.9	0.067393	✓	Pathogenesis-related transcriptional factor and ERF domain containing protein.
Os08g0508800|	3.9	0.028939	✓	Lipoxygenase, chloroplast precursor (EC 1.13.11.12).
Os01g0657800|	3.9	0.047942		(No Hit)
Os03g0150800|	3.9	0.049743	✓	High affinity phosphate transporter 2 (Phosphate transporter).
Os03g0830200|	3.9	0.023187		Uncharacterized Cys-rich domain containing protein.
Os03g0830500|	3.9	0.028939		PGPS/D12.
Os09g0357400|	3.9	0.033595		Disease resistance protein family protein.
Os02g0228400|	3.9	0.040251	✓	Conserved hypothetical protein.
Os02g0228300|	3.8	0.034878		Protein kinase domain containing protein.
Os10g0558700|	3.8	0.034295	✓	2OG-Fe(II) oxygenase domain containing protein.
Os07g0596500|	3.8	0.080045		(No Hit)
Os11g0600900|	3.8	0.042629		Mannose-6-phosphate isomerase (ManA).
Os07g0635300|	3.8	0.038644	✓	E-class P450, group I family protein.
Os05g0530400|	3.8	0.032599	✓	Heat shock factor protein 1 (HSF 1) (Heat shock transcription factor 1) (HSTF 1).
Os01g0846500|	3.8	0.040033		PAP/25A core domain containing protein.

Os01g0113800|	3.8	0.023975	✓	Protein kinase family protein.
Os07g0130800|	3.8	0.024357	✓	Lectin-like receptor kinase 7;2.
Os08g0514200|	3.8	0.054997		(No Hit)
Os02g0138900|	3.8	0.025944		Low affinity calcium antiporter CAX2.
Os02g0561400|	3.8	0.028052		(No Hit)
Os01g0941400|	3.8	0.053568	✓	Beta-1,3-glucanase precursor.
Os04g0600300|	3.8	0.059373	✓	Alternative oxidase (OSJNBa0083N12.12 protein).
Os07g0653000|	3.8	0.032338		BLE2 protein.
Os06g0619000|	3.8	0.044761		Disease resistance protein family protein.
Os08g0335500|	3.8	0.044805		(No Hit)
Os02g0235500|	3.8	0.076862		(No Hit)
Os01g0256300|	3.8	0.033374		Protein kinase-like domain containing protein.
Os03g0236200|	3.8	0.030317	✓	Glutamate decarboxylase isozyme 3 (EC 4.1.1.15).
Os01g0200300|	3.8	0.04663		Hypothetical protein.
Os06g0614100|	3.8	0.036328		Basic-leucine zipper (bZIP) transcription factor domain containing protein.
Os04g0308100|	3.8	0.040033		Wall-associated kinase 4.
Os08g0229000|	3.8	0.074543		(No Hit)
Os11g0540600|	3.8	0.028939		Plant protein of unknown function family protein.
Os05g0549900|	3.8	0.031696		Lipid phosphate phosphatase 2 (EC 3.1.3.-) (AtLPP2) (Phosphatidic acid phosphatase 2) (AtPAP2) (Prenyl diphosphate phosphatase).
Os01g0246700|	3.8	0.043375	✓	WRKY transcription factor 1.
Os04g0524500|	3.8	0.039803	✓	Oligopeptide transporter OPT superfamily protein.
Os08g0480400|	3.7	0.050198		(No Hit)
Os09g0383500|	3.7	0.044499		(No Hit)
Os10g0120300|	3.7	0.056562		Leucine-rich repeat, plant specific containing protein.
Os01g0515300|	3.7	0.036945		Protein kinase domain containing protein.
Os12g0145400|	3.7	0.027037		Protein of unknown function DUF231 domain containing protein.
Os06g0128800|	3.7	0.048459	✓	C2 calcium/lipid-binding region, CaLB domain containing protein.
Os05g0371800|	3.7	0.077148		(No Hit)
Os04g0266600|	3.7	0.084973		(No Hit)
Os01g0686000|	3.7	0.02829		Conserved hypothetical protein.
Os07g0134000|	3.7	0.073821		Amino acid permease 6.
Os01g0763700|	3.7	0.031428		Exo70 exocyst complex subunit family protein.
Os08g0473900|	3.7	0.033395		Alpha-amylase type B (Fragment).
Os06g0546200|	3.7	0.062616		(No Hit)
Os06g0635600|	3.7	0.086609		(No Hit)
Os04g0150700|	3.7	0.044382		(No Hit)
Os10g0562200|	3.7	0.043948		Lipase-like protein.

Os01g0273800|	3.7	0.04928		Flavin-containing monooxygenase FMO family protein.
Os01g0728300|	3.7	0.029457		Cytochrome P450 monooxygenase CYP72A5 (Fragment).
Os06g0726200|	3.7	0.07081	✓	Endochitinase precursor (EC 3.2.1.14).
Os12g0630500|	3.7	0.048108	✓	Antifungal protein R (Fragment).
Os09g0488600|	3.7	0.075589		(No Hit)
Os08g0509100|	3.7	0.039147		Lipoxygenase, chloroplast precursor (EC 1.13.11.12).
Os03g0610400|	3.7	0.03032	✓	Zn-finger, C2H2 type domain containing protein.
Os01g0163000|	3.7	0.028939	✓	Leucine-rich repeat, typical subtype containing protein.
Os05g0367400|	3.7	0.033595	✓	Thiamine pyrophosphokinase family protein.
Os02g0560200|	3.7	0.028939	✓	(No Hit)
Os06g0726200|	3.7	0.05728	✓	Endochitinase precursor (EC 3.2.1.14).
Os11g0684700|	3.6	0.039181		(No Hit)
Os08g0185900|	3.6	0.040144	✓	Ubiquitin domain containing protein.
Os11g0133100|	3.6	0.075608		Protein kinase family protein.
Os09g0569700|	3.6	0.032085		Hypothetical protein.
Os01g0114300|	3.6	0.029504		LRK14.
Os08g0473900|	3.6	0.033395		Alpha-amylase type B (Fragment).
Os02g0550800|	3.6	0.029457		Ammonium transporter.
Os05g0519700|	3.6	0.036494		101 kDa heat shock protein.
Os09g0434900|	3.6	0.039661		Zn-finger, cysteine-rich C6HC domain containing protein.
Os08g0400000|	3.6	0.035225		Puromycin-sensitive aminopeptidase (EC 3.4.11.-) (PSA).
Os08g0428200|	3.6	0.041761		Typical P-type R2R3 Myb protein (Fragment).
Os07g0539900|	3.6	0.028217	✓	Beta-1,3-glucanase-like protein.
Os04g0141200|	3.6	0.084949		Legume lectin, beta domain containing protein.
Os02g0818900|	3.6	0.046127		Heavy metal transport/detoxification protein domain containing protein.
Os06g0584400|	3.6	0.067745		Hypothetical protein.
Os12g0548800|	3.6	0.045038		ARM repeat fold domain containing protein.
Os01g0841700|	3.6	0.052096	✓	RPP17-1.
Os07g0593600|	3.6	0.076002		(No Hit)
Os01g0350100|	3.6	0.043235		(No Hit)
Os07g0623800|	3.6	0.094161		(No Hit)
Os01g0674400|	3.6	0.023187	✓	(No Hit)
Os03g0276500|	3.6	0.032482		Heat shock protein 70.
Os02g0155300|	3.6	0.028052		Conserved hypothetical protein.
Os01g0763700|	3.6	0.03131		Exo70 exocyst complex subunit family protein.
Os04g0644700|	3.6	0.033516		Coatomer epsilon subunit family protein.

Os01g0946600|	3.6	0.055609		Glucan endo-1,3-beta-glucosidase GV (EC 3.2.1.39) ((1->3)-beta-glucan endohydrolase GV) ((1->3)-beta-glucanase isoenzyme GV) (Beta-1,3-endoglucanase GV).
Os09g0266000|	3.6	0.057592		Nuclear transport factor 2 domain containing protein.
Os07g0537400|	3.6	0.033167		Serine/threonine kinase receptor precursor.
Os03g0793000|	3.6	0.024357		Zn-finger, A20-like domain containing protein.
Os07g0650600|	3.6	0.02829		BLE2 protein.
Os12g0609200|	3.6	0.052149		Hypothetical protein.
Os03g0782300|	3.6	0.039488		Conserved hypothetical protein.
Os09g0431100|	3.6	0.052388		Ionotropic glutamate receptor family protein.
Os01g0605100|	3.6	0.03562	✓	BCS1 protein-like protein.
Os06g0225300|	3.6	0.045937		BRASSINOSTEROID INSENSITIVE 1-associated receptor kinase 1 precursor (EC 2.7.1.37) (BRI1-associated receptor kinase 1) (Somatic embryogenesis receptor-like kinase 3).
Os03g0197200|	3.6	0.051363		Sorbitol transporter.
Os05g0486600|	3.6	0.028939		(No Hit)
Os09g0514500|	3.6	0.032076		Hypothetical protein.
Os06g0676700|	3.5	0.052743		High pI alpha-glucosidase.
Os01g0860500|	3.5	0.046519	✓	Chitinase (EC 3.2.1.14).
Os01g0293000|	3.5	0.024357		S-adenosylmethionine synthetase 1 (EC 2.5.1.6) (Methionine adenosyltransferase 1) (AdoMet synthetase 1).
Os01g0393400|	3.5	0.046899		Hypothetical protein.
Os09g0474000|	3.5	0.045339		Basic-leucine zipper (bZIP) transcription factor domain containing protein.
Os03g0449000|	3.5	0.039147		(No Hit)
Os06g0560000|	3.5	0.032482		Ferroportin1 family protein.
Os06g0554600|	3.5	0.033947	✓	Hypothetical protein.
Os04g0339000|	3.5	0.038644	✓	Cytochrome P450 family protein.
Os01g0127700|	3.5	0.034986	✓	Protein kinase domain containing protein.
Os10g0114300|	3.5	0.039488		Aldo/keto reductase family protein.
Os12g0562400|	3.5	0.030805		Phospholipase C (Fragment).
Os07g0417500|	3.5	0.033374		(No Hit)
Os09g0452900|	3.5	0.039488	✓	Glycosyl transferase, family 31 protein.
Os05g0486600|	3.5	0.023187		(No Hit)
Os03g0189300|	3.5	0.049704		Conserved hypothetical protein.
Os11g0691800|	3.5	0.055116		(No Hit)
Os11g0686400|	3.5	0.028217		Disease resistance protein family protein.
Os03g0575200|	3.5	0.029566		K+ potassium transporter family protein.
Os05g0138800|	3.5	0.031071		(No Hit)
Os01g0115100|	3.5	0.046519		(No Hit)

Os01g0882800	3.5	0.033763	✓	Amino acid carrier.
Os06g0602500	3.5	0.02334	✓	Protein kinase domain containing protein.
Os06g0726200	3.5	0.034884	✓	Endochitinase precursor (EC 3.2.1.14).
Os06g0681000	3.5	0.034918	✓	Hypothetical protein.
Os10g0540800	3.5	0.058996		Hypothetical protein.
Os07g0690900	3.5	0.026444	✓	Phytochelatin synthetase-like conserved region family protein.
Os09g0364700	3.5	0.07655		(No Hit)
Os10g0394000	3.5	0.027037		Lipolytic enzyme, G-D-S-L family protein.
Os08g0205100	3.5	0.046557		NBS-LRR disease resistance protein homologue (Fragment).
Os12g0149900	3.5	0.039855		IQ calmodulin-binding region domain containing protein.
Os10g0136500	3.5	0.032204		Protein kinase domain containing protein.
Os10g0404900	3.5	0.05027		Homeobox-leucine zipper protein HAT5 (HD-ZIP protein 5) (HD-ZIP protein ATHB-1).
Os08g0178400	3.5	0.037948		Non-protein coding transcript, unclassifiable transcript.
Os03g0110000	3.5	0.033947		(No Hit)
Os08g0141400	3.5	0.04366	✓	FAD-dependent pyridine nucleotide-disulphide oxidoreductase domain containing protein.
Os11g0692100	3.5	0.086897		Bacterial blight resistance protein.
Os02g0485100	3.5	0.036494		(No Hit)
Os06g0671300	3.5	0.04079	✓	Cytochrome P450 family protein.
Os06g0715700	3.4	0.040179		Protein of unknown function DUF803 family protein.
Os01g0114100	3.4	0.050773		Protein kinase family protein.
Os01g0250000	3.4	0.071733		HGWP repeat containing protein.
Os07g0536100	3.4	0.090585		(No Hit)
Os01g0248500	3.4	0.050775		Pathogen-related protein.
Os10g0134500	3.4	0.043089		Protein kinase-like domain containing protein.
Os07g0539900	3.4	0.030396	✓	Beta-1,3-glucanase-like protein.
Os06g0690200	3.4	0.023187	✓	Protein kinase domain containing protein.
Os06g0159600	3.4	0.03131		U box domain containing protein.
Os04g0175600	3.4	0.04307		Caffeic acid O-methyltransferase (EC 2.1.1.6).
Os07g0654400	3.4	0.024357		BLE2 protein.
Os05g0233400	3.4	0.044247		(No Hit)
Os05g0209500	3.4	0.024357		Conserved hypothetical protein.
Os01g0831700	3.4	0.047542		(No Hit)
Os10g0510800	3.4	0.034938		(No Hit)
Os02g0733500	3.4	0.027647	✓	Parvalbumin family protein.
Os04g0349700	3.4	0.023187		Leucine-rich repeat, typical subtype containing protein.
Os04g0370100	3.4	0.067826		EGF-like calcium-binding domain containing protein.

Os08g0258500	3.4	0.033095		(No Hit)
Os03g0582300	3.4	0.029566		Non-protein coding transcript, uncharacterized transcript.
Os03g0424900	3.4	0.034195		(No Hit)
Os01g0137800	3.4	0.036051		Non-protein coding transcript, unclassifiable transcript.
Os12g0202700	3.4	0.054354		O-methyltransferase, family 2 protein.
Os01g0781700	3.4	0.044577		Disease resistance protein family protein.
Os11g0162700	3.4	0.036011		Non-protein coding transcript, uncharacterized transcript.
Os05g0519700	3.4	0.035225		101 kDa heat shock protein.
Os10g0132300	3.4	0.04109		Jacalin-related lectin domain containing protein.
Os05g0256100	3.4	0.056284		Protein kinase domain containing protein.
Os04g0120000	3.4	0.037437		Hydroxyproline-rich glycoprotein-like.
Os12g0149900	3.4	0.037216		IQ calmodulin-binding region domain containing protein.
Os03g0288000	3.4	0.071324	✓	Metallothionein-like protein 1 (MT-1).
Os09g0381800	3.4	0.045649		(No Hit)
Os09g0526600	3.4	0.036494		Heat shock factor protein 3 (HSF 3) (Heat shock transcription factor 3) (HSTF 3).
Os02g0162900	3.4	0.036382		(No Hit)
Os01g0860500	3.4	0.030805	✓	Chitinase (EC 3.2.1.14).
Os12g0516000	3.4	0.039488		Hypothetical protein.
Os07g0250900	3.4	0.023187	✓	Harpin-induced 1 domain containing protein.
Os02g0736200	3.4	0.068952	✓	RNA-directed RNA polymerase (EC 2.7.7.48).
Os11g0625200	3.4	0.061451		Protein kinase family protein.
Os09g0288700	3.4	0.071468		(No Hit)
Os06g0613800	3.4	0.02851		Peptidase aspartic family protein.
Os07g0650600	3.4	0.029372		BLE2 protein.
Os08g0367000	3.4	0.039803		En/Spm-like transposon proteins family protein.
Os03g0142700	3.3	0.087838		(No Hit)
Os03g0626600	3.3	0.041934		Ubiquitin interacting motif domain containing protein.
Os07g0115900	3.3	0.033395		(No Hit)
Os01g0598300	3.3	0.038835		(No Hit)
Os11g0284700	3.3	0.036597		(No Hit)
Os06g0159600	3.3	0.028632		U box domain containing protein.
Os01g0532900	3.3	0.063673		Callose synthase 1 catalytic subunit.
Os07g0537300	3.3	0.037165		Protein of unknown function DUF26 domain containing protein.
Os12g0120400	3.3	0.04591		ATPase-like protein.
Os12g0109200	3.3	0.028052		Ca(2+)-dependent nuclease.
Os05g0253200	3.3	0.023187		Protein kinase domain containing protein.

Os10g0444700|	3.3	0.041411		Phosphate transporter 6.
Os01g0735500|	3.3	0.027136		Conserved hypothetical protein.
Os12g0531300|	3.3	0.030882		(No Hit)
Os03g0319000|	3.3	0.028939	✓	Hypothetical protein.
Os06g0319100|	3.3	0.079657		(No Hit)
Os04g0202300|	3.3	0.04244		Protein kinase family protein.
Os06g0607600|	3.3	0.07848		(No Hit)
Os04g0631000|	3.3	0.058805		Anthocyanidin reductase.
Os07g0654700|	3.3	0.024357		(No Hit)
Os11g0579900|	3.3	0.028694		ARM repeat fold domain containing protein.
Os01g0214500|	3.3	0.030805		Conserved hypothetical protein.
Os02g0216300|	3.3	0.030882		Conserved hypothetical protein.
Os12g0149900|	3.3	0.037037		IQ calmodulin-binding region domain containing protein.
Os01g0563000|	3.3	0.035408		Peptidylprolyl isomerase, FKBP-type domain containing protein.
Os03g0835200|	3.3	0.040871		Hypothetical protein.
Os01g0697000|	3.3	0.040872		(No Hit)
Os11g0687100|	3.3	0.096839	✓	von Willebrand factor, type A domain containing protein.
Os11g0135000|	3.3	0.042922		Major facilitator superfamily antiporter.
Os06g0541600|	3.3	0.042429		Protein kinase family protein.
Os12g0203200|	3.3	0.048808		Hypothetical protein.
Os11g0496500|	3.2	0.050813		AT.I.24-5 protein (Fragment).
Os09g0419600|	3.2	0.039488		Zn-finger, cysteine-rich C6HC domain containing protein.
Os02g0632800|	3.2	0.054165	✓	Protein kinase domain containing protein.
Os01g0695800|	3.2	0.035872	✓	Multidrug resistance protein 1 homolog.
Os12g0281300|	3.2	0.028269		Pi-ta protein.
Os02g0612200|	3.2	0.02847		NB-ARC domain containing protein.
Os07g0543500|	3.2	0.032599		Conserved hypothetical protein.
Os01g0674400|	3.2	0.023187	✓	(No Hit)
Os11g0154300|	3.2	0.049501		Protein of unknown function DUF584 family protein.
Os01g0940800|	3.2	0.090512	✓	Beta-1,3-glucanase precursor.
Os08g0127800|	3.2	0.032599		Conserved hypothetical protein.
Os05g0465000|	3.2	0.093728	✓	Conserved hypothetical protein.
Os09g0570000|	3.2	0.04391		Protein kinase domain containing protein.
Os03g0754400|	3.2	0.038194		(No Hit)
Os10g0390600|	3.2	0.024357		1-aminocyclopropane-1-carboxylate synthase family protein.
Os01g0364500|	3.2	0.033758		(No Hit)
Os01g0222800|	3.2	0.039488		Curculin-like (mannose-binding) lectin domain containing protein.

Os04g0629300	3.2	0.02822		SNF2-related domain containing protein.
Os05g0384600	3.2	0.048606		ABC transporter related domain containing protein.
Os03g0583900	3.2	0.053382		Type III restriction enzyme, res subunit family protein.
Os01g0944900	3.2	0.024357	✓	Beta-1,3-glucanase precursor.
Os05g0330900	3.2	0.090181		(No Hit)
Os03g0277300	3.2	0.033374		Heat shock protein 70.
Os12g0281300	3.2	0.029766		Pi-ta protein.
Os05g0557400	3.2	0.024357		Membrane attack complex component/perforin/complement C9 family protein.
Os01g0778600	3.2	0.039147		(No Hit)
Os10g0563800	3.2	0.040826	✓	Conserved hypothetical protein.
Os01g0940800	3.2	0.084629	✓	Beta-1,3-glucanase precursor.
Os03g0743500	3.2	0.036774	✓	Calmodulin 1 (Fragment).
Os04g0629300	3.2	0.031915		SNF2-related domain containing protein.
Os05g0365700	3.2	0.030434		Glycoside hydrolase, family 1 protein.
Os01g0197900	3.2	0.027401		Hypothetical protein.
Os01g0332000	3.2	0.037216		(No Hit)
Os08g0125100	3.2	0.075293		(No Hit)
Os05g0587500	3.2	0.033947		Peptidase aspartic family protein.
Os11g0130400	3.2	0.042401		Harpin-induced 1 domain containing protein.
Os02g0282000	3.2	0.056398		Disease resistance protein family protein.
Os01g0514800	3.2	0.030708		(No Hit)
Os05g0526700	3.2	0.038428		Harpin-induced 1 domain containing protein.
Os02g0216300	3.2	0.039018		Conserved hypothetical protein.
Os02g0809800	3.2	0.044761		SPX, N-terminal domain containing protein.
Os02g0216300	3.2	0.02847		Conserved hypothetical protein.
Os03g0342000	3.1	0.086336		(No Hit)
Os06g0560000	3.1	0.036983		Ferroportin1 family protein.
Os04g0543200	3.1	0.036494		RNA-binding region RNP-1 (RNA recognition motif) domain containing protein.
Os11g0660700	3.1	0.07329		Hypothetical protein.
Os01g0763700	3.1	0.043176		Exo70 exocyst complex subunit family protein.
Os07g0533800	3.1	0.061047		Peptidase A1, pepsin family protein.
Os11g0687200	3.1	0.094624	✓	Hypothetical protein.
Os11g0692500	3.1	0.047392		Bacterial blight resistance protein.
Os03g0321700	3.1	0.04178	✓	WRKY transcription factor 55.
Os12g0537900	3.1	0.041273		(No Hit)
Os02g0118900	3.1	0.056485		Disease resistance protein family protein.
Os01g0826400	3.1	0.043234	✓	WRKY transcription factor 24.
Os11g0674500	3.1	0.0421		NBS-LRR-like protein D.
Os11g0694400	3.1	0.052821		(No Hit)

Os12g0555300	3.1	0.044926	✓	Probenazole-inducible protein PBZ1.
Os02g0216300	3.1	0.029457		Conserved hypothetical protein.
Os12g0634400	3.1	0.077228		Hypothetical protein.
Os06g0726200	3.1	0.03032	✓	Endochitinase precursor (EC 3.2.1.14).
Os01g0965900	3.1	0.04032		Conserved hypothetical protein.
Os04g0307500	3.1	0.029991		Protein kinase domain containing protein.
Os11g0522300	3.1	0.037216		(No Hit)
Os01g0931000	3.1	0.03562		Glucose/ribitol dehydrogenase family protein.
Os08g0298700	3.1	0.037957		Male sterility protein family protein.
Os07g0274000	3.1	0.035596		Viral coat and capsid protein family protein.
Os08g0378000	3.1	0.042255	✓	Conserved hypothetical protein.
Os03g0696300	3.1	0.071468		CCAAT-binding transcription factor, subunit B family protein.
Os02g0227100	3.1	0.044178	✓	Conserved hypothetical protein.
Os02g0434800	3.1	0.032011		(No Hit)
Os07g0130600	3.1	0.039392	✓	Protein kinase family protein.
Os10g0162200	3.1	0.035596		Zn-finger, CCHC type domain containing protein.
Os07g0419300	3.1	0.036051		Thaumatin-like protein 1a precursor (Allergen Mal d 2) (Mdtl1) (Pathogenesis-related protein 5a) (PR-5a).
Os01g0841700	3.1	0.075146	✓	RPP17-1.
Os07g0650600	3.1	0.028632		BLE2 protein.
Os03g0276500	3.1	0.030257		Heat shock protein 70.
Os08g0473900	3.1	0.032457		Alpha-amylase type B (Fragment).
Os08g0473800	3.1	0.037165		Conserved hypothetical protein.
Os02g0124000	3.1	0.036212		(No Hit)
Os01g0563000	3.1	0.03901		Peptidylprolyl isomerase, FKBP-type domain containing protein.
Os03g0597600	3.1	0.046798		L-asparaginase (EC 3.5.1.1) (L-asparagine amidohydrolase).
Os01g0902700	3.1	0.058791	✓	TGF-beta receptor, type I/II extracellular region family protein.
Os07g0190800	3.1	0.041375		Thioredoxin.
Os01g0563000	3.1	0.049704		Peptidylprolyl isomerase, FKBP-type domain containing protein.
Os09g0569800	3.1	0.032268	✓	Protein kinase domain containing protein.
Os06g0321300	3.0	0.096495		(No Hit)
Os01g0346400	3.0	0.036011		Conserved hypothetical protein.
Os02g0703300	3.0	0.045152	✓	Protein of unknown function DUF1218 family protein.
Os11g0227800	3.0	0.069992	✓	Disease resistance protein family protein.
Os03g0315300	3.0	0.031074		(No Hit)

Os01g0300900|	3.0	0.056297		Galactose oxidase, central domain containing protein.
Os01g0602500|	3.0	0.096794	✓	E-class P450, group I family protein.
Os11g0626700|	3.0	0.049343		Hypothetical protein.
Os08g0523600|	3.0	0.033095		CTV.22.
Os12g0248600|	3.0	0.06034	✓	Hypothetical protein.
Os09g0479400|	3.0	0.042401		Conserved hypothetical protein.
Os03g0218400|	3.0	0.023187	✓	Hexose transporter.
Os10g0541000|	3.0	0.029566		Seven transmembrane protein Mlo2.
Os12g0194900|	3.0	0.044178	✓	Amino acid carrier (Fragment).
Os01g0372400|	3.0	0.035408	✓	Glutathione S-transferase, C-terminal domain containing protein.
Os03g0802500|	3.0	0.038644	✓	AAA ATPase, central region domain containing protein.
Os07g0522500|	3.0	0.03664	✓	PDR6 ABC transporter.
Os06g0726200|	3.0	0.031959	✓	Endochitinase precursor (EC 3.2.1.14).
Os02g0216300|	3.0	0.041314		Conserved hypothetical protein.
Os02g0303000|	3.0	0.090843		Non-protein coding transcript, unclassifiable transcript.
Os01g0713200|	3.0	0.027944	✓	Beta-1,3-glucanase precursor.
Os11g0630300|	3.0	0.030882		Hypothetical protein.
Os06g0542300|	3.0	0.052843	✓	Heavy metal transport/detoxification protein domain containing protein.
Os04g0122000|	3.0	0.036494		Leucine-rich repeat, cysteine-containing type containing protein.
Os03g0793000|	3.0	0.033663		Zn-finger, A20-like domain containing protein.
Os03g0227400|	3.0	0.025975		Glycoside hydrolase, family 17 protein.
Os01g0666000|	3.0	0.034986		Lipid phosphate phosphatase 2 (EC 3.1.3.-) (AtLPP2) (Phosphatidic acid phosphatase 2) (AtPAP2) (Prenyl diphosphate phosphatase).
Os06g0726200|	3.0	0.028939	✓	Endochitinase precursor (EC 3.2.1.14).
Os02g0216300|	3.0	0.038006		Conserved hypothetical protein.
Os12g0633700|	3.0	0.055871		(No Hit)
Os01g0563000|	3.0	0.036051		Peptidylprolyl isomerase, FKBP-type domain containing protein.
Os01g0713200|	3.0	0.027401	✓	Beta-1,3-glucanase precursor.
Os05g0369100|	3.0	0.033595	✓	(No Hit)
Os11g0695000|	3.0	0.054435		(No Hit)
Os01g0946600|	3.0	0.039232		Glucan endo-1,3-beta-glucosidase GV (EC 3.2.1.39) ((1->3)-beta-glucan endohydrolase GV) ((1->3)-beta-glucanase isoenzyme GV) (Beta-1,3-endoglucanase GV).
Os01g0847100|	3.0	0.030434		Hypothetical protein.
Os01g0892300|	3.0	0.043578		Leucine-rich repeat, plant specific containing protein.

Os04g0395800|	3.0	0.080657	✓	ZIM domain containing protein.
Os06g0248600|	3.0	0.054834		Hypothetical protein.
Os10g0322200|	3.0	0.030882	✓	UDP-glucuronosyl/UDP-glucosyltransferase family protein.
Os05g0522600|	3.0	0.044774	✓	Leucine-rich repeat, plant specific containing protein.
Os09g0110700|	3.0	0.043375		(No Hit)
Os08g0544200|	3.0	0.036414		(No Hit)
Os01g0553900|	3.0	0.071277		(No Hit)
Os11g0493600|	3.0	0.043963		(No Hit)
Os05g0553800|	3.0	0.026444		Anti-silencing protein-like (Anti-silencing function 1b) (Anti- silencing factor 1-like protein).
Os07g0194500|	3.0	0.033395		2OG-Fe(II) oxygenase domain containing protein.
Os11g0172300|	2.9	0.039488		Leucine-rich repeat, plant specific containing protein.
Os08g0356800|	2.9	0.042734	✓	Plant protein of unknown function family protein.
Os04g0599000|	2.9	0.028939		Protein kinase domain containing protein.
Os08g0395800|	2.9	0.055736		Plant protein of unknown function family protein.
Os05g0232800|	2.9	0.037886		HAT dimerisation domain containing protein.
Os12g0556300|	2.9	0.049921		Hypothetical protein.
Os10g0527800|	2.9	0.070645	✓	Tau class GST protein 3.
Os10g0100500|	2.9	0.024357		Protein kinase domain containing protein.
Os07g0474500|	2.9	0.080078		(No Hit)
Os04g0450900|	2.9	0.04366		Protein kinase PKN/PRK1, effector domain containing protein.
Os03g0203700|	2.9	0.04627		Plasma membrane Ca2+-ATPase.
Os02g0561000|	2.9	0.039488		(No Hit)
Os08g0386200|	2.9	0.04247	✓	WRKY transcription factor 69.
Os10g0204500|	2.9	0.046289		(No Hit)
Os02g0703300|	2.9	0.036983	✓	Protein of unknown function DUF1218 family protein.
Os04g0121800|	2.9	0.037823	✓	Non-protein coding transcript, uncharacterized transcript.
Os04g0121800|	2.9	0.041278	✓	Non-protein coding transcript, uncharacterized transcript.
Os11g0244300|	2.9	0.049145		Non-protein coding transcript, uncharacterized transcript.
Os07g0429200|	2.9	0.089451		(No Hit)
Os06g0726200|	2.9	0.030243	✓	Endochitinase precursor (EC 3.2.1.14).
Os01g0677900|	2.9	0.038194		Conserved hypothetical protein.
Os11g0695000|	2.9	0.034154		(No Hit)
Os04g0531100|	2.9	0.045558		C2 domain containing protein.
Os02g0767200|	2.9	0.056055		Lipase, class 3 family protein.

Os02g0135200|	2.9	0.04526		Blast and wounding induced mitogen-activated protein kinase.
Os04g0599000|	2.9	0.033595		Protein kinase domain containing protein.
Os07g0538400|	2.9	0.058901		Receptor-like protein kinase 4.
Os11g0226100|	2.9	0.054638		Protein kinase domain containing protein.
Os09g0105400|	2.9	0.076114		(No Hit)
Os02g0703300|	2.9	0.046648	✓	Protein of unknown function DUF1218 family protein.
Os12g0161500|	2.9	0.052199		Hypothetical protein.
Os05g0305600|	2.9	0.028694		Disease resistance protein family protein.
Os04g0477300|	2.9	0.031074		Hypothetical protein.
Os02g0584700|	2.9	0.043599	✓	(No Hit)
Os02g0153700|	2.9	0.046529		Protein kinase domain containing protein.
Os08g0351700|	2.9	0.046127		Conserved hypothetical protein.
Os06g0214300|	2.9	0.08229		Esterase/lipase/thioesterase domain containing protein.
Os04g0258800|	2.9	0.086033		(No Hit)
Os11g0283200|	2.9	0.047788		(No Hit)
Os09g0457700|	2.9	0.02829	✓	Conserved hypothetical protein.
Os12g0537000|	2.9	0.036414		(No Hit)
Os03g0781300|	2.9	0.028939		Conserved hypothetical protein.
Os09g0330900|	2.9	0.048786		(No Hit)
Os08g0378000|	2.9	0.030434	✓	Conserved hypothetical protein.
Os10g0571600|	2.9	0.030628	✓	No apical meristem (NAM) protein domain containing protein.
Os06g0313500|	2.9	0.058084		Hypothetical protein.
Os04g0142400|	2.9	0.068807	✓	Conserved hypothetical protein.
Os05g0546400|	2.9	0.040335	✓	Conserved hypothetical protein.
Os07g0626200|	2.8	0.037886		DNA-binding SAP domain containing protein.
Os02g0685600|	2.8	0.028217		Protein phosphatase 2C-like.
Os01g0533400|	2.8	0.070603		Glycoside hydrolase, family 35 protein.
Os03g0700700|	2.8	0.039488		Lipoxygenase (EC 1.13.11.12).
Os03g0700700|	2.8	0.041061		Lipoxygenase (EC 1.13.11.12).
Os12g0129700|	2.8	0.035034		Cyclin-like F-box domain containing protein.
Os08g0457400|	2.8	0.032011	✓	Protein kinase APK1B (EC 2.7.1.-).
Os04g0368800|	2.8	0.030805	✓	EGF-like calcium-binding domain containing protein.
Os04g0186400|	2.8	0.044388		Phosphate transporter 6.
Os02g0159400|	2.8	0.036416		Hypothetical protein.
Os09g0421000|	2.8	0.089717		(No Hit)
Os05g0202800|	2.8	0.031148	✓	Plant metallothionein, family 15 protein.
Os01g0289600|	2.8	0.059264	✓	DNA-binding WRKY domain containing protein.
Os07g0134000|	2.8	0.052997		Amino acid permease 6.

Os03g0218400|	2.8	0.024357	✓	Hexose transporter.
Os03g0183500|	2.8	0.064269	✓	Protein of unknown function DUF581 family protein.
Os01g0722800|	2.8	0.055295		Dimethylmenaquinone methyltransferase family protein.
Os01g0508100|	2.8	0.081546	✓	Hypothetical protein.
Os03g0672300|	2.8	0.043224		Pyruvate kinase family protein.
Os02g0216300|	2.8	0.028939		Conserved hypothetical protein.
Os03g0182800|	2.8	0.060083		Ethylene responsive element binding factor3 (OsERF3).
Os04g0178300|	2.8	0.040085	✓	Copalyl diphosphate synthetase (Fragment).
Os02g0111500|	2.8	0.045338		Non-protein coding transcript, uncharacterized transcript.
Os03g0166200|	2.8	0.044133		Metridin-like ShK toxin domain containing protein.
Os03g0638900|	2.8	0.02822		Cyclin-like F-box domain containing protein.
Os01g0562600|	2.8	0.023187		Plant protein of unknown function family protein.
Os01g0713200|	2.8	0.028694	✓	Beta-1,3-glucanase precursor.
Os04g0630800|	2.8	0.044126		Anthocyanidin reductase.
Os10g0351200|	2.8	0.064587		Cytochrome P450 family protein.
Os03g0700700|	2.8	0.039661		Lipoxygenase (EC 1.13.11.12).
Os01g0722500|	2.8	0.036774		Protein of unknown function DUF1544 domain containing protein.
Os02g0135200|	2.8	0.025768		Blast and wounding induced mitogen-activated protein kinase.
Os05g0456900|	2.8	0.028939		Hypothetical protein.
Os08g0520600|	2.8	0.064904		Conserved hypothetical protein.
Os03g0700700|	2.8	0.03721		Lipoxygenase (EC 1.13.11.12).
Os01g0113900|	2.8	0.043375		Conserved hypothetical protein.
Os02g0135200|	2.8	0.025765		Blast and wounding induced mitogen-activated protein kinase.
Os01g0713200|	2.7	0.028694	✓	Beta-1,3-glucanase precursor.
Os01g0860500|	2.7	0.075353	✓	Chitinase (EC 3.2.1.14).
Os05g0305600|	2.7	0.028939		Disease resistance protein family protein.
Os09g0286800|	2.7	0.065393		(No Hit)
Os01g0713200|	2.7	0.026909	✓	Beta-1,3-glucanase precursor.
Os03g0218400|	2.7	0.041273	✓	Hexose transporter.
Os05g0100100|	2.7	0.057629		(No Hit)
Os01g0826400|	2.7	0.053649	✓	WRKY transcription factor 24.
Os01g0563000|	2.7	0.035596		Peptidylprolyl isomerase, FKBP-type domain containing protein.
Os01g0781200|	2.7	0.030434		Rp1-like protein.
Os11g0186800|	2.7	0.02829		Major facilitator superfamily protein.
Os04g0666800|	2.7	0.045937		Plant disease resistance response protein family protein.

Os02g0560200|	2.7	0.039733	✓	(No Hit)
Os04g0531100|	2.7	0.0379		C2 domain containing protein.
Os03g0181100|	2.7	0.043761	✓	ZIM domain containing protein.
Os07g0151200|	2.7	0.070387		General substrate transporter family protein.
Os05g0142900|	2.7	0.034884		Conserved hypothetical protein.
Os11g0229400|	2.7	0.030434	✓	RPR1.
Os12g0197700|	2.7	0.034878		XS zinc finger domain containing protein.
Os05g0172800|	2.7	0.028939		Conserved hypothetical protein.
Os02g0560600|	2.7	0.040335	✓	(No Hit)
Os03g0181100|	2.7	0.035821	✓	ZIM domain containing protein.
Os09g0438000|	2.7	0.037908		Respiratory burst oxidase protein E.
Os04g0390600|	2.7	0.040682		Oligopeptide transporter OPT superfamily protein.
Os12g0622900|	2.7	0.035225		Mov34/MPN/PAD-1 family protein.
Os01g0857400|	2.7	0.024357		Amino acid/polyamine transporter II family protein.
Os03g0288000|	2.7	0.075608	✓	Metallothionein-like protein 1 (MT-1).
Os05g0198400|	2.7	0.043009		Zinc transporter 4, chloroplast precursor (ZRT/IRT-like protein 4).
Os01g0713200|	2.7	0.030805	✓	Beta-1,3-glucanase precursor.
Os04g0176200|	2.7	0.042565		S-adenosyl-L-methionine: beta-alanine N-methyltransferase (Fragment).
Os01g0117700|	2.7	0.035602		LRK14.
Os11g0490200|	2.7	0.038644		Protein kinase domain containing protein.
Os03g0237100|	2.7	0.038644		Aldo/keto reductase family protein.
Os04g0192800|	2.7	0.033395		En/Spm-like transposon proteins family protein.
Os03g0327800|	2.7	0.043562		NAC-domain containing protein 29 (ANAC029) (NAC2) (NAC-LIKE, ACTIVATED BY AP3/PI protein) (NAP).
Os04g0407800|	2.7	0.040932		2OG-Fe(II) oxygenase domain containing protein.
Os10g0558700|	2.7	0.047906	✓	2OG-Fe(II) oxygenase domain containing protein.
Os01g0716500|	2.7	0.043948	✓	SAM (and some other nucleotide) binding motif domain containing protein.
Os01g0807000|	2.7	0.030882		Conserved hypothetical protein.
Os03g0207400|	2.7	0.039665		Protein phosphatase 2C-like domain containing protein.
Os02g0561800|	2.7	0.036382		(No Hit)
Os08g0328600|	2.7	0.042401		(No Hit)
Os07g0534300|	2.7	0.03472		Protein kinase family protein.
Os10g0337600|	2.7	0.038058		(No Hit)
Os12g0636500|	2.7	0.039488		Hypothetical protein.
Os07g0537000|	2.6	0.04216		Receptor protein kinase.
Os01g0392600|	2.6	0.043375		Hypothetical protein.
Os08g0384500|	2.6	0.034392		PDR-like ABC transporter (PDR3 ABC transporter).
Os04g0406300|	2.6	0.067671	✓	Hypothetical protein.

Os12g0221600|	2.6	0.024357		Hypothetical protein.
Os07g0635200|	2.6	0.023187	✓	E-class P450, group I family protein.
Os02g0561900|	2.6	0.040932		(No Hit)
Os02g0193200|	2.6	0.077148		Conserved hypothetical protein.
Os03g0696300|	2.6	0.038006		CCAAT-binding transcription factor, subunit B family protein.
Os12g0172400|	2.6	0.029457		Hypothetical protein.
Os07g0129200|	2.6	0.093962		PR1a protein.
Os01g0961300|	2.6	0.039488		Hypothetical protein.
Os08g0233400|	2.6	0.030987		TPR-like domain containing protein.
Os02g0561400|	2.6	0.036494		(No Hit)
Os03g0663400|	2.6	0.055753		Thaumatin-like protein.
Os05g0316200|	2.6	0.08363		Conserved hypothetical protein.
Os07g0122200|	2.6	0.034458	✓	Conserved hypothetical protein.
Os04g0608600|	2.6	0.044774		C1-like domain containing protein.
Os01g0864500|	2.6	0.047517	✓	Harpin-induced 1 domain containing protein.
Os03g0793700|	2.6	0.079832		Globulin 2 (Fragment).
Os10g0542900|	2.6	0.04638		Chitinase (EC 3.2.1.14) (Fragment).
Os07g0540800|	2.6	0.032011		Serine/threonine protein kinase family protein.
Os02g0615800|	2.6	0.069846		Protein kinase domain containing protein.
Os02g0134000|	2.6	0.039763		(No Hit)
Os01g0860500|	2.6	0.066437	✓	Chitinase (EC 3.2.1.14).
Os06g0297400|	2.6	0.039661		Hypothetical protein.
Os01g0218100|	2.6	0.039147	✓	Basic helix-loop-helix dimerisation region bHLH domain containing protein.
Os01g0946200|	2.6	0.039488		Conserved hypothetical protein.
Os09g0502200|	2.6	0.052423		Beta-1,3-glucanase homologue (Fragment).
Os02g0579800|	2.6	0.043338		Fw2.2.
Os08g0328600|	2.6	0.031737		(No Hit)
Os05g0304600|	2.6	0.034072		Linoleate:oxygen oxidoreductase (Fragment).
Os07g0626200|	2.6	0.052777		DNA-binding SAP domain containing protein.
Os05g0369300|	2.6	0.04737	✓	Conserved hypothetical protein.
Os06g0548200|	2.6	0.057522		FAD linked oxidase, N-terminal domain containing protein.
Os07g0510900|	2.6	0.039488		Multicopper oxidase, type 1 family protein.
Os04g0684900|	2.6	0.034869		Ribonuclease CAF1 family protein.
Os08g0133700|	2.6	0.052388		HGWP repeat containing protein.
Os11g0245100|	2.6	0.047573		(No Hit)
Os11g0525900|	2.6	0.059326		Exonuclease domain containing protein.
Os12g0621500|	2.5	0.046861		IRE.
Os01g0627900|	2.5	0.07414	✓	Cytochrome P450 monooxygenase CYP72A5 (Fragment).

Os04g0598800|	2.5	0.050116		Wall-associated kinase-like protein.
Os11g0477400|	2.5	0.050821		Transposase (Fragment).
Os02g0288400|	2.5	0.047155		MRP-like ABC transporter.
Os02g0225200|	2.5	0.095622		Conserved hypothetical protein.
Os05g0100100|	2.5	0.058897		(No Hit)
Os01g0588600|	2.5	0.065146		Protein of unknown function DUF572 family protein.
Os07g0604300|	2.5	0.066241		COBRA protein precursor (Cell expansion protein).
Os07g0181700|	2.5	0.074385		Guanylate-binding protein family protein.
Os12g0116900|	2.5	0.035018		Non-protein coding transcript, putative npRNA.
Os01g0885000|	2.5	0.043226		Cytochrome c.
Os01g0961000|	2.5	0.040352		Conserved hypothetical protein.
Os07g0695400|	2.5	0.028939		Spectrin repeat containing protein.
Os11g0506800|	2.5	0.028939		IQ calmodulin-binding region domain containing protein.
Os03g0832200|	2.5	0.058025		Calcium-binding protein precursor (Calreticulin).
Os05g0550300|	2.5	0.062739		Nonspecific lipid transfer protein.
Os03g0583900|	2.5	0.033435		Type III restriction enzyme, res subunit family protein.
Os03g0835300|	2.5	0.025768		Conserved hypothetical protein.
Os04g0197200|	2.5	0.047155		Protein kinase domain containing protein.
Os05g0553800|	2.5	0.030557		Anti-silencing protein-like (Anti-silencing function 1b) (Anti- silencing factor 1-like protein).
Os09g0468000|	2.5	0.038218		Multi antimicrobial extrusion protein MatE family protein.
Os03g0736000|	2.5	0.04032		NOT2/NOT3/NOT5 family protein.
Os01g0144800|	2.5	0.03989		Conserved hypothetical protein.
Os01g0176100|	2.5	0.046856		UDP-glucuronosyl/UDP-glucosyltransferase family protein.
Os03g0174900|	2.5	0.039487		Transcriptional activator php2.
Os10g0342300|	2.5	0.038154		Receptor-like protein kinase.
Os01g0778800|	2.5	0.037948		Peptidase M16, C-terminal domain containing protein.
Os02g0122600|	2.5	0.052084		Calcium-binding EF-hand domain containing protein.
Os11g0539200|	2.5	0.050311		Glycoside hydrolase, family 16 domain containing protein.
Os03g0203700|	2.5	0.025975		Plasma membrane Ca2+-ATPase.
Os05g0549800|	2.5	0.059042	✓	Pathogenesis-related transcriptional factor and ERF domain containing protein.
Os06g0714800|	2.5	0.02847		Protein of unknown function DUF581 family protein.
Os01g0382000|	2.5	0.033395	✓	Pathogenesis-related protein 1 precursor (PR-1).
Os05g0374200|	2.5	0.030882		FAD linked oxidase, N-terminal domain containing protein.
Os03g0189300|	2.5	0.064912		Conserved hypothetical protein.

Os07g0625500|	2.5	0.045929		Fimbriata-associated protein (Fragment).
Os01g0860500|	2.5	0.030557	✓	Chitinase (EC 3.2.1.14).
Os02g0216300|	2.5	0.03032		Conserved hypothetical protein.
osa-miR398b|C	2.5	0.063312		miRNA
Os03g0803900|	2.5	0.030434		Glycosyl transferase, family 31 protein.
Os10g0558700|	2.5	0.082475	✓	2OG-Fe(II) oxygenase domain containing protein.
Os09g0255400|	2.5	0.033947	✓	Indole-3-glycerol phosphate synthase, chloroplast precursor (EC 4.1.1.48) (IGPS).
Os01g0803600|	2.5	0.087126		(No Hit)
Os02g0176700|	2.5	0.077962		Potential calcium-transporting ATPase 9, plasma membrane-type (EC 3.6.3.8) (Ca(2+)-ATPase isoform 9).
Os10g0530900|	2.5	0.043176	✓	Glutathione S-transferase GST 30 (EC 2.5.1.18).
Os03g0748500|	2.5	0.027136		Flavodoxin/nitric oxide synthase domain containing protein.
Os01g0847100|	2.5	0.030557		Hypothetical protein.
Os01g0953100|	2.5	0.029161		Protein of unknown function DUF594 family protein.
Os10g0534600|	2.5	0.024357	✓	Conserved hypothetical protein.
Os02g0215900|	2.5	0.069827		Receptor kinase-like protein.
Os01g0142900|	2.4	0.032011		(No Hit)
Os10g0101000|	2.4	0.041273		N/apple PAN domain containing protein.
Os07g0481400|	2.4	0.023187		Disease resistance protein family protein.
Os03g0800000|	2.4	0.060285		Conserved hypothetical protein.
Os04g0477500|	2.4	0.049743		Glycosyl transferase, family 17 protein.
Os01g0351800|	2.4	0.028939		2OG-Fe(II) oxygenase domain containing protein.
Os08g0386200|	2.4	0.043092		WRKY transcription factor 69.
Os06g0165500|	2.4	0.039147		Protein kinase family protein.
Os03g0849800|	2.4	0.039223		Conserved hypothetical protein.
Os10g0530900|	2.4	0.042401	✓	Glutathione S-transferase GST 30 (EC 2.5.1.18).
Os09g0569900|	2.4	0.057044		Hypothetical protein.
Os12g0467200|	2.4	0.086897		Conserved hypothetical protein.
Os01g0782100|	2.4	0.05734		(No Hit)
Os04g0669700|	2.4	0.02576		Phospholipase/Carboxylesterase family protein.
Os11g0245100|	2.4	0.039488		(No Hit)
Os01g0957800|	2.4	0.074657		Cytochrome P450 family protein.
Os03g0823000|	2.4	0.055033		Resistance protein candidate (Fragment).
Os04g0594400|	2.4	0.04904		RNA-binding region RNP-1 (RNA recognition motif) domain containing protein.
Os10g0371000|	2.4	0.046648		Pollen Ole e 1 allergen and extensin domain containing protein.
Os01g0392600|	2.4	0.024357		Hypothetical protein.
Os04g0615700|	2.4	0.059448		Argonaute and Dicer protein, PAZ domain containing protein.

Os02g0517900|	2.4	0.037838		Conserved hypothetical protein.
Os09g0559500|	2.4	0.031152		(No Hit)
Os11g0116700|	2.4	0.04391		(No Hit)
Os03g0663400|	2.4	0.056371		Thaumatin-like protein.
Os06g0147400|	2.4	0.030317		Hypothetical protein.
Os07g0492500|	2.4	0.061048		Conserved hypothetical protein.
Os09g0370500|	2.4	0.059531		(No Hit)
Os01g0113500|	2.4	0.050209	✓	Protein kinase domain containing protein.
Os03g0694600|	2.4	0.034663		Hypothetical protein.
Os06g0248500|	2.4	0.08765		U box domain containing protein.
Os04g0407800|	2.4	0.0379		2OG-Fe(II) oxygenase domain containing protein.
Os09g0464100|	2.4	0.033395		Conserved hypothetical protein.
Os03g0322200|	2.4	0.044506	✓	Hypothetical protein.
Os02g0768000|	2.4	0.028939		Conserved hypothetical protein.
Os01g0681800|	2.4	0.044962	✓	Conserved hypothetical protein.
Os05g0208900|	2.4	0.045558		Conserved hypothetical protein.
Os03g0303100|	2.4	0.078941		Hypothetical protein.
Os05g0580100|	2.4	0.04663		Hypothetical protein.
Os05g0115100|	2.4	0.057529	✓	Eukaryotic DNA topoisomerases I, dispensable insert domain containing protein.
Os06g0693000|	2.4	0.051312		Protein kinase domain containing protein.
Os04g0686800|	2.4	0.037742		Nodulin-lile protein.
Os05g0410800|	2.4	0.059222		Conserved hypothetical protein.
Os06g0228200|	2.4	0.034884		Major intrinsic protein family protein.
Os03g0411800|	2.4	0.038794	✓	Zinc transporter 11 precursor (ZRT/IRT-like protein 11).
Os08g0172300|	2.4	0.04079		HAT dimerisation domain containing protein.
Os04g0280300|	2.4	0.076843		Hypothetical protein.
Os10g0337600|	2.4	0.024357		(No Hit)
Os06g0578400|	2.4	0.07321		Peptidase C48, SUMO/Sentrin/Ubl1 family protein.
Os06g0306300|	2.4	0.036179	✓	Peroxidase precursor (EC 1.11.1.7).
Os02g0241100|	2.4	0.042711		Protein kinase domain containing protein.
Os08g0475100|	2.4	0.066801		Esterase/lipase/thioesterase domain containing protein.
Os03g0577500|	2.4	0.036494		Avr9 elicitor response-like protein.
Os01g0953100|	2.3	0.050771		Protein of unknown function DUF594 family protein.
Os11g0211300|	2.3	0.028105		NBS-LRR disease resistance protein homologue (Fragment).
Os04g0122000|	2.3	0.033421		Leucine-rich repeat, cysteine-containing type containing protein.
Os06g0147200|	2.3	0.042222	✓	Hypothetical protein.
Os07g0440100|	2.3	0.077908		Endo-1,4-beta-glucanase.

Os09g0571200|	2.3	0.028052	✓	C2 domain containing protein.
Os03g0355500|	2.3	0.029493		Conserved hypothetical protein.
Os05g0479900|	2.3	0.058084		Conserved hypothetical protein.
Os10g0336400|	2.3	0.051277		(No Hit)
Os06g0627500|	2.3	0.024357		Leucine-rich repeat, plant specific containing protein.
Os09g0266300|	2.3	0.073147		Hypothetical protein.
Os02g0578100|	2.3	0.065264		Glucosyltransferase (Fragment).
Os07g0653900|	2.3	0.076295		ARM repeat fold domain containing protein.
Os03g0663400|	2.3	0.05998	✓	Thaumatin-like protein.
Os01g0160800|	2.3	0.055329	✓	Protein synthesis inhibitor II (EC 3.2.2.22) (Ribosome-inactivating protein II) (rRNA N-glycosidase).
Os06g0538400|	2.3	0.024202		Hypothetical protein.
Os07g0537000|	2.3	0.038164		Receptor protein kinase.
Os12g0277000|	2.3	0.054877		U box domain containing protein.
Os10g0558700|	2.3	0.03562	✓	2OG-Fe(II) oxygenase domain containing protein.
Os11g0675200|	2.3	0.079297		NBS-LRR type resistance protein (Fragment).
Os11g0700900|	2.3	0.051152		Class III chitinase homologue (OsChib3H-b).
Os01g0678100|	2.3	0.043323		Conserved hypothetical protein.
Os02g0174100|	2.3	0.036597		SBP domain containing protein.
Os06g0134500|	2.3	0.047059		Hypothetical protein.
Os03g0583900|	2.3	0.036218		Type III restriction enzyme, res subunit family protein.
Os06g0228200|	2.3	0.038644		Major intrinsic protein family protein.
Os10g0371000|	2.3	0.040033		Pollen Ole e 1 allergen and extensin domain containing protein.
Os04g0174200|	2.3	0.046856		Hypothetical protein.
Os01g0650200|	2.3	0.030154		Lipolytic enzyme, G-D-S-L family protein.
Os10g0444600|	2.3	0.051146		Phosphate transporter (Fragment).
Os01g0790200|	2.3	0.035225		Polyprotein.
Os08g0175700|	2.3	0.08499		Hypothetical protein.
Os01g0860500|	2.3	0.031296	✓	Chitinase (EC 3.2.1.14).
Os01g0543100|	2.3	0.033996		Peroxidase 72 precursor (EC 1.11.1.7) (Atperox P72) (PRXR8) (ATP6a).
Os06g0718400|	2.3	0.041817		(No Hit)
Os01g0742400|	2.3	0.04216	✓	Protein kinase domain containing protein.
Os06g0714800|	2.3	0.036827		Protein of unknown function DUF581 family protein.
Os01g0878400|	2.3	0.044683		Amino acid/polyamine transporter II family protein.
Os02g0736300|	2.3	0.030887		Membrane attack complex component/perforin/complement C9 family protein.
Os10g0442000|	2.3	0.039147		Receptor like protein kinase.
Os12g0277000|	2.3	0.044777		U box domain containing protein.

Os07g0488200|	2.3	0.075395		(No Hit)
Os07g0167500|	2.3	0.045874		Conserved hypothetical protein.
Os01g0860500|	2.3	0.02576	✓	Chitinase (EC 3.2.1.14).
Os01g0335700|	2.3	0.032482		Disease resistance protein family protein.
Os06g0608800|	2.3	0.067522		Copine domain containing protein.
Os03g0823000|	2.3	0.06425		Resistance protein candidate (Fragment).
Os01g0138300|	2.3	0.056154		Protein kinase domain containing protein.
Os12g0194100|	2.3	0.03416		Hypothetical protein.
Os01g0624500|	2.3	0.033395		Sgt1.
Os01g0516400|	2.3	0.06584		Conserved hypothetical protein.
Os11g0695800|	2.3	0.049123		Protein kinase domain containing protein.
Os04g0597400|	2.3	0.025886		TGF-beta receptor, type I/II extracellular region family protein.
Os07g0487100|	2.3	0.085028		UDP-glucuronosyl/UDP-glucosyltransferase family protein.
Os04g0407800|	2.3	0.065267		2OG-Fe(II) oxygenase domain containing protein.
Os06g0228200|	2.3	0.030317		Major intrinsic protein family protein.
Os08g0378000|	2.3	0.054967	✓	Conserved hypothetical protein.
Os10g0555900|	2.3	0.043874		Beta-expansin precursor.
Os03g0157700|	2.3	0.036794		Protein of unknown function DUF639 family protein.
Os02g0288000|	2.3	0.064173		Cyclin-like F-box domain containing protein.
Os02g0157200|	2.3	0.058865		Leucine-rich repeat, plant specific containing protein.
Os11g0547300|	2.3	0.048617		(No Hit)
Os03g0667100|	2.3	0.083709		BTB/POZ domain containing protein.
Os03g0293600|	2.3	0.089024		Hypothetical protein.
Os06g0608800|	2.3	0.077203		Copine domain containing protein.
Os01g0137200|	2.3	0.043176	✓	Receptor serine/threonine kinase PR5K.
Os08g0396700|	2.3	0.039488		DC1 domain containing protein.
Os03g0125100|	2.3	0.050209		Beta-carotene hydroxylase.
Os02g0658900|	2.3	0.045456		Sec20 family protein.
Os02g0180700|	2.3	0.043874		Cinnamoyl-CoA reductase (EC 1.2.1.44).
Os04g0338100|	2.3	0.046189	✓	IN2-2 protein.
Os03g0241600|	2.3	0.040394		Protein kinase domain containing protein.
Os11g0604500|	2.3	0.052143		Conserved hypothetical protein.
Os10g0337700|	2.3	0.030781		MFP1 attachment factor 1.
Os09g0325700|	2.3	0.061965		Protein phosphatase 2C (EC 3.1.3.16) (PP2C).
Os10g0372800|	2.3	0.087334		Hypothetical protein.
Os12g0580900|	2.2	0.044133		Protein phosphatase 2C-like protein.
Os04g0122000|	2.2	0.028939		Leucine-rich repeat, cysteine-containing type containing protein.

Os04g0574800|	2.2	0.073686		Dihydrodipicolinate synthase 1, chloroplast precursor (EC 4.2.1.52) (DHDPS 1).
Os01g0957200|	2.2	0.0587		Conserved hypothetical protein.
Os01g0377000|	2.2	0.045044		Cytochrome P450 family protein.
Os01g0738100|	2.2	0.045733		Peptidase C48, SUMO/Sentrin/Ubl1 family protein.
Os06g0701800|	2.2	0.045785		(No Hit)
Os11g0117500|	2.2	0.085789	✓	(No Hit)
Os11g0161700|	2.2	0.040033		NB-ARC domain containing protein.
Os11g0180200|	2.2	0.039488		Tyrosine specific protein phosphatase family protein.
Os01g0516600|	2.2	0.058956		Stable protein 1.
Os03g0411800|	2.2	0.039247	✓	Zinc transporter 11 precursor (ZRT/IRT-like protein 11).
Os03g0268400|	2.2	0.064228		Mannose-1-phosphate guanyltransferase (EC 2.7.7.13) (ATP-mannose-1- phosphate guanylyltransferase) (GDP-mannose pyrophosphorylase) (NDP- hexose pyrophosphorylase).
Os06g0711100|	2.2	0.050729		Hypothetical protein.
Os06g0557100|	2.2	0.042737		Protein kinase domain containing protein.
Os12g0174100|	2.2	0.044757		Hypothetical plant protein family protein.
Os02g0440900|	2.2	0.050268		Phosphatidylinositol transfer-like protein II.
Os06g0147200|	2.2	0.086386	✓	Hypothetical protein.
Os05g0576600|	2.2	0.04052	✓	Conserved hypothetical protein.
Os12g0637100|	2.2	0.05336		Purple acid phosphatase (EC 3.1.3.2).
Os08g0409500|	2.2	0.043129		VQ domain containing protein.
Os09g0428300|	2.2	0.065307		Metabotropic gamma-aminobutyric acid receptor, type B family protein.
Os11g0208800|	2.2	0.086081		Receptor-like protein kinase.
Os05g0198400|	2.2	0.069968		Zinc transporter 4, chloroplast precursor (ZRT/IRT-like protein 4).
Os05g0187100|	2.2	0.091202		Hexokinase.
Os06g0608800|	2.2	0.092494		Copine domain containing protein.
Os03g0816800|	2.2	0.054548	✓	Protein of unknown function DUF567 family protein.
Os01g0564000|	2.2	0.037948		(No Hit)
Os07g0617200|	2.2	0.032542		(No Hit)
Os04g0434800|	2.2	0.024211		Stromal ascorbate peroxidase.
Os01g0114600|	2.2	0.028939		Receptor-like kinase ARK1AS (Fragment).
Os07g0134000|	2.2	0.056456		Amino acid permease 6.
Os11g0588400|	2.2	0.075186		Disease resistance protein family protein.
Os10g0530900|	2.2	0.086658	✓	Glutathione S-transferase GST 30 (EC 2.5.1.18).
Os07g0631500|	2.2	0.087198		(No Hit)
Os06g0113500|	2.2	0.023187		Conserved hypothetical protein.

Os03g0241600|	2.2	0.037838		Protein kinase domain containing protein.
Os03g0571700|	2.2	0.032076		TRANSPARENT TESTA 12 protein.
Os09g0567900|	2.2	0.053192		Inosine/uridine-preferring nucleoside hydrolase domain containing protein.
Os01g0826400|	2.2	0.05127	✓	WRKY transcription factor 24.
Os01g0231300|	2.2	0.057021		(No Hit)
Os06g0235700|	2.2	0.025739		(No Hit)
Os10g0485800|	2.2	0.07245		(No Hit)
Os06g0133500|	2.2	0.042877	✓	Conserved hypothetical protein.
Os11g0227300|	2.2	0.038644		Conserved hypothetical protein.
Os09g0482900|	2.2	0.064145		UDP-glucuronosyl/UDP-glucosyltransferase family protein.
Os02g0117200|	2.2	0.028939		Hypothetical protein.
Os03g0174900|	2.2	0.042737		Transcriptional activator php2.
Os04g0434800|	2.2	0.036414		Stromal ascorbate peroxidase.
Os07g0524300|	2.2	0.072474		Non-protein coding transcript, putative npRNA.
Os01g0624500|	2.2	0.037742		Sgt1.
Os06g0339500|	2.2	0.058744		Non-protein coding transcript, unclassifiable transcript.
Os03g0276500|	2.2	0.036945		Heat shock protein 70.
Os02g0669500|	2.2	0.047542		Conserved hypothetical protein.
Os12g0182300|	2.2	0.030805		Protein kinase domain containing protein.
Os12g0162800|	2.2	0.095391		Hypothetical protein.
Os08g0562600|	2.2	0.057592		C2 domain containing protein.
Os02g0108800|	2.2	0.055736		Conserved hypothetical protein.
Os03g0405500|	2.2	0.039488	✓	PDI-like protein.
Os04g0448000|	2.2	0.038674		(No Hit)
Os10g0548700|	2.2	0.032204		Protein kinase domain containing protein.
Os05g0304600|	2.2	0.033374		Linoleate:oxygen oxidoreductase (Fragment).
Os02g0174900|	2.2	0.040033		Non-protein coding transcript, uncharacterized transcript.
Os11g0705200|	2.2	0.039733	✓	Scarecrow-like 11 (Fragment).
Os10g0128400|	2.2	0.044276		Non-protein coding transcript, uncharacterized transcript.
Os02g0281800|	2.1	0.081422		Conserved hypothetical protein.
Os08g0453400|	2.1	0.039488		(No Hit)
Os05g0507300|	2.1	0.028694		Calreticulin 3 precursor.
Os10g0548700|	2.1	0.040916		Protein kinase domain containing protein.
Os02g0555700|	2.1	0.079269		Chaperone protein dnaJ (40 kDa heat shock chaperone protein) (HSP40).
Os01g0730600|	2.1	0.046529		(No Hit)
Os01g0846400|	2.1	0.08898		Hypothetical protein.
Os08g0173300|	2.1	0.040831		(No Hit)

Os03g0835200|	2.1	0.035668		Hypothetical protein.
Os06g0163800|	2.1	0.064002		(No Hit)
Os12g0512100|	2.1	0.023187		General substrate transporter family protein.
Os03g0153500|	2.1	0.037216		Aromatic-ring hydroxylase family protein.
Os09g0353200|	2.1	0.042876		Protein kinase domain containing protein.
Os01g0840100|	2.1	0.053383		Heat shock protein 70.
Os01g0821900|	2.1	0.035021		Protein kinase domain containing protein.
Os11g0227000|	2.1	0.046066		Hypothetical protein.
Os07g0494700|	2.1	0.093572		Hypothetical protein.
Os02g0157400|	2.1	0.059527		(No Hit)
Os02g0502300|	2.1	0.068569		(No Hit)
Os05g0373900|	2.1	0.059127		Eukaryotic peptide chain release factor subunit 1 (eRF1) (Eukaryotic release factor 1).
Os10g0513900|	2.1	0.04698		E-class P450, group I family protein.
Os06g0185300|	2.1	0.050078		Transferase family protein.
Os03g0192600|	2.1	0.053575		Plant lipid transfer protein/Par allergen family protein.
Os03g0672300|	2.1	0.042629		Pyruvate kinase family protein.
Os09g0412300|	2.1	0.067118		Calcium-binding EF-hand domain containing protein.
Os04g0212400|	2.1	0.0486		(No Hit)
Os04g0430600|	2.1	0.053423		Harpin-induced 1 domain containing protein.
Os08g0556000|	2.1	0.056371		YT521-B-like protein family protein.
Os02g0215700|	2.1	0.036051		Protein kinase domain containing protein.
Os08g0556000|	2.1	0.066162		YT521-B-like protein family protein.
Os01g0949300|	2.1	0.053522		Calcium-binding EF-hand domain containing protein.
Os12g0632900|	2.1	0.043946		Receptor protein kinase.
Os08g0508800|	2.1	0.071113	✓	Lipoxygenase, chloroplast precursor (EC 1.13.11.12).
Os08g0409900|	2.1	0.064454	✓	Major facilitator superfamily protein.
Os03g0405500|	2.1	0.04638	✓	PDI-like protein.
Os01g0758300|	2.1	0.083746		Phosphoenolpyruvate carboxylase, housekeeping isozyme (EC 4.1.1.31) (PEPCase).
Os10g0200000|	2.1	0.031202		Protein kinase domain containing protein.
Os01g0730700|	2.1	0.06956		WRKY transcription factor 49.
Os07g0124100|	2.1	0.03131		Phytosulfokines 4 precursor [Contains: Phytosulfokine-alpha (PSK- alpha) (Phytosulfokine-a); Phytosulfokine-beta (PSK-beta) (Phytosulfokine-b)].
Os05g0505800|	2.1	0.034435		(No Hit)
Os12g0543600|	2.1	0.075891		Potential sarcosine oxidase (EC 1.5.3.1).
Os03g0577500|	2.1	0.03173		Avr9 elicitor response-like protein.
Os01g0758300|	2.1	0.08943		Phosphoenolpyruvate carboxylase, housekeeping isozyme (EC 4.1.1.31) (PEPCase).

Os08g0158200|	2.1	0.047517		FAD linked oxidase, N-terminal domain containing protein.
Os12g0636400|	2.1	0.05111	✓	Epoxide hydrolase family protein.
Os02g0181300|	2.1	0.066221	✓	WRKY transcription factor 71 (Transcription factor WRKY09).
Os07g0550400|	2.1	0.054354		Receptor-like protein kinase 5.
Os02g0566300|	2.1	0.076521		Conserved hypothetical protein.
Os03g0312500|	2.1	0.033996		Inhibitor of apoptosis-like protein.
Os01g0836800|	2.1	0.044838		Lung seven transmembrane receptor family protein.
Os04g0255700|	2.1	0.044133		(No Hit)
Os04g0653200|	2.1	0.04366		Low affinity calcium transporter CAX2 (Fragment).
Os10g0513900|	2.1	0.057112	✓	E-class P450, group I family protein.
Os04g0476800|	2.1	0.050207		TA5 protein (Fragment).
Os04g0209300|	2.1	0.039753		Glutathione-conjugate transporter AtMRP4.
Os04g0331900|	2.1	0.058865		Hypothetical protein.
Os06g0163900|	2.1	0.044285		Disease resistance protein family protein.
Os10g0370400|	2.1	0.043948		Disease resistance protein family protein.
Os05g0567500|	2.1	0.035034		HhH-GPD family protein.
Os04g0450400|	2.1	0.092312		Zn-finger, RING domain containing protein.
Os03g0279400|	2.1	0.044926		Arginine biosynthesis protein ArgJ family protein.
Os06g0111800|	2.1	0.087758		CSLD2 (Fragment).
Os01g0266400|	2.1	0.025768		Conserved hypothetical protein.
Os07g0271000|	2.1	0.064185		GDP dissociation inhibitor protein OsGDI1.
Os04g0633700|	2.1	0.041278		(No Hit)
Os09g0293400|	2.1	0.030557		Ubiquitin-conjugating enzyme E2.
Os01g0346600|	2.1	0.034938		Conserved hypothetical protein.
Os03g0326000|	2.1	0.049743	✓	Phospholipid-transporting ATPase 1 (EC 3.6.3.1) (Aminophospholipid flippase 1).
Os01g0706800|	2.1	0.065408		Jacalin-related lectin domain containing protein.
Os07g0260400|	2.1	0.08161		Phospholipase D.
Os04g0569300|	2.1	0.066916		Membrane protein.
Os01g0950200|	2.1	0.051349		Conserved hypothetical protein.
Os04g0373400|	2.1	0.047517	✓	Multi antimicrobial extrusion protein MatE family protein.
Os05g0467000|	2.1	0.058897		Calcium-dependent protein kinase.
Os08g0527200|	2.1	0.045904		Peptidase C19, ubiquitin carboxyl-terminal hydrolase 2 family protein.
Os01g0766300|	2.1	0.068274		Conserved hypothetical protein.
Os03g0310600|	2.1	0.083933		Plant protein of unknown function family protein.
Os08g0408500|	2.1	0.043874		Pathogenesis-related transcriptional factor and ERF domain containing protein.
Os02g0665500|	2.1	0.043121		Protein kinase domain containing protein.

Os03g0757900|	2.1	0.053875		UDP-glucose 6-dehydrogenase (EC 1.1.1.22) (UDP-Glc dehydrogenase) (UDP-GlcDH) (UDPGDH).
Os09g0323000|	2.1	0.023187		UDP-glucose 4-epimerase (EC 5.1.3.2) (Galactowaldenase) (UDP-galactose 4-epimerase).
Os05g0390600|	2.1	0.049526		Conserved hypothetical protein.
Os07g0256100|	2.1	0.057883		(No Hit)
Os02g0644400|	2.1	0.037165		Kinesin, motor region domain containing protein.
Os07g0541200|	2.1	0.035958		Protein kinase family protein.
Os04g0476800|	2.1	0.06588		TA5 protein (Fragment).
Os02g0297200|	2.1	0.046189		Protein of unknown function DUF594 family protein.
Os04g0615800|	2.0	0.02912		Stem cell self-renewal protein Piwi domain containing protein.
Os01g0778100|	2.0	0.064349	✓	(No Hit)
Os05g0387700|	2.0	0.061556		Protein kinase domain containing protein.
Os11g0155100|	2.0	0.063276		Cyclin-like F-box domain containing protein.
Os01g0551000|	2.0	0.041278		Conserved hypothetical protein.
Os09g0538600|	2.0	0.046632		Peroxisome type ascorbate peroxidase.
Os01g0631800|	2.0	0.035872	✓	Conserved hypothetical protein.
Os10g0542900|	2.0	0.044599	✓	Chitinase (EC 3.2.1.14) (Fragment).
Os10g0429700|	2.0	0.067581		Viral coat and capsid protein family protein.
Os06g0574200|	2.0	0.04216		UspA domain containing protein.
Os03g0801900|	2.0	0.035958	✓	Protein of unknown function DUF569 family protein.
Os07g0191800|	2.0	0.044152		(No Hit)
Os08g0439600|	2.0	0.097285		Plant protein of unknown function DUF868 family protein.
Os11g0691700|	2.0	0.025859		Hypothetical protein.
Os05g0553400|	2.0	0.078756	✓	Myb-related transcription factor-like protein (MYB transcription factor).
Os12g0129800|	2.0	0.041273		Hypothetical protein.
Os02g0705400|	2.0	0.039753	✓	Pathogen induced protein 2-4.
Os11g0119200|	2.0	0.033167		Cytochrome P450 family protein.
Os05g0187600|	2.0	0.075693		Peptidase aspartic family protein.
Os02g0324900|	2.0	0.034874		(No Hit)
Os04g0205200|	2.0	0.087854		Disease resistance protein family protein.
Os03g0661900|	2.0	0.030434		Peptidase, trypsin-like serine and cysteine proteases domain containing protein.
Os11g0100100|	2.0	0.030317		Autophagy 8h (Symbiosis-related like protein).
Os09g0454600|	2.0	0.076505	✓	Phosphate carrier protein, mitochondrial precursor (PTP). Splice isoform B.
Os01g0136800|	2.0	0.03556		Protein kinase domain containing protein.
Os01g0778100|	2.0	0.094584	✓	(No Hit)
Os07g0211000|	2.0	0.098661		Conserved hypothetical protein.
Os01g0213400|	2.0	0.040219	✓	Zn-finger, RING domain containing protein.

Os06g0195800	2.0	0.050352	✓	Conserved hypothetical protein.
Os10g0130600	2.0	0.059272		Nbs-lrr resistance protein (Fragment).
Os03g0269900	2.0	0.058448		Protein of unknown function DUF604 family protein.
Os02g0634700	2.0	0.075426		Serine carboxypeptidase II-1 precursor (EC 3.4.16.6) (CP-MII.1) (Fragment).
Os03g0192600	2.0	0.060875		Plant lipid transfer protein/Par allergen family protein.
Os07g0540100	2.0	0.033485		Receptor protein kinase.
Os06g0557100	2.0	0.028694	✓	Protein kinase domain containing protein.
Os08g0266400	2.0	0.044774		Leucine-rich repeat, typical subtype containing protein.
Os01g0699600	2.0	0.05152	✓	Protein kinase domain containing protein.
Os09g0361700	2.0	0.023187		Protein of unknown function UPF0203 family protein.
Os01g0827600	2.0	0.038644		Exo70 exocyst complex subunit family protein.
Os01g0117100	2.0	0.047662		LRK14.
Os01g0960400	2.0	0.099168		Protein kinase domain containing protein.
Os11g0674400	2.0	0.058084		NBS-LRR type resistance protein (Fragment).
Os07g0604300	2.0	0.054155		COBRA protein precursor (Cell expansion protein).
Os06g0253100	2.0	0.060528		Heat shock protein Hsp20 domain containing protein.
Os06g0294600	2.0	0.034986	✓	Cytochrome P450 family protein.
Os04g0112100	2.0	0.029434		Disease resistance protein family protein.
Os06g0159400	2.0	0.041283		U box domain containing protein.
Os10g0555900	2.0	0.057544		Beta-expansin precursor.
Os12g0520600	2.0	0.06434		(No Hit)
Os04g0117900	2.0	0.023187		Toc64.
Os04g0535600	2.0	0.093728		Beta-fructofuranosidase 1 precursor (EC 3.2.1.26) (Sucrose-6-phosphate hydrolase 1) (Invertase 1).
Os02g0819400	2.0	0.043578		Globin-like family protein.
Os04g0430600	2.0	0.067849		Harpin-induced 1 domain containing protein.
Os09g0517100	2.0	0.093416	✓	Disease resistance protein family protein.
Os08g0479300	2.0	0.045209		Cyclin, N-terminal domain containing protein.
