# Supplementary material for: WRKY45-dependent priming of diterpenoid phytoalexin biosynthesis in rice and the role of cytokinin in triggering the reaction
Source: Plant Mol Biol. 2014 Jul 18;86(1):171–83. doi: 10.1007/s11103-014-0221-x (PMC4133022; doi:10.1007/s11103-014-0221-x)
Supplement: Supplementary file 3 — Supplementary material 3 (PDF 25 kb) [file 11103_2014_221_MOESM3_ESM.pdf]

Table S2. List of PCR primers used for qRT-PCR.

Gene	Forward	Reverse
OsWRKY45	CGGGTAAAACGATCGAAAGA	TTTCGAAAGCGGAAGAACAG
OsNPR1	TGGCAGGTGAGAGTCTACGA	AGGTGGATTTGCACCAGAAC
OsCPS2	GGGAATCAAAGGATTCAGCA	TGTCCATGACATGAGCCACT
OsCPS4	CCATCGAGATTTTTGGAGGA	TGGAAAGTTTGCAGCAGATG
OsKSL4	ATGGGTTGCTGGTCAGGTAG	GTTCCAGCGTGGCATAAAAT
OsKSL7	CATGGTGTCACTTGGACAGG	TCAGCCTGAACAACTCATCG
OsKSL10	CTTGCTGATGCCCGAATG	CGCGATGTAGTTCTCCTTCTCTT
OsCYP71Z6	GGTTGCAGCTAAACTCATTACCC	GCATTCCATTAGGACGGATGT
OsCYP71Z7	GCTGTTCGTCCTCCTCTCCA	ATGATGGGGCCGTGCTT
OsCYP99A2	GCGAGTGTTCGACAACAAGA	GGCAACACTGGATTCAACCT
OsCYP99A3	CAACGGCGAGGAGAAAGAA	CACAGTCACACACATCCATCAAC
OsMAS	GAAGCTCGACGTCATGTTCA	GCTCGAAGTCCTCCTTGGT
OsKOL4	CATGGACGAGAAGGTGTGG	TGAACTGCATGGTGTCCTCCT
OsTGAP1	ATGGCCAGTGAAGGATGAAG	CTCTTGTGCCCACATCAGAA
OsRubq1	GGAGCTGCTGCTGTTCTAGG	TTCAGACACCATCAAACCAGA
T3A	ACATAACCAAAGAGAGGTGA	TTATGGCATTGGAAAAGCTG
OsRR6	GTGATCATGTCGTCGGAGAA	CATCTGATACGGCTGCAGAG

WRKY45-dependent priming of diterpenoid phytoalexin biosynthesis in rice and the role of cytokinin in triggering the reaction

Plant Molecular Biology,

Aya Akagi, Setsuko Fukushima, Kazunori Okada, Chang-Jie Jiang, Riichiro Yoshida, Akira Nakayama, Masaki Shimono, Shoji Sugano, Hisakazu Yamane, Hiroshi Takatsuji,

National Institute of Agrobiological Sciences,

Corresponding author: Hiroshi Takatsuji E-mail: takatsuh@affrc.go.jp
